# Convergent Astrocytic Failure in Parkinson’s Disease: A System-Level Model of Pathogenesis and Treatment

**DOI:** 10.1007/s12035-026-06177-0

**Published:** 2026-09-22

**Authors:** Ahmed M. Abdelaziz, Mohamed N. Fawzy, Mustafa M. Shokr

**Affiliations:** Department of Pharmacology and Toxicology, Faculty of Pharmacy, Sinai University – Arish Branch, Arish, 45511 Egypt

**Keywords:** Astrocyte-neuron crosstalk, Neuroinflammation, Glymphatic system, Alpha-synuclein, Parkinson’s disease pathogenesis, Glial therapeutics

## Abstract

A paradigm shift from a purely neurodegenerative disorder to a multicellular failure of the neuroglial unit has fundamentally redefined Parkinson’s disease (PD). This review synthesizes compelling evidence that positions astrocyte dysfunction, or astrocytopathy, as a central and active driver of pathogenesis, extending far beyond a passive bystander role. We delineate how pathological α-synuclein triggers a vicious cycle of astrocytic failure, encompassing impaired proteostasis via ubiquitin-proteasome and autophagy-lysosomal pathways, glymphatic system collapse due to aquaporin-4 (AQP4) depolarization, and chronic neuroinflammation driven by microglial crosstalk and NLRP3 inflammasome activation. This core triad is exacerbated by critical deficits in metabolic support, including disruption of the astrocyte-neuron lactate shuttle, aberrant lipid droplet metabolism, and failure in mitochondrial transfer. Furthermore, we integrate emerging systemic axes, demonstrating how peripheral gut dysbiosis, via the microbiota-gut-astrocyte (MGA) axis, and central circadian rhythm disruption converge to amplify central glial pathology. Critically, the present reframing unveils novel, cell-type-specific therapeutic avenues. We evaluate strategies to restore neuroprotection by targeting AQP4 polarization, modulating GLP-1 receptor signaling to mitigate inflammation, employing senolytics to clear dysfunctional glia, and utilizing gene therapy to engineer astrocytes as trophic bio-factories. By deconstructing PD as a disorder of astrocyte-neuron crosstalk, this review provides a roadmap for next-generation, disease-modifying therapies aimed at rescuing glial homeostasis to halt progression, moving beyond symptomatic dopamine replacement to address the core pathophysiology.

## Introduction

**Fig. 1:**
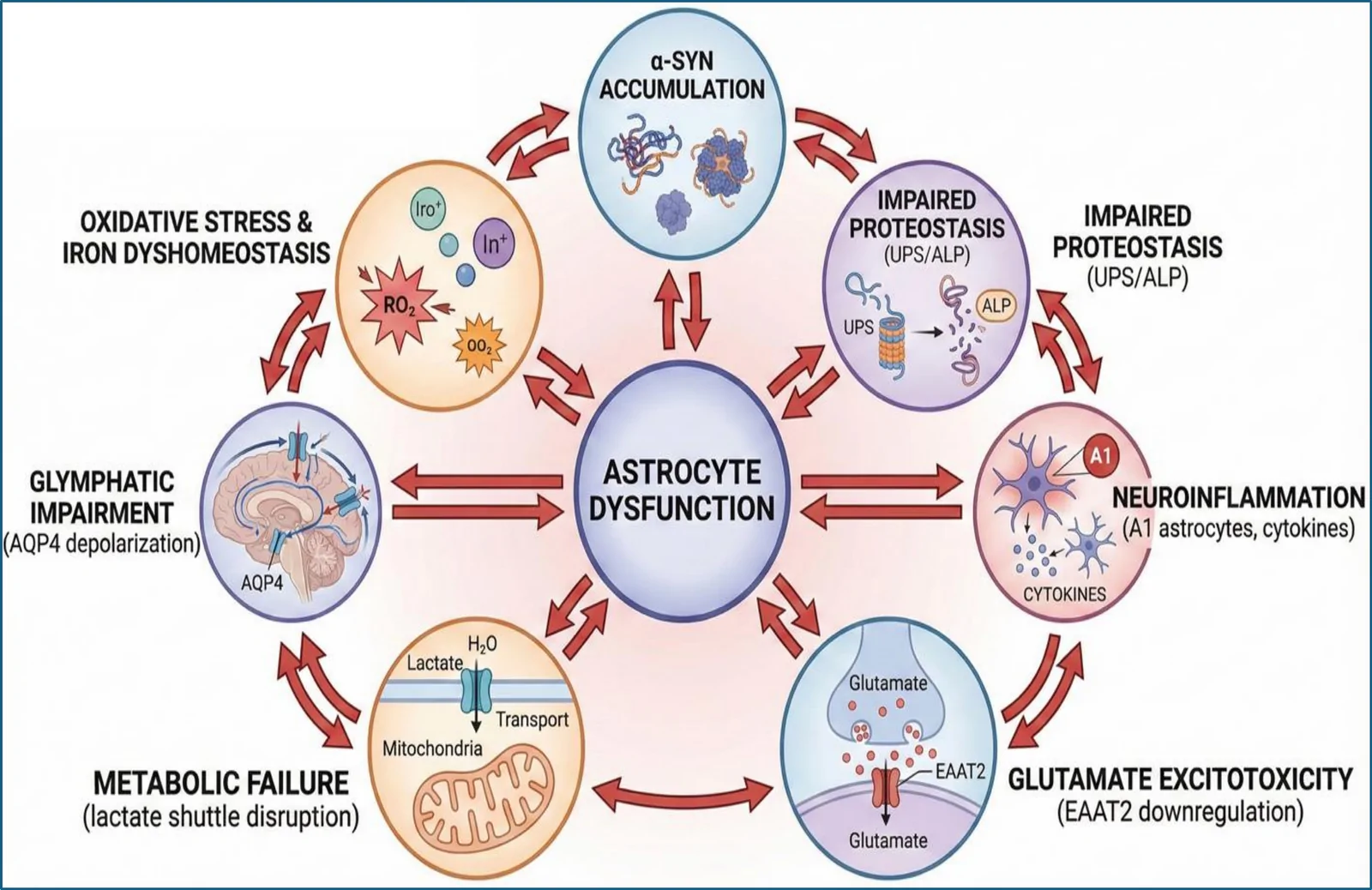
Bidirectional interplay between astrocyte dysfunction and parkinsonian neuropathology. Bidirectional interplay between astrocyte dysfunction and major pathological hallmarks of PD. Astrocyte dysfunction both responds to and amplifies multiple deleterious processes, forming a self-perpetuating cycle. While the schematic depicts established interactions in the chronic disease state, the chronological sequence of pathological events suggests that early astrocytic deficits in proteostasis and glymphatic clearance may precede and sensitize neurons to degeneration, with later-stage amplification by neuroinflammation and metabolic failure. Key pathways include accumulation of α-syn aggregates, failure of protein degradation systems (UPS/ALP), and induction of neuroinflammation via A1 astrocyte transformation. Lactate shuttle disruption, downregulation of glutamate transporter EAAT2, and loss of aquaporin-4 (AQP4) polarity compromise metabolic and homeostatic functions, leading to glymphatic impairment. These changes culminate in heightened oxidative stress and iron dysregulation, establishing a self-perpetuating cycle of neurodegeneration

The central pathological hallmark of Parkinson’s disease (PD) has long been the demise of dopaminergic neurons in the substantia nigra [1]. However, the focus is now shifting to the silent companions of these neurons: the astrocytes [2]. Once considered mere passive support cells, astrocytes are now recognized as crucial regulators of brain homeostasis, and their dysfunction, or astrocytopathy, is emerging as a critical driver of PD pathogenesis, influencing everything from protein aggregation to neuronal vulnerability [2]. Central to this dysfunction is the astrocytes’ compromised ability to manage the toxic protein α-synuclein (α-syn). In a healthy brain, astrocytes efficiently clear excess α-syn and other debris [3–5]. However, in PD, overwhelmed astrocytes not only fail to degrade these aggregates, but they may also contribute to their propagation by releasing misfolded α-syn to neighboring cells, thereby exacerbating the pathology [3–6]. Furthermore, the accumulation of α-syn within astrocytes themselves, particularly in the form of Lewy-body-like inclusions, impairs their core functions, triggering a cascade of detrimental consequences [3, 4]. One of the most critical of these is the disruption of glutamate homeostasis. Astrocytes are responsible for siphoning excess glutamate from the synaptic cleft via excitatory amino acid transporters (EAATs). In PD models, these transporters are often downregulated, leading to glutamate excitotoxicity, where overstimulation of neuronal receptors accelerates the very neurodegeneration that defines the disease [7, 8]. A profound loss of the astrocytes’ neuroprotective capacity further exacerbates this excitotoxic environment [8]. Astrocytes that are sick do not release as many neurotrophic factors like GDNF, which are important for the survival and health of dopaminergic neurons. This makes the neurons more vulnerable to other damage [9]. Concurrently, PD astrocytes adopt a reactive, often pro-inflammatory phenotype. While initially a protective response, this chronic reactivity leads to the sustained release of cytotoxic cytokines such as TNF-α and IL-1β, creating a neuroinflammatory milieu that perpetuates neuronal damage and contributes to the non-cell-autonomous mechanisms of neurodegeneration [10, 11]. This dysfunction extends to the metabolic realm, where astrocytes fail to provide adequate energetic and antioxidant support. Impairments in lactate shuttling and glutathione production render neurons more vulnerable to the relentless oxidative stress that characterizes the PD brain [12–14]. Finally, astrocyte end-feet dysfunction can compromise blood-brain barrier integrity, potentially allowing peripheral inflammatory cells and toxins into the brain parenchyma and initiating or amplifying the degenerative process [15]. Thus, astrocyte dysfunction in PD is not a mere bystander effect but an active and integral component of the disease process, creating a hostile microenvironment that facilitates protein aggregation, exacerbates neuroinflammation, and ultimately seals the fate of vulnerable neuronal populations. This paradigm shift reframes PD not solely as a neuronal disorder but as a multicellular failure, opening new therapeutic avenues aimed at restoring astrocytic neuroprotection to slow or halt disease progression [15, 16]. A critical question arises from this astrocyte-centric model: does astrocyte dysfunction precede and actively drive neuronal degeneration, or is it merely a reactive consequence of neuronal injury? While definitive chronological evidence in humans remains challenging to obtain, several lines of investigation support a primary, upstream role for astrocytopathy [17]. First, postmortem studies of incidental Lewy body disease (presymptomatic PD) have identified astrocytic α-synuclein pathology in the absence of overt neuronal loss in some brain regions, suggesting that glial dysfunction may precede neuronal death [18–20]. Second, longitudinal studies in genetic PD models like LRRK2 and GBA1 mutation carriers demonstrate that astrocytic metabolic and inflammatory alterations manifest years before detectable dopaminergic terminal loss [21–23]. Third, toxin-induced models (MPTP, 6-OHDA) reveal that astrocyte reactivity and AQP4 depolarization occur within hours of insult, while significant neuronal death requires days to weeks [24, 25]. Nevertheless, the field acknowledges significant bidirectional crosstalk: once initiated, astrocytic failure and neurodegeneration engage in mutually reinforcing cycles, as illustrated in Fig. 1. Thus, we propose that astrocyte dysfunction operates as an early amplifier and perpetuator of pathology, positioned upstream of the final common pathway of neuronal death, rather than as the sole initiator. This nuanced chronology does not diminish astrocytes as therapeutic targets; rather, it suggests that interventions restoring glial homeostasis may interrupt the feed-forward loop at a critical proximal node.

Despite this, PD has long been characterized by the degeneration of dopaminergic neurons in the substantia nigra. However, emerging research underscores astrocytes, the most abundant glial cells in the central nervous system, as active contributors to the disease pathogenesis rather than passive bystanders. Once considered merely supportive, astrocytes are now recognized to play complex roles in neuroinflammation, metabolic support, and protein clearance, with their dysfunction implicated in multiple facets of PD progression. This review synthesizes the state-of-the-art evidence linking astrocyte dysfunction to PD pathology, proposing a convergent model wherein glymphatic failure, metabolic collapse, gut dysbiosis, and circadian disruption are not isolated events but mutually reinforcing failures of the astrocyte. We dissect the molecular mechanisms of astrocytic failure across ten critical domains, from established pathways like inflammation and glutamate dysregulation to emerging frontiers such as mitochondrial transfer, lipid droplet metabolism, and circadian rhythm disruption. Finally, we critically evaluate the therapeutic landscape, assessing how targeting astrocyte-specific pathways, such as AQP4 polarization or GLP-1 signaling, offers a promising new frontier for disease-modifying interventions.

## Neurobiological Substrates of Astrocyte Dysfunction in Parkinsonian Pathogenesis

### Neuroimmune Circuitry and Glial Inflammatory Signaling Cascades

A self-amplifying glial loop drives neurodegeneration in PD. Microglia activated by α‑synuclein aggregates, complement opsonins, or extracellular ATP secrete IL‑1α, TNF‑α, C1q, and mitochondrial fragments, which together polarize astrocytes into a neurotoxic A1 state [26, 27]. These reactive astrocytes lose supportive functions and release further pro‑inflammatory mediators (IL‑1α, IL‑1β, IL‑6, and TNF‑α), largely through pattern‑recognition receptor signaling. TLR2 promotes α‑synuclein uptake and NF‑κB/p38‑mediated inflammation, while TLR4, highly expressed in human substantia nigra but apparently absent in rodent astrocytes, highlights a translational gap that demands validation in human iPSC‑derived cells [28–30]. Astrocytic inflammation is also fueled by NLRP3 inflammasome‑derived IL‑1β, triggered especially by microglial handling of α‑synuclein [31, 32] and by the accumulation of α‑synuclein within astrocytes themselves, which disrupts anti‑inflammatory pathways governed by Nurr1 and β‑arrestin2‑dependent dopamine D2 receptor signaling [32–34].

Parallel disease‑accelerating circuits link the two cell types. Complement components C1q and C3 tag distressed synapses for microglial phagocytosis, while impaired neuronal fractalkine (CX3CL1) signaling to microglial CX3CR1 removes a critical off‑signal, permitting unchecked activation [35–38]. Simultaneously, ATP released from injured neurons binds P2X7 receptors on microglia and astrocytes, driving NLRP3 inflammasome assembly and maturation of IL‑1β and IL‑18 [39–42]. These pathways interconnect: complement‑ and ATP‑stimulated microglia release the A1‑inducing cytokine cocktail, and A1 astrocytes reciprocally enhance microglial activation, generating a vicious cycle of chronic inflammation and oxidative stress that preferentially destroys dopaminergic neurons [43, 44]. In this milieu, α‑synuclein‑laden astrocytes can adopt an antigen‑presenting phenotype, upregulating MHC‑II and activating CD4⁺ T cells; high‑molecular‑weight α‑synuclein fibrils drive MHC‑II‑dependent peptide presentation, and α‑synuclein/MHC‑II complexes can spread between astrocytes via tunneling nanotubes [45, 46]. TNF‑α and α‑synuclein fibrils appear to compete in directing astrocytes toward either a pro‑inflammatory or an antigen‑presenting state [47].

**Fig. 2:**
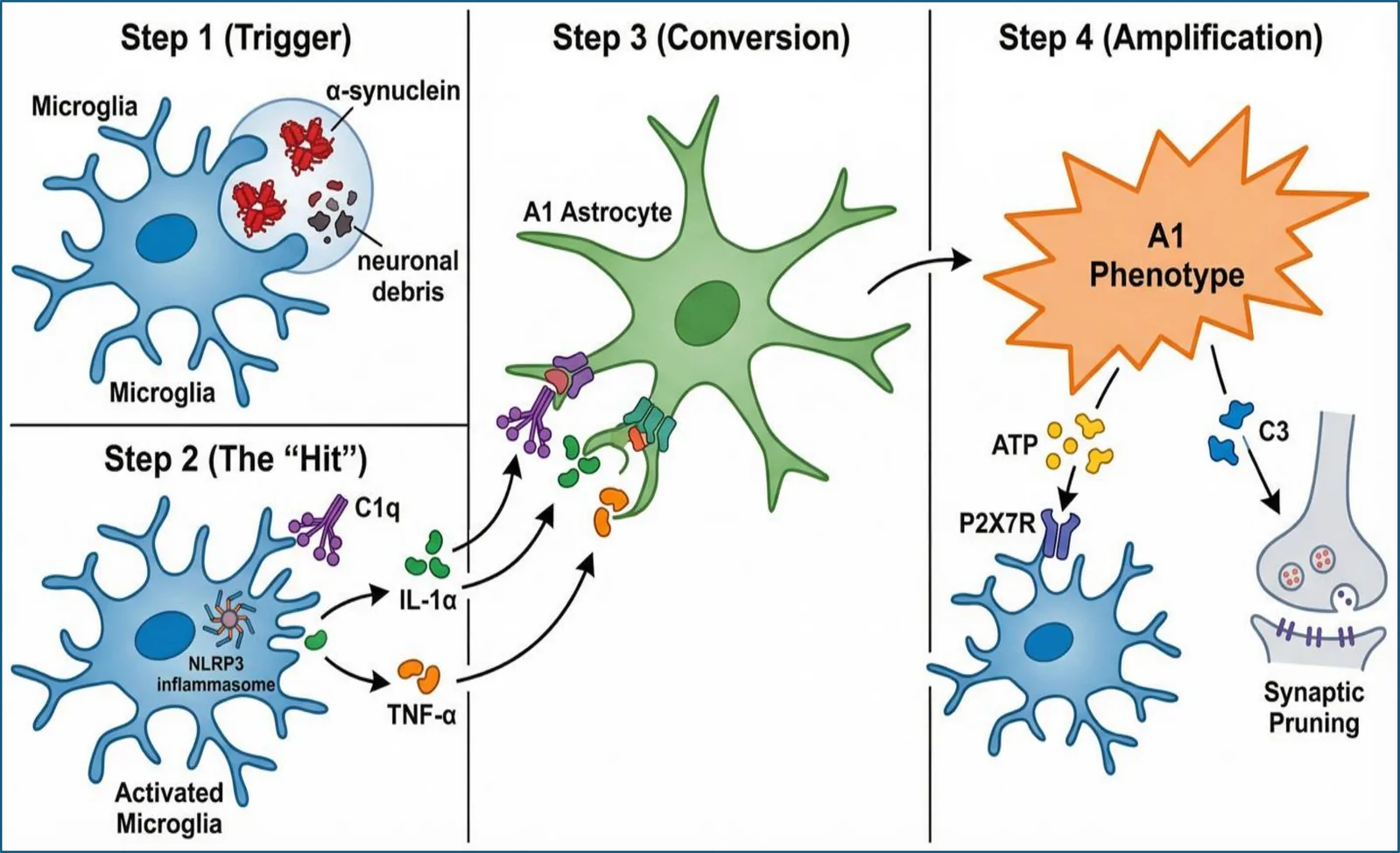
Microglia-mediated conversion of astrocytes to a neurotoxic A1 phenotype. The stepwise neuroinflammatory pathway triggered by pathological α-syn and neuronal debris. The cascade is initiated by microglial activation, which triggers the release of pro-inflammatory factors, including C1q, IL-1α, and TNF-α, and activates the NLRP3 inflammasome. This, in turn, converts resting astrocytes into harmful A1 astrocytes. These A1 astrocytes then exacerbate inflammation through ATP-P2X7R signaling and C3 release, resulting in the excessive elimination of synapses

Genetic modifiers fine-tune these responses. DJ-1 (PARK7) deficiency enhances astrocytic production of COX-2 and IL-6 while diminishing anti-inflammatory prostaglandin D through unchecked STAT1 signaling [48–51]. Importantly, DJ-1 deficiency in astrocytes promotes neuroinflammation through α-synuclein-independent mechanisms, as DJ-1 directly regulates STAT1 signaling and mitochondrial function [51, 52]; astrocytes from PARK7 mutation carriers exhibit elevated COX-2 and IL-6 without exogenous α-synuclein exposure, though α-synuclein amplifies this inflammatory state, indicating that genetic and proteinopathic triggers engage overlapping but distinct molecular pathways [29, 44]. Conversely, DJ-1 overexpression attenuates dopaminergic loss and neuroinflammation in vivo [49, 53]. Disrupting any node of this glial network, blocking P2X7R, inhibiting complement C3, or restoring fractalkine-mediated microglial quiescence, has been shown to dampen the entire loop, protect substantia nigra neurons, and improve motor outcomes in PD models, underscoring the profound therapeutic potential of targeting this pathological intercellular dialogue (Fig. 2) [42, 54].

### Disruption of Proteostatic Networks and Alpha-Synuclein Handling

#### Intracellular Proteolytic Systems: Ubiquitin-Proteasome and Autophagy-Lysosomal Pathways

The hallmark buildup of faulty proteins observed in neurodegenerative conditions such as PD is largely driven by failures in astrocytic breakdown machinery [55]. Two primary housekeeping systems, the ubiquitin-proteasome system (UPS) and the autophagy-lysosomal pathway (ALP), coordinate the salvage or elimination of aberrant polypeptides within these glial cells [56, 57]. Should these pathways become compromised, protease-resistant, malformed proteins inevitably amass.

Within the PD landscape, α-syn stands out for its ability to migrate from neurons into the astrocytic network and spread between neighboring astrocytes [58]. Notably, these glia exhibit a remarkable proficiency in dismantling captured α-syn, directing oligomeric and fibrillar species toward lysosomal compartments (marked by LAMP1) for enzymatic breakdown [59, 60]. Astrocytes actually outperform neurons in this clearing task, relying on both the ALP and UPS; pharmacological blockade using bafilomycin A1, MG132, or 3-methyladenine leads to a rise in detergent-insoluble α-syn [61–63], whereas agents such as ginkgolide B and bilobalide significantly boost its removal [64]. Such vigorous degrading activity positions astrocytes as protectors of neuronal health; indeed, when co-cultured, they lower α-syn burdens in neurons and curtail its propagation between them [65]. Furthermore, astrocytes join forces with microglia in a biphasic clean-up strategy [43, 44]. Although astrocytes are comparatively weaker decomposers, they offload a substantial fraction of internalized α-syn to microglia via secretory routes or tunneling nanotubes (TNTs); microglia, conversely, excel at destruction and seldom shuttle the protein back [66].

The neuroprotective facade of astrocytes crumbles once the influx of α-syn assemblies outstrips their disposal capabilities, resulting in the formation of persistent intracellular aggregates [67]. Both laboratory experiments and animal models confirm that α-syn inclusions can endure within astrocytes for prolonged durations [68]. This breakdown in clearance is sometimes precipitated by factors like αB-crystallin, a small heat shock protein that effectively suppresses autophagic activity in these cells [68]. Given that αB-crystallin is overexpressed in PD patients and animal models, it fosters the buildup of α-syn; moreover, autopsy examinations frequently reveal α-syn-positive inclusions within astrocytes, with their emergence closely mirroring the clinical advancement of PD [68]. Under such duress, astrocytes might extend TNTs to dispatch α-syn to neighboring healthy astrocytes, a maneuver that may initially aim to relieve cellular stress, yet ironically risks amplifying pathological spread [66]. Although intercellular shuttling from astrocytes back to neurons is uncommon, it is potent enough to trigger neuronal demise [58]. Additionally, specific genetic aberrations further compromise protein catabolism within astrocytes, intensifying α-syn aggregation [69]. The subsequent table details how distinct gene mutations disrupt astrocytic α-syn clearance, thereby fueling the pathogenic cascade of PD **[**Table 1**]**.

**Table 1:** Genetic determinants of impaired astrocytic alpha-synuclein clearance

Gene	Normal function/association	Impact on astrocyte function and α-syn clearance	Key consequences and interactions	References
LRRK2 (PARK8)	Encodes a multifunctional kinase	• Mutations (e.g., G2019S) cause lysosomal enlargement and impaired autophagy • Reduces degradation capacity • Impairs clearance of extracellular α-syn aggregates via downregulation of matrix metallopeptidase 2 and annexin A2 deficits	• Progressive α-syn accumulation • Transfer of α-syn to healthy neurons, compromising their survival • Crosstalk: Upregulates ATP13A2 as a potential compensatory mechanism	[70–72]
GBA1	Encodes the lysosomal enzyme glucocerebrosidase (GCase), a major PD risk factor	• Evidence is mixed: some studies show increased α-syn aggregation after uptake, while others find no impairment in the degradation rate of exogenous α-syn • GCase deficiency may not be sufficient alone but exacerbates pathology with other insults	• Can lead to increased α-syn aggregation • Crosstalk: Downregulates LRRK2 kinase activity; inhibiting LRRK2 can partially normalize lysosomal defects	[73–75]
ATP13A2 (PARK9)	Encodes a lysosomal ATPase	• Loss of function increases lysosomal membrane permeabilization • Impairs α-syn internalization and degradation • Promotes neuroinflammation via the NLRP3 inflammasome	• Reduces astrocytes’ ability to protect neurons from α-syn accumulation and propagation	[76, 77]

Although the abovementioned mechanisms are related to α-synuclein pathology, astrocyte dysfunction in genetic forms of PD could involve both α-synuclein-dependent and α-synuclein-independent pathways [70, 78, 79]. LRRK2 mutations like G2019S cause primary lysosomal enlargement and defective autophagic flux in astrocytes, facilitating α-synuclein accumulation [70], but LRRK2 also directly impairs AQP4 polarization and glymphatic function, independent of α-synuclein aggregation [78, 80]. PRKN/PINK1 mutant astrocytes exhibit mitochondrial fragmentation and defective mitophagy that impair their ability to support neurons independent of α-synuclein accumulation, with exposure to α-synuclein exacerbating these deficiencies [81–86]. GBA1 mutations reduce lysosomal glucocerebrosidase activity, thereby decreasing α-synuclein degradation and at the same time induce ER stress and neuroinflammation by α-synuclein-independent mechanisms [87–89]. Astrocytopathy in genetic PD thus exemplifies convergent failures, in which primary genetic mutations and secondary α-synuclein pathology are mutually exacerbating, but each of them is capable of inducing dysfunction independently of a mandatory α-synuclein intermediary [5, 78, 90].

Taken together, these genetic findings underscore the pivotal importance of lysosomal activity within astrocytes. The interplay among these pathways points to convergent pathogenic processes that ultimately disrupt α-synuclein degradation and accelerate disease advancement.

### Glymphatic Dysfunction and Aquaporin-4 Mislocalization in Perivascular Clearance

Although the UPS and autophagic machinery manage α-syn within the cellular milieu, the removal of its extracellular soluble forms depends just as crucially on the glymphatic pathway [91]. This network operates via paravascular CSF inflow and venous interstitial outflow, effectively performing a nightly brain cleanse that expels metabolic byproducts, including extracellular α-syn [92, 93]. Astrocytes are the chief coordinators of this process, as their perivascular end-feet are densely populated with AQP4 water channels that mediate the bulk parenchymal fluid movement [92].

Pathological alterations in this clearance system are a hallmark of PD, largely stemming from the mislocalization of AQP4 [94, 95]. In healthy states, AQP4 retains a strict polar orientation on astrocytic end-feet encircling vessels, preserved through anchoring connections with α-syntrophin in the dystrophin-associated protein complex [95, 96]. Conversely, PD pathology disrupts this polarization, prompting AQP4 to disperse throughout the cell body, a state defined as AQP4 depolarization. Experimental evidence shows that AQP4 knockout or depolarization hampers α-syn elimination, leading to heightened parenchymal accumulation, accelerated Lewy body genesis, and intensified dopaminergic neuronal death [95, 96]. Autopsy findings corroborate these findings, revealing that cortical α-syn burdens in PD patients are inversely proportional to AQP4 expression, with the heaviest aggregate loads appearing in areas exhibiting AQP4 depolarization [95, 96]

**Fig. 3:**
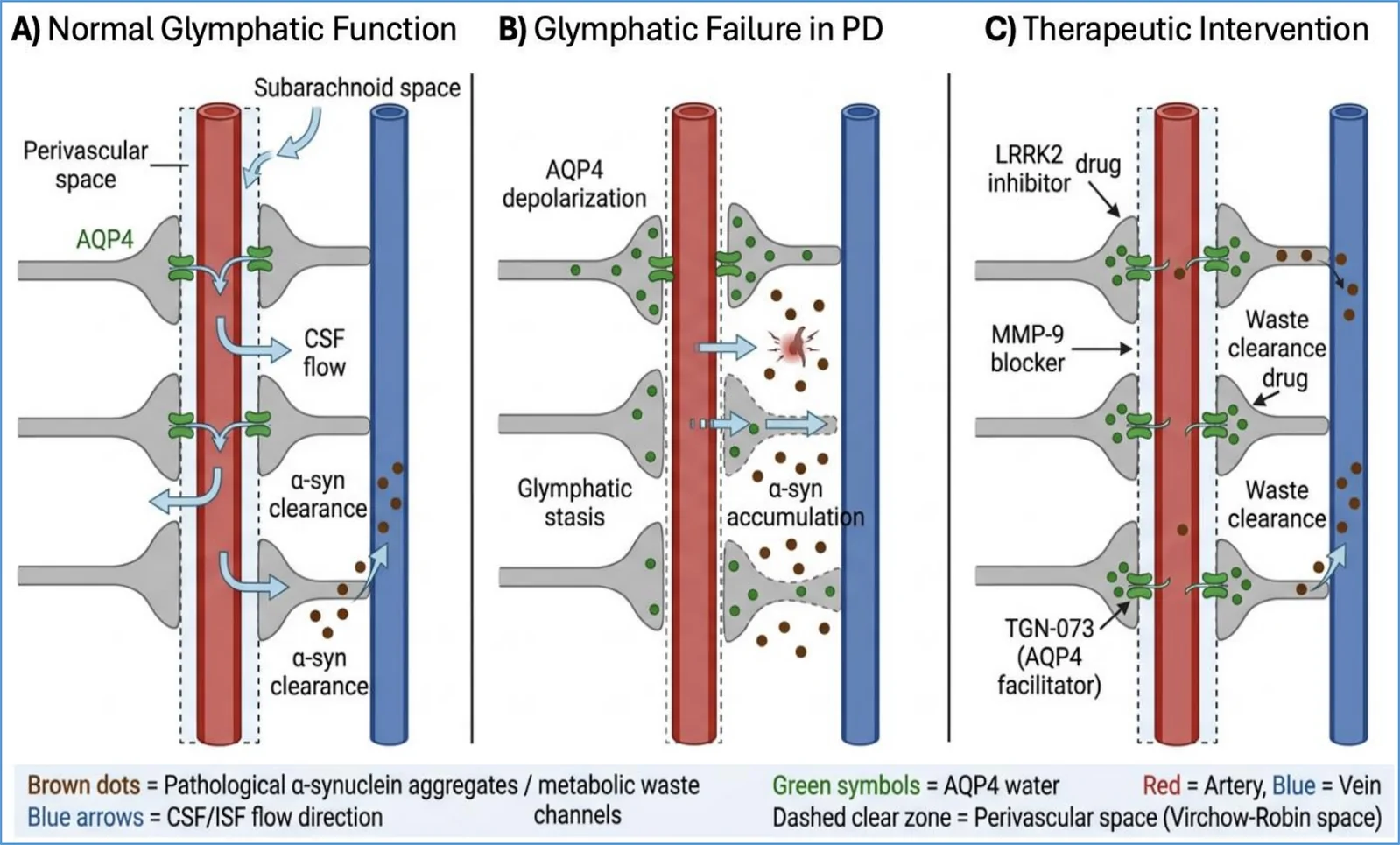
Glymphatic function, dysfunction in PD, and therapeutic restoration. The figure illustrates the role of the glymphatic system in brain clearance. **A** Under normal conditions, CSF flows along perivascular spaces, facilitated by AQP4 water channels. This directional flow clears metabolic waste, including α-synuclein, preventing pathological aggregation. **B** In glymphatic failure characteristic of PD, AQP4 depolarization disrupts CSF/ISF exchange, leading to α-syn stasis, accumulation of toxic aggregates, and impaired waste clearance. **C** Therapeutic strategies aimed at restoring glymphatic function include LRRK2 inhibitors, MMP-9 blockers, and waste-clearance drugs, which promote the removal of pathological α-synuclein and debris around arterial and venous vessels

A vicious cycle is initiated by glymphatic failure; diminished α-syn removal fosters the accumulation of extracellular aggregates, which in turn disrupts astrocyte function and AQP4 polarization, thereby amplifying the clearance deficit [96, 97]. Notably, this dysfunction manifests at the earliest stages of the neurodegenerative process, often before significant neuronal death becomes evident, thereby marking it as a promising diagnostic indicator and a point of pharmacological intervention [96]. A reciprocal interdependence defines the relationship between α-syn pathology and AQP4. When pathogenic α-syn isoforms, notably the A53T mutant, are overexpressed, a direct downregulation of AQP4 expression and polarization occurs; in a reciprocal manner, the genetic ablation of AQP4 worsens α-syn pathology, thus affirming the two-way nature of this pathogenic interaction [98]. From a clinical perspective, these findings carry considerable weight. Strategies aimed at reinstating AQP4 polarity and promoting glymphatic transit, including sleep optimization, exercise regimens, or stabilizing AQP4 at astrocytic end-feet via pharmacology, hold the potential to decelerate illness advancement by re-engaging the brain’s inherent waste-removal systems [92, 93]. Experimental genetic models of PD further support this approach, as targeting LRRK2 with pharmacological inhibitors reinstates AQP4 polarity, bolsters glymphatic clearance, and curtails the IFNγ-mediated neuroinflammatory response [99]. This LRRK2-mediated AQP4 dysregulation operates independently of α-syn pathology, as LRRK2 directly phosphorylates AQP4, causing its mislocalization from astrocyte end-feet even in the absence of α-synuclein aggregates [80, 100]. Conversely, in sporadic PD and α-synuclein-overexpressing models, AQP4 depolarization occurs secondary to α-synuclein-induced MMP-9 activation and β-dystroglycan cleavage, demonstrating that glymphatic failure can arise through both α-synuclein-dependent (sporadic PD) and α-synuclein-independent (LRRK2-PD) mechanisms converging on the same terminal phenotype [25, 96]. In MPTP models, MMP-9-mediated cleavage of β-dystroglycan disrupts the anchoring of AQP4 in the vascular basement membrane; blocking MMP-9 prevents this degradation, thereby maintaining AQP4 polarization and glymphatic function [101] (Fig. 3).

### Metal Homeostasis, Oxidative Vulnerability, and Ferroptotic Susceptibility

#### Iron Dysregulation and Ferroptosis-Prone Cellular States

Astrocytes are central to brain iron homeostasis, as they express nearly all known iron transporters and proteins involved in iron metabolism, thereby regulating its uptake, storage, and release [102]. However, this critical role can be compromised with aging, as astrocytic and microglial senescence disrupts metal homeostasis, increasing the brain’s susceptibility to neurodegenerative diseases [103, 104]. The contribution of astrocytes to disturbed iron metabolism in PD is an active area of research.

Mechanistically, while extracellular α-syn alone does not alter iron-related protein expression in astrocytes, it has a significant effect under conditions of iron overload. In such states, a marked decrease in the hepcidin-to-ferritin ratio facilitates an iron-releasing phenotype, which may be detrimental to neighboring neurons [105, 106]. Although astrocytic hepcidin does not respond to α-syn, it can regulate systemic iron intake at the blood-brain barrier by controlling ferroportin in endothelial cells [107]. The therapeutic potential of modulating hepcidin is highlighted by studies showing that its overexpression in astrocytes reduces brain iron, oxidative stress, and pathology in an Alzheimer's disease mouse model [108], and similar overexpression in neurons and glia protects against dopaminergic neurodegeneration in PD models [109]. This suggests that elevated astrocytic hepcidin may restrict peripheral iron transport, alleviating brain iron accumulation and PD symptoms.

Further evidence for the therapeutic modulation of astrocytic iron metabolism comes from studies on estrogen, which may contribute to the lower risk of PD in females [110]. Estrogen enhances the expression of the iron exporters FPN and DMT1 in astrocytes, potentially facilitating the discharge of excess iron from the brain [110, 111]. This presents a complex question of whether to suppress astrocytic iron transport to limit import or enhance it to facilitate export, underscoring that astrocytic iron regulation and its potential disturbance in PD are worthy of further investigation.

Genetic factors also play a key role. The HFE H63D variant, a PD disease modifier, promotes brain iron overload and oxidative stress [112, 113]. Interestingly, astrocytes with this variant develop adaptive mechanisms, including increased iron storage in L-ferritin and Nrf2 system activation, which limit senescence and provide neuroprotection against stressors like paraquat [114, 115]. Supporting the importance of genetics, gene expression correlating with cortical iron deposition in PD patients is predominantly found in astrocytes and glutamatergic neurons [116], indicating that the role of genetic modulations in astrocytic iron homeostasis warrants future attention.

#### Mitochondrial and Endoplasmic Reticulum Stress in Reactive Astrocytosis

Astrocytes consume 20% of brain oxygen via mitochondrial oxidative phosphorylation, not glycolysis, making mitochondrial integrity vital for ATP and normal physiology [117]. In disease, activation coincides with failure, causing calcium dysregulation, ROS, and death [118]. Internalized α-syn oligomers, but not other forms, target mitochondria, lowering oxygen consumption, disrupting fission–fusion, reducing ATP, and inducing fragmentation; these effects are reversible by oligomer-selective antibodies [119] [120, 121]. Fibrils and trans-Golgi aggregates similarly impair respiration, as confirmed in transgenic models [119, 122].

Astrocytes neuroprotect by shedding vesicles with functional mitochondria and ATP that neurons internalize to survive energy stress and stroke [123]. In PD, defective astrocytes fail to support high-demand neurons, whereas co-cultures show astrocytic mitochondria restore neuronal ATP via phospho-p38 [124, 125]. Although α-syn undermines this support, healthy astrocytes transfer mitochondria to stressed counterparts via nanotubes [126, 127]. Conversely, astrocytes activated by microglial dysfunctional mitochondria may release neurotoxic mitochondria that suppress neuronal respiration; whether α-syn prompts similar release is unknown [128].

α-Syn oligomers initially activate mitophagy but then arrest it, causing fragmented mitochondrial accumulation and failed quality control [129, 130]. Kir6.1 deletion inhibits astrocytic mitophagy, worsening dopaminergic loss, but restoring mitophagy rescues it [131, 132]. Astrocytes also perform transmitophagy, clearing damaged neuronal mitochondria to avert toxic release [133–135]. Critically, astrocytic mitochondrial dysfunction in PRKN/PINK1-mutant PD occurs independently of α-syn aggregation, as evidenced by mitochondrial fragmentation and reduced respiratory capacity in patient-derived astrocytes lacking α-synuclein pathology; however, α-synuclein exposure synergistically exacerbates these deficits, indicating that genetic and proteinopathic insults converge on shared mitochondrial pathways while remaining capable of initiating dysfunction autonomously [136–138].

Critically, astrocytic mitochondrial dysfunction in α-syn uptake/overexpression induces astrocytic ER stress, causing Golgi fragmentation, CHOP apoptosis, and increased luminal pressure [139–141]. This drives inflammation via PERK/JAK1/STAT3, upregulating IL-6/chemokines that activate microglia in a self-perpetuating loop and shift astrocytes to a reactive state with reduced synaptogenic factors [142, 143]. Mild ER stress can be protective, but transmissible ER stress is debated [144, 145]. In genetic PD, LRRK2-G2019S aggravates α-syn-induced calcium-mediated ER stress and astrocyte death, whereas Parkin blunts ER stress by degrading NOD2 [139, 146, 147]. PLA2G6 mutations cause chronic ER stress, though their α-syn interaction in astrocytes is unexplored [148, 149].

### Synaptic Dysregulation and Trophic Factor Deprivation

In PD, astrocyte dysfunction simultaneously drives excitotoxic damage and deprives neurons of essential trophic support. Healthy astrocytes clear over 90% of synaptic glutamate via EAAT2 transporters, ensuring precise neurotransmission [7, 150–153]. A toxic combination of oxidative stress, microglial inflammation, and α-synuclein pathology impairs these transporters, leading to synaptic glutamate accumulation that overstimulates NMDA and mGluR5 receptors, triggering massive Ca^2^⁺ influx and release from intracellular stores [154, 155]. The resultant calcium overload cripples mitochondria, generates reactive oxygen species, and activates calpains and caspases, pushing substantia nigra dopaminergic neurons toward apoptotic or necrotic death and accelerating motor decline [156, 157]. Dual‑pronged therapeutic strategies attempt to restore glutamate clearance by upregulating GLT‑1 such as ceftriaxone [158] or to protect neurons with mGluR5 antagonists [159, 160], reflecting a paradigm shift toward astrocyte‑targeted neuroprotection. Concurrently, the trophic axis fails: astrocytes normally supply GDNF, BDNF, and TGF‑β and sustain Wnt/β‑catenin signaling vital for dopaminergic survival and plasticity [161–164]. Early compensatory hypertrophy transiently boosts trophic factor and antioxidant output [165–167], but chronic α‑synuclein spread, escalating inflammation, and metabolic burden eventually force a switch to a neurotoxic reactive state in which GDNF, BDNF, and TGF‑β production plummets, Wnt/β‑catenin signaling is disrupted, and pro‑inflammatory mediators are released [164, 166–168]. This loss of trophic support dramatically accelerates nigrostriatal degeneration [169]. Clinical translation of trophic replenishment via GDNF gene therapy or intraputaminal infusion has yielded inconsistent results, constrained by limited parenchymal diffusion, the need for early intervention, and an insufficiently supportive tissue environment [170–176]. Effective disease modification will therefore demand a precision approach that integrates glial restoration, optimally timed trophic factor delivery, and multimodal combination therapy to halt PD progression.

### Metabolic Reprogramming and Lipidomic Alterations

#### Astrocyte-Neuron Metabolic Coupling and Energetic Failure

At the core of maintaining cerebral homeostasis lies a finely tuned metabolic alliance between astrocytes and neurons, the deterioration of which constitutes a major pathway toward the neurodegeneration observed in PD [177]. Orchestrating this symbiosis is the astrocyte-neuron lactate shuttle (ANLS), wherein astrocytes metabolize glucose into lactate in reaction to synaptic firing [178]. Following its synthesis, this lactate is shuttled outward through specific monocarboxylate transporters (MCT1 and MCT4), only to be taken up by neurons via MCT2 [179]. Once internalized, neurons, particularly those with high synaptic energy demands, preferentially oxidize this lactate to fuel mitochondrial ATP generation and uphold critical physiological activities [180, 181]. However, in the context of PD, this carefully orchestrated support network undergoes a profound breakdown. The shuttle mechanism falters owing to the diminished expression or impaired function of MCTs, while astrocytic glycogen stores, which serve as an essential reserve during metabolic emergencies, become exhausted, thereby rendering the brain exceptionally susceptible to energetic stressors [118, 181]. Additionally, intrinsic mitochondrial dysfunction within astrocytes curtails their ability to perform both bioenergetic and neuroprotective duties. Together, this destructive trio, compromised lactate transport, glycogen loss, and astrocytic mitochondrial failure, precipitates a severe shortfall in neuronal energy, stripping vulnerable dopaminergic cells of essential metabolic substrates, which in turn amplifies oxidative injury and hastens cellular demise [182, 183]. In response to this energetic catastrophe, current therapeutic explorations are pivoting toward supplying the brain with exogenous or alternative fuel sources. Direct lactate administration is being investigated to replenish the depleted substrate, whereas ketogenic regimens operate by redirecting whole-body metabolism toward the generation of ketone bodies, such as β-hydroxybutyrate. These metabolites readily traverse the blood-brain barrier and present a viable substitute for lactate in sustaining neuronal bioenergetics [184, 185]. The indispensability of astrocytic metabolic support is powerfully validated by empirical research, which reveals that, in cultured models, the availability of astrocytic lactate is absolutely critical for neuronal resilience and for shielding cells from neurotoxic insults [186]. Consequently, the mounting evidence points to a progressive erosion of this metabolic crosstalk as more than just a secondary event; rather, it is increasingly acknowledged as a primary pathogenic driver directly intertwined with both the onset and the relentless advancement of PD.

#### Lipid Droplet Biology as a Nexus for Alpha-Synuclein Aggregation

Shifting focus from lactate shuttle disruptions, astrocytic lipid homeostasis now stands out as a major determinant in Parkinson’s disease progression. Under normal circumstances, these glial cells function as the brain’s primary fat depot. They absorb surplus fatty acids and cholesterol from neurons through apolipoprotein E (ApoE)-dependent shuttling, safely storing these molecules within cytoplasmic lipid droplets (LDs) for future utilization or breakdown [187, 188]. This neuron-to-astrocyte relay is vital for preserving neuronal integrity; otherwise, lipid overload within neurons leads to toxic effects, membrane instability, and impaired mitochondrial performance. Astrocytes orchestrate this process via ATP-binding cassette transporter A1 (ABCA1), which facilitates cholesterol loading onto ApoE particles. These particles are then taken up by neurons through low-density lipoprotein receptors, including LDLR and LRP1 [187–189].

Tragically, this protective system collapses entirely under Parkinsonian conditions. Autopsy examinations of the substantia nigra in PD patients reveal a striking reversal of the normal pattern: rather than accumulating lipids, the astrocytes in this region actually possess fewer LDs compared to healthy controls, whereas the dopaminergic neurons become engorged with them [189]. This indicates that astrocytes have lost their ability to sequester neurotoxic lipids, effectively leaving adjacent neurons vulnerable to overwhelming lipotoxic damage. Adding to the complexity, the high-risk ApoE4 isoform demonstrates reduced lipid affinity and diminished ABCA1-mediated lipidation in astrocytes, which severely undermines this critical intercellular lipid exchange [187, 190]. Moreover, recent findings suggest that LDs are not merely passive storage units but can actively catalyze α-synuclein aggregation. When astrocytic LD clearance, whether through lipophagy or enzymatic lipolysis, is compromised, these droplets morph from harmless lipid reservoirs into dangerous nucleating platforms that drive α-syn fibril formation and propagation [189, 191]. This sets off a vicious, self-perpetuating feedback loop: astrocytic lipid mishandling worsens neuronal lipotoxicity, which in turn encourages α-synuclein misfolding on LD surfaces, thereby exacerbating both protein clumping and metabolic deterioration. The molecular mechanism by which α-syn nucleates on LDs involves direct binding to anionic phospholipids, particularly phosphatidylserine and phosphatidic acid, which become exposed on the LD monolayer surface during metabolic stress [189, 191, 192]. α-Syn binds via its N-terminal amphipathic α-helical region (residues 1–95) with a dissociation constant (Kd) of approximately 100 nM for phosphatidylserine-containing membranes, while the hydrophobic NAC region (residues 61–95) drives oligomerization directly on the LD surface [189, 193]. LD surface proteins perilipin 2 (PLIN2) and PLIN3, upregulated in PD astrocytes, act as scaffolds that concentrate α-syn, lowering its critical fibrillation concentration [191, 194]. Conversely, the LD-associated chaperone Hsp70, which normally maintains α-syn solubility, is reduced in PD astrocytes [189, 195, 196]. Lipolysis of LD triglycerides generates free fatty acids, particularly arachidonic acid and oleic acid, which bind α-syn and promote β-sheet conformational transition [191, 196]. This nucleation mechanism explains why LDs with impaired lipophagy become pathological aggregation hubs [189, 191].

#### Astrocyte-Mediated Intercellular Mitochondrial Trafficking

Emerging evidence fundamentally recasts astrocytes, positioning them not as passive bystanders but as dynamic cellular donors that actively shuttle healthy mitochondria to neurons under duress [43, 124, 197]. This coordinated rescue operates via tunneling nanotubes (TNTs), actin-based cytoplasmic bridges that deliver mitochondrial packets via molecular motors, and extracellular vesicles, alongside gap junction channels (notably Cx43) that exchange ions and small metabolites [198–202]. Given neurons’ enormous ATP consumption for synaptic transmission and transport, donated mitochondria provide immediate bioenergetic relief, restore ATP production, buffer calcium, and neutralize reactive oxygen species, stabilizing vulnerable cells against apoptotic stress [203–205].

In the context of PD, the disruption of this astrocytic support network exposes the fatal consequences of failed mitochondrial donation [206]. Misfolded α-syn and chronic inflammation impair TNT formation and Cx43 function, depriving substantia nigra neurons of astrocytic aid and driving their progressive degeneration into characteristic motor symptoms [207, 208]. This mechanistic insight has spurred neuroprotective strategies focused on augmenting intrinsic glial repair, boosting TNT production, enhancing Cx43 expression, or directly transplanting exogenous mitochondria [123, 209–211]. Co-culture studies validate these approaches, consistently showing that stressed neurons recover ATP, synaptic activity, and viability when co-cultured with competent astrocytes [212]. Consequently, astrocyte-mediated mitochondrial transfer marks a definitive paradigm shift, establishing glia as central therapeutic targets for halting neurodegenerative progression.

### Astrocyte-Endothelial Interactions and Blood-Brain Barrier Compromise

The blood-brain barrier (BBB) functions as the brain’s essential protective shield, a highly selective interface formed by endothelial cells sealed together with tight junction proteins such as claudin-5, which creates the primary physical seal, and ZO-1 (Zonula Occludens-1), which provides the critical structural scaffolding [213, 214]. The integrity of this barrier is not autonomously maintained by the endothelial cells alone but is heavily reliant on its dynamic partnership with astrocytes [215]. In a healthy state, these astrocytic end-feet provide trophic support and help regulate BBB function; however, in the pathology of PD, this supportive role is catastrophically reversed [9, 215]. Triggered by various insults, astrocytes become reactive and initiate a self-perpetuating cycle of disruption. They begin to overproduce and release potent lytic enzymes, primarily matrix metalloproteinases (MMPs) like MMP-9, which actively degrade and cleave the claudin-5 and ZO-1 proteins, effectively dismantling the tight junction complex [216].

Concurrently, reactive astrocytes release vascular endothelial growth factor (VEGF), a molecule that, despite its role in angiogenesis, significantly increases vascular permeability in the mature brain, further disrupting the organization of the remaining junctional proteins [217]. The collective impact of MMP-9 and VEGF is a profound structural compromise of the BBB, transforming it from a tightly regulated border into a leaky sieve [216, 217]. This breach has two devastating consequences: first, it permits neurotoxins from the systemic circulation, such as specific pesticides linked to PD risk, such as rotenone, paraquat or inflammatory molecules like bacterial lipopolysaccharide (LPS), to freely enter the brain parenchyma, where they can induce oxidative stress and directly damage vulnerable dopamine neurons in the substantia nigra [218–220]. Second, the compromised barrier allows for the unregulated infiltration of peripheral immune cells, such as T-cells and monocytes, into the brain [221]. Once inside, these cells become activated, releasing pro-inflammatory cytokines that fuel a state of chronic neuroinflammation, creating a toxic milieu that accelerates neuronal death and disease progression [222–224]. The therapeutic promise of this mechanism lies in its targetability; strategies using MMP inhibitors could halt the initial degradation of tight junctions, while agents that promote vascular stability, such as angiopoietin-1, can actively counteract VEGF and reinforce the barrier’s integrity [225]. The clinical relevance of this pathway is strongly supported by evidence showing a direct correlation between elevated levels of astrocyte-derived VEGF and the degree of BBB leakage observed in PD patients, solidifying this astrocyte-BBB axis as a promising frontier for developing disease-modifying therapies [226].

### System-Level Interfaces: Microbiota-Gut-Astrocyte Axis And Circadian Disruption

#### The Microbiota-Gut-Astrocyte (MGA) Axis

The traditional gut-brain axis model emphasizes neuronal (vagal) and immune (microglial) pathways [227]. A paradigm shift is underway with the recognition of a distinct microbiota-gut-astrocyte (MGA) axis, positioning astrocytes, central orchestrators of CNS homeostasis, metabolism, and inflammation, as direct targets of gut-derived microbial metabolites [228]. This axis functions via circulatory delivery of bioactive compounds that cross the BBB to engage specific receptors on astrocytes, modulating their phenotype and function. In PD, characteristic gut dysbiosis disrupts this signaling, depriving astrocytes of crucial homeostatic inputs while potentially exposing them to pro-inflammatory triggers [229–231]. This communication breakdown contributes to a persistent neuroinflammatory state and the failure of astrocytic support, exacerbating dopaminergic neurodegeneration.

The therapeutic promise of the MGA axis lies in its bidirectional modifiability. Preclinical data demonstrate that oral administration of sodium butyrate, a prototypical SCFA, rescues astrocytic metabolic dysfunction and reduces A1 polarization in MPTP models by activating FFAR2/3 receptors on astrocyte membranes [232]. Similarly, probiotic reconstitution with *Faecalibacterium prausnitzii*, a keystone SCFA producer depleted in PD patients, restores systemic butyrate levels and attenuates neuroinflammation in 6-OHDA-lesioned rats [233]. Critically, fecal microbiota transplantation (FMT) from healthy donors to PD patients in pilot trials has shown preliminary motor improvement (UPDRS-III reduction), mechanistically linked to restored circulating SCFAs and reduced serum LPS [234, 235]. These findings position the MGA axis as a tractable target for systemic metabolic reprogramming of astrocytes, circumventing blood-brain barrier challenges inherent to direct CNS drug delivery.

The primary mediators of the MGA axis are microbial metabolites, whose production and systemic levels are directly determined by the composition and functional capacity of the gut microbiota. Short-chain fatty acids (SCFAs**)** such as butyrate, propionate, and acetate, produced by fermentative bacteria, are potent histone deacetylase inhibitors and ligands for free fatty acid receptors (FFAR2/3) on astrocytes [236]. This signaling promotes astrocyte metabolic quiescence, enhances barrier functions of the BBB, and stimulates the release of neurotrophic factors. PD dysbiosis, often deficient in SCFA-producers like *Faecalibacterium*, results in diminished tonic signaling, contributing to astrocyte dysfunction and a pro-inflammatory shift [236]. Microbial conversion of dietary tryptophan yields aryl hydrocarbon receptor (AhR) agonists, such as indole-3-propionic acid [237]. AhR activation in astrocytes is a key regulator of the anti-inflammatory transcriptional program, suppressing NF-κB signaling and reducing the production of inflammatory mediators like C3. A deficit in these metabolites, common in PD, leaves astrocytes susceptible to inflammatory polarization [237, 238].

However, pro-inflammatory microbe-associated molecular patterns (MAMPs), in the context of heightened intestinal permeability, allow bacterial components such as LPS and peptidoglycans to enter systemic circulation and the brain [239, 240]. These chemicals stimulate astrocytic TLRs, especially TLR4, propelling them into a harmful reactive A1-like state marked by the secretion of cytokines and complement components that are neurotoxic. Dysbiosis can modify the transformation of the main bile acids into secondary bile acids. Although certain secondary bile acids, including tauroursodeoxycholic acid, exhibit neuroprotective properties, an unbalanced profile may lead to oxidative stress and inflammation [241].

So, therapeutic strategies targeting the MGA axis aim to restore the beneficial signaling balance. By correcting the peripheral metabolic signals that govern astrocyte health, such interventions offer a promising, indirect route to modulate the neuroinflammatory landscape in PD (Table 2).

**Table 2:** Microbial metabolites modulating the microbiota-gut-astrocyte axis: molecular mechanisms and therapeutic implications

Metabolite class	Primary source/example	Receptor/target on astrocyte	Normal homeostatic function	Consequence of PD-associated dysbiosis	Potential therapeutic intervention	References
Short-chain fatty acids (SCFAs)	Fermentation of fiber by bacteria such as *Faecalibacterium prausnitzii*. Butyrate, Propionate	FFAR2/3 (GPR43/41);HDAC inhibition	Promotes anti-inflammatory phenotype; supports BBB integrity; stimulates neurotrophic factor release (GDNF)	Reduced signaling. Leads to loss of metabolic support, increased inflammatory susceptibility, and impaired barrier function	Prebiotics (dietary fiber), SCFA prodrugs, and specific probiotic supplementation	[242–245]
Tryptophan metabolites (AhR ligands)	Microbial metabolism of tryptophan (e.g., Indole-3-propionic acid, Indole-3-aldehyde)	Aryl Hydrocarbon Receptor (AhR)	Drives anti-inflammatory gene expression; suppresses astrocytic NF-κB pathway; enhances antioxidant defenses	Agonist deficiency. Results in unchecked neuroinflammation and loss of cytoprotective responses in astrocytes	Tryptophan-rich diet; probiotics that produce indoles; AhR agonist compounds	[237, 246]
Secondary bile acids	Microbial dehydroxylation of primary bile acids (e.g., deoxycholic acid, tauroursodeoxycholic acid (TUDCA))	TGR5, Farnesoid X receptor (FXR)	Modulates energy metabolism and inflammation; TUDCA has potent anti-apoptotic and ER-stress reducing effects	Altered profile. May shift balance towards more toxic, inflammatory species and reduce protective signaling	Bile acid sequestrants; administration of neuroprotective bile acids like TUDCA	[247]
Pro-inflammatory MAMPs	Gram-negative bacteria (LPS); Gram-positive bacteria (Peptidoglycan)	Toll-like Receptor 4 (TLR4), TLR2	Low-level surveillance for infection; part of innate immune priming	Increased systemic translocation due to gut leakiness. Drives astrocyte reactivity (A1 polarization), leading to chronic neuroinflammation and complement-mediated neuronal damage	Strategies to improve gut barrier integrity (e.g., glutamine, zinc); anti-TLR4 therapeutics; reduce bacterial overgrowth	[248, 249]

#### Circadian Rhythm Disruption and Suprachiasmatic Nucleus Pathology

The disruption of circadian rhythms in PD represents a complex pathology where astrocytes transition from supportive glial cells to active contributors of neuronal dysfunction, profoundly impacting the brain’s central timekeeper, the suprachiasmatic nucleus (SCN) [250, 251]. In the healthy brain, astrocytes are indispensable for circadian function, regulating the extracellular balance of ions and neurotransmitters like glutamate and GABA, and providing metabolic fuel to SCN neurons in the form of lactate [153, 252, 253]. However, in the PD brain, these cells undergo a pathological transformation, becoming reactive and initiating a cascade of disruptive events. This reactive astrogliosis is characterized by the sustained release of pro-inflammatory cytokines, such as TNF-α and IL-6, which directly impair the electrical firing and molecular clockwork of SCN neurons by interfering with core clock genes like Bmal1 and Per2 [254, 255]. Concurrently, astrocytic homeostasis is critically compromised; they lose their ability to efficiently clear synaptic glutamate, leading to excitotoxicity, and their regulation of GABA is altered, destabilizing the delicate balance of neuronal excitation and inhibition necessary for robust circadian rhythmicity [255, 256]. Furthermore, the astrocyte-neuron lactate shuttle, a key energy-delivery system, is impaired, leaving SCN neurons energetically deficient and unable to sustain the high metabolic demands of circadian timekeeping [257]. Pathological α-syn, which some studies suggest can directly propagate to and corrupt astrocyte function, often exacerbates this multifaceted astrocytic failure [258]. Consequently, the destabilization of the SCN’s master clock by these dysfunctional astrocytes not only manifests as the classic non-motor symptoms of PD, such as fragmented sleep, excessive daytime sleepiness, and sundowning, but also may create a vicious cycle where a disrupted circadian system fails to provide its neuroprotective benefits, potentially accelerating the broader neurodegenerative process [251, 256]. Therefore, astrocytes in PD are not passive bystanders but central actors in dismantling the temporal architecture of the brain, linking core pathology directly to the debilitating circadian rhythm disturbances experienced by patients. The specific mechanisms by which astrocytes disrupt SCN function are now understood at the molecular level [2, 252, 259]. First, reactive astrocytes in PD release TNF-α and IL-1β, which suppress core clock gene expression (Bmal1 and Per2) via NF-κB-mediated HDAC1 recruitment to their promoters, reducing transcriptional amplitude in SCN slice cultures [254, 255, 260–263]. Second, impaired astrocytic glutamate homeostasis via reduction in EAAT2 expression in PD SCN causes glutamate spillover and tonic extrasynaptic NMDA receptor activation, dampening rhythmic firing, while increased astrocytic GABA release through Bestrophin‑1 channels activates presynaptic GABAB receptors on retinohypothalamic terminals, blocking light‑induced phase resetting [253, 255, 256, 264]. Third, failure of the astrocyte‑neuron lactate shuttle via reduction in MCT4 expression deprives SCN neurons of metabolic fuel, causing ATP deficit, circadian period lengthening, and amplitude reduction [257, 265, 266]. Fourth, pathological α‑syn oligomers internalized by SCN astrocytes bind and sequester BMAL1 in the cytoplasm, reducing clock‑controlled gene expression and creating a cell‑autonomous feedback loop that destabilizes SCN neuronal synchrony [21, 168, 251, 267].

In conclusion, astrocytes are central players in the multifaceted pathology of PD. Their dysfunction spans a wide spectrum of activities, from immune regulation and metabolic support to protein clearance and intercellular communication. The therapeutic strategies emerging from this understanding are increasingly cell-type-specific, aiming not to abolish astrocyte function but to restore their homeostatic and protective capacities. Future research and clinical trials will be essential to translate these mechanism-based insights into effective treatments that can modify the progression of PD by targeting these pivotal glial cells.

### Temporal Hierarchy of Astrocytic Failure: Primary Drivers vs. Secondary Consequences

The previous sections outline the complex structure of astrocytic dysfunction in PD, although a crucial unresolved question pertains to the temporal order of these pathogenic events: which mechanisms serve as key drivers and which are subsequent consequences [17, 268]? We propose a hierarchical model delineating three temporal periods based on information from longitudinal animal models and human postmortem staging investigations.

Primary mechanisms include glymphatic failure resulting from AQP4 depolarization and compromised α-synuclein clearance through the autophagy-lysosomal pathway, which manifest prior to significant neuronal death [95, 96]. AQP4 mislocalization is observable in A53T α-synuclein transgenic mice as early as 2 months of life, preceding motor impairments or dopaminergic cell death [100]. Lysosomal failure in astrocytes with GBA1 mutations constitutes a fundamental genetic susceptibility that disrupts proteostasis from the beginning [73]. These fundamental deficiencies foster a conducive environment for α-synuclein aggregation and dissemination.

Secondary amplifying mechanisms are neuroinflammation (via conversion to reactive astrocytes), metabolic dysfunction (disruption of the lactate shuttle), and glutamate excitotoxicity, which generally arise subsequent to prolonged protein buildup [11, 76, 154, 183]. In PD models, reactive astrocyte phenotypes manifest only after extracellular α-synuclein attains critical thresholds, indicating that these are responses to pathology rather than its initiators [26, 27]. The activation of the NLRP3 inflammasome in astrocytes, albeit harmful, necessitates priming by signals generated from microglia, which are contingent upon preceding neuronal stress [31, 32].

**Fig. 4:**
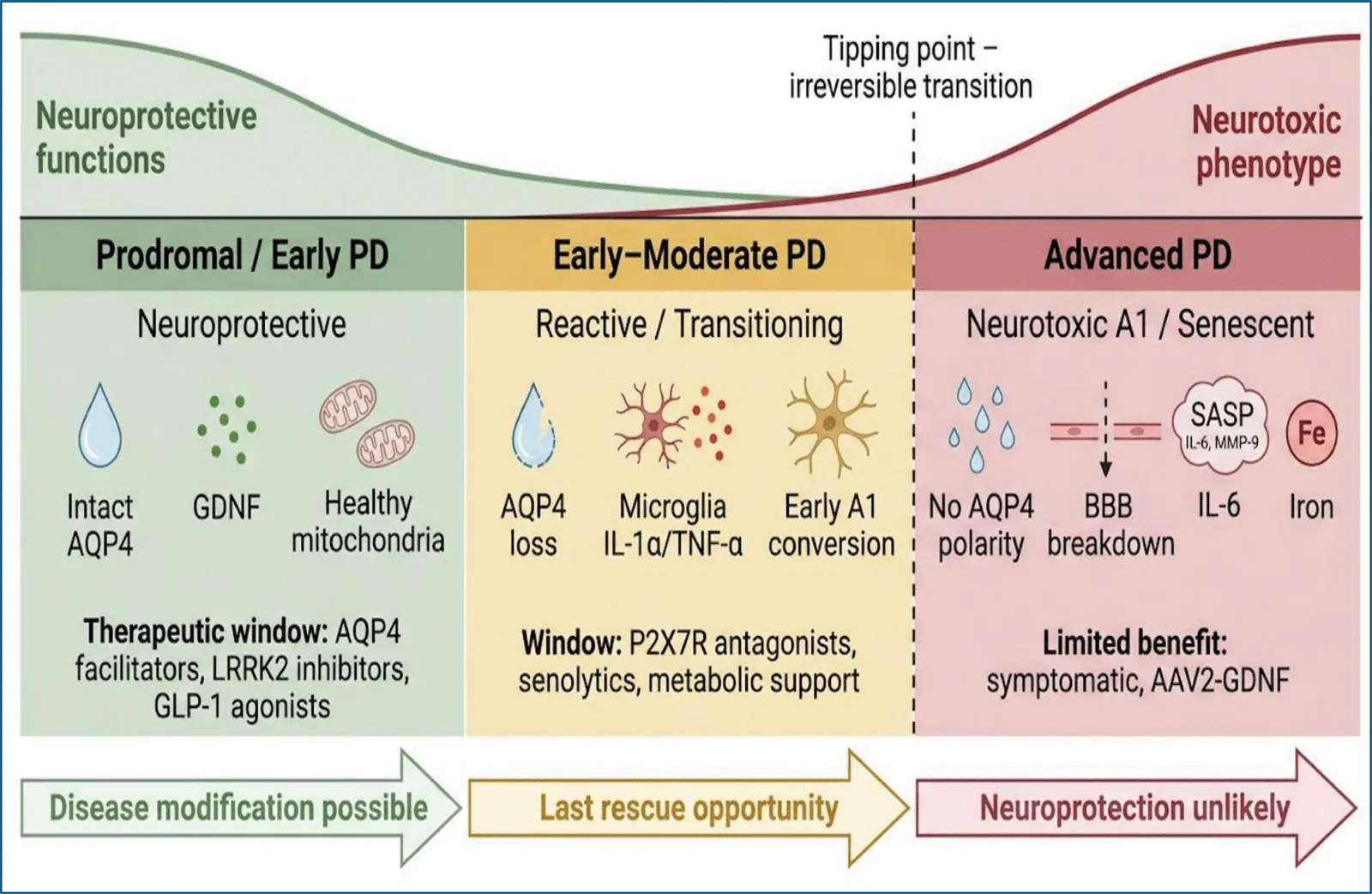
Progression of Parkinson’s disease and associated therapeutic windows. This schematic outlines the irreversible progression of Parkinson’s disease across three stages, separated by a critical tipping point. The prodromal/early stage (green) features intact neuroprotection (AQP4, GDNF, mitochondria), offering a window for disease modification. The early-moderate stage (yellow) is a reactive transition (AQP4 loss, microglial activation) representing the last rescue opportunity. The advanced stage (red) exhibits a neurotoxic, senescent phenotype (BBB breakdown, IL-6, iron), where neuroprotection is unlikely and only symptomatic interventions remain

Tertiary late/decompensatory mechanisms, senescence as a SASP phenotype, iron dysregulation with susceptibility to ferroptosis, and collapse of the BBB generally appear in advanced disease, coinciding with extensive neurodegeneration [15, 269, 270]. These probably indicate terminal astrocytic failure, in which homeostatic reserves are depleted (Fig. 4).

## Translational Perspectives: Astrocyte-Directed Therapeutic Strategies and Clinical Implementation

Recent advances have positioned astrocytes as central agents in Parkinson’s pathology, fundamentally reshaping the drug discovery landscape [26, 271]. In contrast to conventional symptomatic approaches that simply replenish dopamine, emerging astrocyte-directed interventions are designed to re-establish cerebral equilibrium, temper neuroinflammatory responses, and arrest ongoing neuronal loss [2, 272]. Assessed here are the leading pharmacological candidates currently navigating preclinical development and clinical translation, with a particular focus on mechanisms that reinforce the intrinsic neuroprotective functions of these glial cells. A range of ongoing clinical investigations, whether primarily designed or secondarily aimed at rectifying astrocyte malfunction in PD, are actively being pursued.

Among these, GLP-1 receptor agonists stand at the vanguard, with lixisenatide demonstrating notable promise as a disease-modifying agent. Concurrently, pharmacological blockade of LRRK2 is being explored to counter both lysosomal impairment and glymphatic clearance deficits within astrocytes, as detailed in Table 3.

**Table 3:** Clinical-stage investigational agents targeting astrocyte-mediated pathways in Parkinson’s disease

Mechanistic strategy arm	Investigational agent (class)	Precision astrocytic reprogramming strategy	Current clinical milestone and key outcome data	References
I. inflammatory and metabolic rescue	Lixisenatide (GLP-1RA)	Counteracts microglia-driven A1 astrocytic polarization; safeguards astrocytic-neuronal lactate shuttling (ANLS) to preserve metabolic coupling	Phase II (Completed): *LixiPark* (NCT03439943)—achieved primary endpoint; yielded a significant 3.0-point reduction in MDS-UPDRS III motor decline over 12 months vs. placebo (*p* = 0.0068)	[273, 274]
	Semaglutide (oral) (GLP-1RA)	Exerts broad neuroimmunomodulation by suppressing NLRP3 inflammasome assembly and curtailing astrocyte-derived IL-6 secretory cascades	Phase III (Active–AD trial): *EVOKE* + (NCT04777409)—although designed for Alzheimer’s, its anti-gliotic properties are highly translatable to PD; primary completion forecast for 2026	[275, 276]
	NLY01 (pegylated GLP-1RA)	Engineered as a long-acting variant to preemptively block the phenotypic conversion of quiescent astrocytes into neurotoxic A1-reactive glia	Phase II (completed): NCT04154072—did not meet the primary efficacy bar across the general PD population; however, exploratory subgroup analyses revealed a potential signal in early-onset patients (< 60 years)	[277]
II. Glymphatic and lysosomal homeostasis	BIIB122/DNL151 (LRRK2 Inhibitor)	Restores physiological aquaporin-4 (AQP4) surface polarization at astrocytic perivascular end-feet, concurrently normalizing endolysosomal clearance pathways	Phase IIb (active): *LUMA* (NCT05348785)—actively recruiting ~ 640 early-stage patients (sporadic + LRRK2 carriers) to assess combined efficacy endpoints and validated fluid biomarkers	[278]
	TGN-073 (AQP4 Facilitator)	Pharmacologically enhances convective fluid fluxes via AQP4, boosting perivascular CSF-interstitial solute clearance without altering total AQP4 expression levels	Preclinical: in vivo rodent studies demonstrate significant augmentation of interstitial fluid circulation and glymphatic efflux of pathological solutes	[279, 280]
III. Senolytic glial remodeling	Dasatinib + quercetin (D + Q) (senolytic cocktail)	Selectively ablates senescent astrocytic populations; eliminates the senescence-associated secretory phenotype (SASP), thereby reducing chronic IL-6, IL-1β, and MMP neuroinflammation	Phase I/II (active–AD trial): *ALSenLite* (NCT04063124)—currently under investigation for Alzheimer’s; the clearance of “zombie glia” offers a directly applicable therapeutic avenue for PD-related glial senescence	[281]
IV. Trophic biofactory engineering	AAV2-GDNF (AB-1005) (gene therapy)	Utilizes MRI-guided convection-enhanced delivery (CED) to transduce striatal astrocytes, effectively transforming them into sustained, localized GDNF-secreting bioreactors	Phase Ib (completed): NCT04167540—confirmed a strong safety profile; treated subjects exhibited stable motor functionality alongside increased putaminal dopamine transporter (DAT) binding at the 18-month follow-up	[282]

### Stage-Specific Therapeutic Targeting: Matching Mechanism to Disease Phase

The temporal hierarchy of astrocytic dysfunction outlined above has profound implications for clinical trial design and patient stratification, as interventions must be matched to the dominant pathological process at each disease stage to achieve efficacy [2]. In the prodromal/early stage (approximately 5–10 years before motor diagnosis) [283], astrocytes retain significant compensatory capacity, making this the optimal window for AQP4 facilitators like TGN-073 to enhance glymphatic clearance [279, 280, 284, 285], LRRK2 inhibitors to prevent AQP4 phosphorylation and maintain end-foot polarity [99, 286], and GLP-1 receptor agonists to block microglia-mediated A1 conversion before it begins [287, 288], though trials in this window require prodromal biomarkers such as RBD, hyposmia, or DAT imaging [289, 290]. During early-moderate PD (H&Y stages 1–2.5), as astrocytes begin transitioning to reactive phenotypes, the most appropriate interventions include agents targeting the A1 conversion process (NLY01 and P2X7R antagonists), metabolic support (lactate and ketogenic diets), and early senolytics (dasatinib + quercetin) to clear nascent senescent astrocytes before their SASP amplifies inflammation; this window likely represents the last opportunity for true disease modification before irreversible neuronal loss [41, 54, 277, 287, 291–293]. In advanced PD (H&Y stages 3–5), where astrocytes have fully adopted a neurotoxic A1 phenotype with established SASP and BBB breakdown [294, 295], neuroprotective strategies face diminished returns, and the therapeutic focus should shift to symptomatic management [296], though gene therapies (AAV2-GDNF) and mitochondrial transfer strategies may still provide trophic support to remaining neurons [297, 298]. Clinical trials that fail to stratify patients by disease stage risk erroneously conclude the inefficacy of otherwise promising agents [299, 300].

### GLP-1 Receptor Agonists and Glial Crosstalk Modulation

Among the most promising strategies currently under investigation for Parkinson’s disease are the glucagon-like peptide-1 (GLP-1) receptor agonists, a class that has already yielded several candidates with demonstrable disease-modifying activity in clinical settings [301, 302]. Initially developed to manage type 2 diabetes, these compounds confer neuroprotection through two primary mechanisms: modulating the crosstalk between microglia and astrocytes and re-establishing metabolic equilibrium [287, 301].

The path from bench to bedside, however, has been anything but uniform, as underscored by the contrasting fates of various GLP-1 analogs in recent trials. The early preclinical promise surrounding NLY01 did not translate into success in its Phase 2 trial, which fell short of its primary goal of curbing motor decline in early-stage PD; nevertheless, subsequent post hoc evaluations did hint at a benefit for patients under 60 years old [277]. On the other hand, the GLP-1 agonist lixisenatide hit a significant milestone in the 2024 LixiPark Phase 2 trial, where it produced a marked attenuation of motor disability progression, measured via the MDS-UPDRS Part III, relative to placebo over a 1-year period in early PD patients [273]. This success reinforces the viability of this therapeutic class, contingent upon the agent’s capacity to penetrate the blood-brain barrier effectively. The recent failure of semaglutide to achieve its primary cognitive endpoint in the Phase 3 EVOKE + trial for Alzheimer’s disease (NCT04777409) has prompted some skepticism regarding its utility in chronic neurodegeneration. However, given that the AD study focused on late-stage amyloid pathology, this outcome does not necessarily undermine its relevance to PD, especially since GLP-1R-mediated suppression of microglial IL-1α/TNF-α/C1q release—which prevents the harmful conversion of astrocytes to the A1 phenotype—remains a highly PD-relevant pathway. The lixisenatide results underscore that agent-specific pharmacokinetic properties, including BBB penetrance, receptor occupancy duration, and the timing of intervention, are paramount to therapeutic success. Accordingly, a dedicated direct assessment of semaglutide in early-stage PD, rather than relying on inferences drawn from AD trials, is clearly justified [303].

Building on this monoagonist foundation, next-generation compounds that combine GLP-1 with GIP receptor activation, exemplified by DA5-CH, have demonstrated markedly superior outcomes in animal models [292, 304]. This dual agonist not only traverses the BBB more efficiently but also exerts a more potent effect, curbing astrocyte hyperreactivity, diminishing α-synuclein accumulation, and tempering NF-κB-driven neuroinflammatory cascades. Collectively, these preclinical observations indicate that dual incretin receptor agonists are poised to spearhead the forthcoming generation of glial-centric therapeutic interventions. Table 4 compares the mechanisms, preclinical evidence, and clinical trial outcomes of GLP-1 receptor agonists and dual incretin analogs targeting astrocyte-mediated pathways in PD.

**Table 4:** Comparative analysis of GLP-1 receptor agonists and glial crosstalk modulators

Agent	Primary astrocytic/glial target	Mechanism of action	Key preclinical evidence	Clinical trial status	Outcome/findings	References
Lixisenatide	Astrocyte-neuron metabolic coupling; microglia-astrocyte crosstalk	GLP-1R agonism; counteracts microglia-driven A1 astrocytic polarization; safeguards astrocyte-neuron lactate shuttle (ANLS)	Reduced astrocyte reactivity and preserved metabolic support in toxin models	Phase II (Completed): LixiPark (NCT03439943)	Achieved primary endpoint; significant 3.0-point reduction in MDS-UPDRS III motor decline over 12 months vs. placebo (*p* = 0.0068)	[273, 274]
NLY01 (pegylated GLP-1RA)	Prevents A1 astrocyte conversion	Engineered long-acting variant that preemptively blocks phenotypic conversion of quiescent astrocytes into neurotoxic A1-reactive glia via microglial IL-1α/TNF-α/C1q suppression	Blocked A1 astrocyte conversion and protected dopaminergic neurons in MPTP models	Phase II (completed): NCT04154072	Did not meet primary efficacy bar across general PD population; exploratory subgroup analysis showed potential signal in early-onset patients (< 60 years)	[277, 287]
Semaglutide (oral)	NLRP3 inflammasome; astrocyte-derived IL-6 secretion	Broad neuroimmunomodulation; suppresses NLRP3 inflammasome assembly and curtails astrocyte-derived IL-6 secretory cascades	Anti-gliotic properties demonstrated in preclinical models	Phase III (active): EVOKE + (NCT04777409)—Alzheimer’s disease trial	Designed for Alzheimer’s; anti-gliotic properties highly translatable to PD; primary completion forecast for 2026	[276, 305]
DA5-CH (dual GLP-1/GIP agonist)	Astrocyte hyperreactivity; NF-κB-driven inflammation	Dual incretin receptor activation; superior BBB penetration; curbs astrocyte hyperreactivity, diminishes α-synuclein accumulation, tempers NF-κB-driven neuroinflammatory cascades	Superior to single GLP-1R agonists in MPTP mouse model; reduced astrocyte activation and neuroinflammation	Preclinical	Demonstrated markedly superior outcomes vs. single GLP-1 agonists in reducing astrocyte hyperreactivity and neuroinflammation	[292, 304]
Exenatide	Neuroinflammatory modulation	GLP-1R agonism; reduces microglial activation and astrocyte inflammatory responses	Neuroprotective in animal models; reduces α-synuclein toxicity	Phase II (completed): multiple trials	Mixed results; some evidence of neuroprotection but inconsistent outcomes across studies	[301, 302, 306, 307]
Liraglutide	Metabolic and inflammatory pathways	GLP-1R agonism; enhances mitochondrial function and reduces oxidative stress in glial cells	Protected against MPTP-induced dopaminergic loss; reduced astrogliosis	Phase II (Completed)	Demonstrated neuroprotective potential; further studies warranted	[308–311]

### Clearing Zombie Glia: Senolytic Therapies

Under the sustained duress of iron dysregulation and oxidative damage characteristic of PD, certain astrocytes transition into a senescent phenotype. Although these “zombie cells” arrest their cell cycle, they persist as metabolic powerhouses, releasing a hazardous senescence-associated secretory phenotype (SASP). This cocktail, featuring IL-6, IL-1β, and various matrix metalloproteinases (MMPs), actively breaks down the extracellular milieu and instigates neuronal demise [269, 270].

Targeting these zombie cells directly, senolytics represent a pharmacological breakthrough that executes a “hit-and-run” tactic by specifically triggering apoptotic pathways [312]. Preclinical evidence, particularly the dasatinib and quercetin (D + Q) duo, demonstrates effective clearance of senescent glial cells and mitigation of neuroinflammatory responses in tauopathy models [313]. Although initial clinical investigations, such as ALSENLITE (NCT04063124), are currently focused on AD, the pronounced parallels in astrocyte senescence mechanisms strongly position this approach as a highly promising avenue for PD [281]. Rather than simply suppressing inflammatory cascades, this intervention physically purges the chronic inflammatory source.

Consequently, senolytics may effectively arrest the detrimental bystander effect, a phenomenon wherein senescence-associated toxins are transmitted from compromised astrocytes to their otherwise healthy counterparts.

### Pharmacological Restoration of Glymphatic Clearance via Aquaporin-4 Modulation

Central to PD pathology is a severe impairment of the glymphatic system, wherein AQP4 depolarization directly obstructs the clearance of soluble α-synuclein aggregates [314, 315]. Through direct phosphorylation of AQP4, LRRK2 mutations drive the protein away from astrocytic end-feet, thereby hampering glymphatic inflow and simultaneously intensifying neuroinflammatory responses due to defective IFNγ removal [99]. In genetic models of the disease, inhibiting LRRK2 pharmacologically has been shown to reinstate AQP4 polarity, boost glymphatic drainage, and dampen IFNγ-driven neuroinflammation [99]. Currently, LRRK2 inhibitors are being evaluated in clinical trials specifically targeting carriers of LRRK2 mutations [316]. Moreover, the enzymatic cleavage of β-dystroglycan by MMP-9 undermines the stable anchoring of AQP4 to the vascular basement membrane; by preventing this degradation, MMP-9 inhibition preserves AQP4 polarization and glymphatic activity, as demonstrated in MPTP models [101].

Turning to methods that do not rely on drugs, therapeutic promise is also evident in strategies such as enhancing non-REM slow-wave sleep, the prime physiological condition for glymphatic function, via orexin antagonists, or sodium oxybate to boost protein removal [317–320]. Similarly, very low-intensity ultrasound (VLIUS) has been observed to stimulate glymphatic inflow through the astrocytic TRPV4-AQP4 axis, thereby promoting waste clearance while avoiding tissue injury [321]. More recently, TGN-073, a new AQP4 facilitator, has demonstrated efficacy in boosting interstitial fluid movement and glymphatic clearance independently of changes in AQP4 expression, thus providing a direct drug-based avenue for reinstating cerebral waste elimination [279, 280].

Collectively, these pharmacological and non‑pharmacological strategies converge on a unified goal: restoring AQP4‑mediated glymphatic clearance to halt the feed‑forward cycle of α‑synuclein accumulation and neurodegeneration. Table 5 synthesizes these emerging interventions to guide future therapeutic development.

**Table 5:** Pharmacological and non-pharmacological strategies for restoring glymphatic function via aquaporin-4 modulation

Strategy category	Intervention/target	Mechanism of action	Key supporting evidence	Development stage	References
I. Pharmacological interventions					
AQP4 small-molecule modulators	TGN-073 (AQP4 facilitator)	Enhances AQP4-mediated water flux, promoting convective CSF–ISF exchange and glymphatic clearance	In vivo: TGN-073 increases glymphatic transport by up to 41% in rat brain; Gd-DTPA distribution broadens, water diffusivity rises	Preclinical (rodent)	[279, 280]
LRRK2-targeted strategies	LRRK2 kinase inhibitors (e.g., BIIB122/DNL151)	Prevent LRRK2-mediated AQP4 phosphorylation, restore AQP4 polarization at astrocytic end-feet, and rescue IFNγ clearance	LRRK2 R1441G directly phosphorylates AQP4, causing depolarization; pharmacological inhibition restores polarity, improves glymphatic function, reduces IFNγ-driven neuroinflammation, and protects dopamine neurons	Phase IIb (LUMA trial, NCT05348785)	[99, 322]
MMP-9 inhibition	GM6001 (MMP-9 antagonist)	Blocks MMP-9-mediated cleavage of β-dystroglycan, preserving the anchoring of AQP4 to the vascular basement membrane	In MPTP and A53T models, MMP-9 and cleaved β-DG are upregulated; MMP-9 inhibition restores AQP4 polarization, ameliorates glymphatic dysfunction, and reduces neuronal loss	Preclinical (mouse)	[25, 323]
Circadian-clock modulation	Agomelatine (melatonergic antidepressant)	Enhances AQP4 polarization via BMAL1-dependent transcriptional regulation, repairing the circadian-glymphatic axis	In MPTP-PD mice, agomelatine restores AQP4 polarity, improves motor performance, and reduces α-syn pathology; first compound targeting the clock–glymphatic–AQP4 axis	Preclinical (repurposed approved drug)	[324]
Traditional Chinese medicine	Tianqi Pingchan Granule (TPG)	Restores AQP4 polarization and preserves glymphatic integrity	In L-DOPA-induced dyskinesia (LID) rat models, TPG increases fluorescent tracer influx, restores AQP4 localisation and expression, reduces Aβ deposition and astrogliosis	Preclinical (herbal formulation)	[325]
II. Non-pharmacological interventions					
Sleep optimisation	Sleep enhancement/sleep disorder treatment	Sleep is the primary physiological driver of glymphatic flow; reduces astrocytic LAG3 upregulation and restores AQP4 polarity	Sleep deprivation upregulates astrocytic LAG3, disrupts AQP4 polarization, impairs CSF inflow and α-syn clearance; LAG3 knockdown restores AQP4 polarity and glymphatic function	Lifestyle intervention	[326]
Sensory stimulation	40-Hz light flicker	Enhances astrocytic AQP4 polarization and vasomotion via adenosine A2A receptor signalling, promoting glymphatic inflow/outflow	40-Hz flicker frequency-dependently increases glymphatic influx and efflux in awake mice, independent of anaesthesia or sleep	Preclinical (mouse)	[327]
Physical exercise	High-intensity interval training (HIIT)	Promotes AQP4 polarization and augments glymphatic protein clearance	HIIT enhances AQP4 polarization and facilitates Aβ clearance via the glymphatic system	Preclinical/lifestyle intervention	[328, 329]
Lymphatic/gamma stimulation	Cervical lymphatic stimulation; gamma-rhythm stimulation	Enhances meningeal lymphatic drainage and glymphatic inflow	Emerging non-invasive strategies showing restoration of glymphatic function in AD and PD models	Preclinical/early exploration	[330]

### Gene Therapeutic Engineering of Astrocytes as Trophic Biofactories

Leveraging the inherent longevity and homeostatic capabilities of astrocytes within the CNS, a transformative neuroprotective strategy for Parkinson’s disease involves engineering them into sustained, localized neurotrophic factor sources [331]. In contrast to direct neuronal transduction, this astrocytic approach creates stable biological factories that secrete therapeutic proteins into the extracellular milieu, offering sustained support to degenerating dopaminergic terminals while avoiding the excitotoxicity risks frequently tied to neuronal overexpression.

Optimized adeno-associated viral vectors (AAV2-GDNF) have been established as durable expression platforms capable of transducing striatal cells for long-term glial cell line-derived neurotrophic factor (GDNF) secretion [332, 333]. To overcome the poor diffusion profiles of conventional injections, modern clinical efforts, such as the Phase 1b AB-1005 trial, have adopted MRI-guided convection-enhanced delivery (CED) [282]. This technique achieves extensive and consistent putaminal coverage, which has resulted in stabilized motor function and heightened dopamine transporter binding over 18-month follow-up periods. By ensuring GDNF levels remain steady, this approach seeks to preserve the functional integrity of the nigrostriatal pathway and protect surviving dopaminergic neurons from further deterioration [334].

Moving past the strategy of trophic factor secretion, cutting-edge in vivo reprogramming techniques present a bolder therapeutic option: directly transforming reactive astrocytes into neuronal cells to restore disrupted circuitry [335, 336]. Utilizing CRISPR activation (CRISPRa), recent investigations have successfully converted striatal astrocytes into induced GABAergic neurons through targeted activation of the endogenous Ascl1, Lmx1a, and Nr4a2 loci [335, 337]. These induced neurons functionally assimilate into striatal networks and have been shown to mitigate voluntary motor deficits in 6-OHDA toxin-induced PD models [338]. This approach circumvents the necessity for exogenous cell grafting and suggests that reestablishing inhibitory GABAergic tone in the striatum could offset dopamine loss, thereby offering a novel therapeutic avenue independent of dopamine replacement.

Astrocytic utility extends prominently into cell replacement approaches, where co-transplanting their engineered counterparts has been found to markedly improve the survival of grafted neurons [339]. Specifically, ventral midbrain-derived astrocytes (VM-Ast) that are engineered to overexpress Nurr1 and FoxA2 enhance the maturation, synaptic integration, and resilience of co-grafted neural progenitor cells (NPCs) [340, 341]. Mechanistically, this synergistic benefit is driven by activation of the Nrf2/Wnt/β-catenin signaling cascade [339]. This pathway shifts the local microenvironment away from a pro-inflammatory, immunogenic state and toward a neuroprotective, antioxidant phenotype, consequently reducing graft-induced dyskinesias while fostering durable structural repair of the nigrostriatal pathway.

### Targeted Disruption of Astrocyte-Specific Inflammatory Pathways

Central to the self-perpetuating loop of sustained neuroinflammation is the P2X7 receptor (P2X7R), whose engagement of the NLRP3 inflammasome in both microglia and reactive astrocytes ignites the inflammatory cascade [342]. When dying neurons release extracellular ATP, this receptor is stimulated, prompting the continuous discharge of IL-1β and IL-18, which in turn exacerbates astrocyte reactivity and accelerates neuronal injury [343].

While preclinical evidence has established that the P2X7R antagonist Brilliant Blue G (BBG) curbs astrocytosis, dampens microglial activation, and safeguards dopaminergic neurons in 6‑OHDA models, its translational potential is severely constrained by poor BBB penetration and off‑target effects like skin discoloration [344, 345]. In response, research has pivoted toward developing next‑generation antagonists that cross the BBB effectively—among them JNJ‑47965567, CE‑224,535, and A‑438079, which offer markedly improved pharmacokinetics and stand as viable candidates for disrupting the astrocyte–microglia inflammasome axis in PD [346–348]. Consequently, disrupting the P2X7R-NLRP3 signaling axis has emerged as a leading strategy to break this feed-forward inflammatory loop.

Building on this, direct targeting of the inflammasome machinery itself, alongside its upstream transcriptional regulators, represents a parallel therapeutic axis to intercept neuroinflammation at its source. At the forefront of this process is the NLRP3 inflammasome, which drives IL-1β production and A1 astrocyte formation; selective antagonists like MCC950 have demonstrated efficacy in curtailing neuroinflammation in preclinical models [349, 350]. Concurrently, the NF-κB pathway serves as a transcriptional hub for numerous pro-inflammatory agents in reactive astrocytes, and targeted inhibitors that block astrocytic NF-κB activation manage to reduce inflammation while preserving broader systemic immune responses [351].

In the pursuit of robust neuroprotective functions, the use of PPARγ agonists stands out as a promising tactic. These compounds actively foster an anti-inflammatory astrocyte profile, effectively bolstering glutamate clearance and enhancing the production of endogenous antioxidants [352]. Equally critical to this regulatory web is the canonical Wnt/β-catenin signaling pathway, which forms a vital astrocyte-neuron communication axis that frequently falters in PD [164]. Under physiological conditions, astrocytes release Wnt1 to stabilize neuronal β-catenin, thereby safeguarding dopaminergic neurons; however, the oxidative milieu characteristic of PD suppresses this protective dialogue, increasing neuronal vulnerability [32, 353]. Pharmacological intervention targeting GSK-3β, a key suppressor of Wnt, offers a means to rejuvenate astrocytic Wnt signaling, thus restoring trophic support and curbing local inflammatory responses [32]. On the opposing side of this signaling landscape, the MAPK cascades, specifically the c-JNK pathway, exhibit heightened activity in PD astrocytes, stimulating the discharge of inflammatory alarmins and cytokines. Evidence indicates that preventing JNK activation within these glial cells effectively halts the secretion of TNF-α and nitric oxide, thereby successfully attenuating the detrimental glial reaction [354].

Finally, Table 6 provides a comprehensive synthesis of the diverse and critical roles that astrocyte dysfunction assumes in PD pathogenesis. It systematically catalogs how these normally homeostatic glial cells transform into pathological drivers through specific mechanistic axes, including sustained neuroinflammation, glutamate excitotoxicity, oxidative stress, and the loss of neurotrophic support that collectively propel neurodegeneration. In parallel, the table delineates key-associated pathological components, such as α-synuclein propagation, metabolic dysregulation, and genetic susceptibility factors such as LRRK2 and GBA1 mutations. Crucially, for each identified mechanism, corresponding potential therapeutic strategies and illustrative research findings are presented, collectively reinforcing astrocytes as a central and multifaceted target for next-generation PD interventions.

**Table 6:** Astrocyte dysfunction domains and corresponding therapeutic interventions in Parkinson’s disease

Astrocytic pathological domain	Core molecular and cellular hallmarks	Interventional avenue	Critical translational evidence	References
1. Immuno-inflammatory crosstalk	Hyperactivation of NF-κB/STAT3 inflammatory cascades driving IL-1β, TNF-α, and IL-6 secretion; complement (C3/C1q) and purinergic (ATP/P2X7R) signaling loops; LRRK2-mutant priming of pro-inflammatory phenotypes	Pharmacological inhibition of upstream inflammatory kinases; application of anti-inflammatory modulators (e.g., NLY01); blockade of complement factors and P2X7R receptors; use of LRRK2 kinase inhibitors	Ablating astrocytic inflammatory signals halts dopaminergic loss in preclinical models; reciprocal astrocyte–microglia communication worsens neuronal injury; LRRK2-mutant astrocytes exhibit heightened α-synuclein exocytosis	[43, 103, 277, 355–360]
2. Proteostasis, clearance, and circadian control	Accumulation of misfolded α-synuclein with defective autophagy/lysosomal degradation (exacerbated by GBA1 dysfunction); dysregulation of core clock genes (BMAL1/CLOCK) and reduced melatonin signaling, impairing clearance rhythms	Induction of autophagic flux (e.g., rapamycin); small-molecule aggregation blockers (e.g., anle138b); GBA1 chaperones (e.g., ambroxol); chronotherapeutic agents (melatonin analogs) to restore circadian stability	Astrocytes internalize α-synuclein but lose degradative capacity over disease progression; the astrocytic circadian cycle directly modulates α-synuclein turnover, with rhythm disruption accelerating aggregate formation	[65, 251, 258, 358–361]
3. Redox, metabolic, and iron dyshomeostasis	Depleted Nrf2/ARE antioxidant defenses and glutathione loss; disruption of the astrocyte–neuron lactate shuttle (MCT1/4) alongside glycogen depletion and mitochondrial failure; iron overload from dysregulated ferritin/export mechanisms, promoting ferroptosis	Activation of Nrf2 (e.g., sulforaphane); metabolic rescue via lactate infusions or ketogenic dietary regimens; administration of iron-chelating agents (e.g., deferiprone)	Astrocyte-specific Nrf2 activation confers robust neuroprotection in synucleinopathy models; lactate derived from astrocytes is essential for neuronal survival; iron-accumulating astrocytes directly amplify oxidative vulnerability	[4, 362–367]
4. Trophic support and intercellular logistics	Decreased secretion of neurotrophic factors (GDNF, BDNF, TGF-β) and blunted Wnt/β-catenin signaling; impaired mitochondrial transfer from astrocytes to neurons due to disrupted tunneling nanotubes (TNTs) and Cx43 gap junctions	Gene therapy or protein delivery to replenish trophic factors; Wnt-signaling enhancers; upregulation of connexins to restore organelle trafficking between cells	Direct CNS administration of GDNF yields mixed clinical outcomes; however, in vitro evidence confirms that astrocyte-supplied mitochondria functionally rescue bioenergetically compromised neurons	[368–372]
5. Synaptic integrity and barrier maintenance	Deficient glutamate reuptake via EAAT1/GLT-1, leading to excessive mGluR stimulation; degradation of BBB tight-junction proteins (Claudin-5, ZO-1) driven by elevated MMP-9 and astrocytic VEGF secretion	Upregulation of GLT-1 expression (e.g., ceftriaxone); application of mGluR5 antagonists; MMP inhibition and tight-junction stabilization (e.g., angiopoietin-1)	Augmenting astrocytic glutamate clearance improves motor function in parkinsonian animal models; VEGF released by astrocytes directly correlates with BBB leakage observed in PD patients	[8, 154, 373–375]

**Fig. 5:**
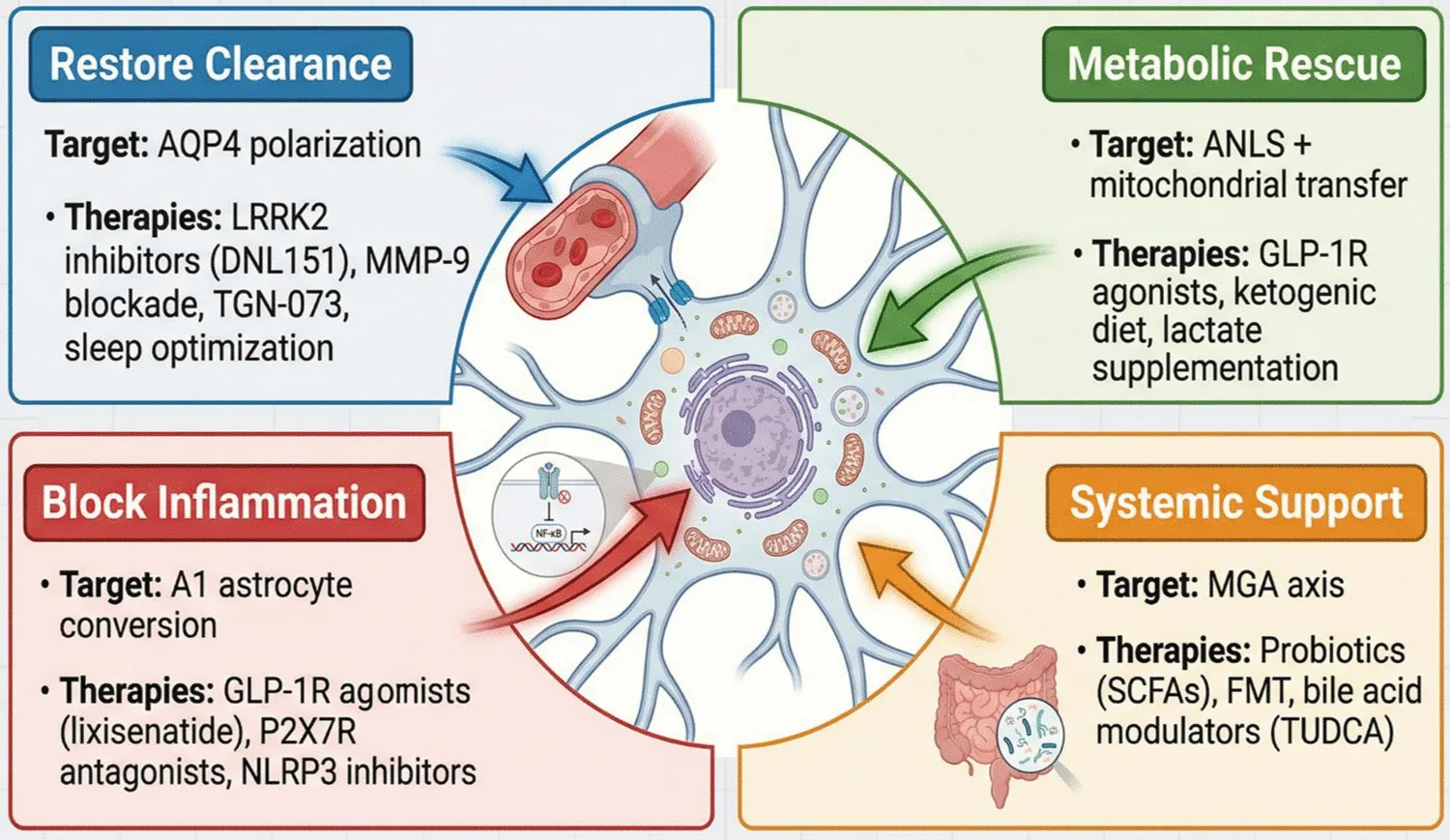
Multi-modal therapeutic strategies targeting astrocyte dysfunction in PD. Proposed intervention framework targeting AQP4 polarization, astrocyte conversion, metabolic dysfunction, and systemic support through pharmacological, dietary, and microbiome-based therapies

The therapeutic strategies outlined above converge on a multi-pronged approach aimed at restoring astrocyte homeostasis in PD. These interventions target key dysfunctional pathways, including AQP4-mediated glymphatic clearance, neuroinflammatory astrocyte conversion, metabolic support via lactate shuttling, and systemic modulation through the gut-brain axis. Figure 4 integrates these modalities into a cohesive framework, illustrating how combined pharmacological, lifestyle, and microbiome-based interventions may synergistically mitigate astrocytic dysfunction and modify disease progression (Fig. 5).

## Discussion

The evidence synthesized in this review fundamentally challenges the neuron-centric dogma of PD, repositioning the astrocyte from a passive support cell to a central architect of disease progression. While the loss of dopaminergic neurons remains the defining clinical endpoint, our analysis of the literature suggests that astrocytic failure is likely an upstream driver that creates the permissive environment for neurodegeneration. We acknowledge, however, that the existing evidence base, derived largely from cross-sectional human studies and acute toxin models, cannot definitively establish unidirectional causality. Figure 1 deliberately depicts a bidirectional network to reflect the reality that, in established PD, astrocyte dysfunction and neuronal degeneration engage in mutually reinforcing feed-forward loops. The critical unanswered question is whether astrocytes are the first responders or merely amplifiers of an initially neuronal insult. While temporal data from genetic carrier studies and incidental pathology favor an early astrocytic contribution, definitive proof will require longitudinal biomarker studies capable of tracking glial and neuronal health in parallel from the prodromal stage. We identify a vicious cycle of dysfunction where three core homeostatic pillars, proteostasis, metabolism, and immunomodulation, collapse simultaneously. The inability of astrocytes to clear α-syn via the lysosomal and glymphatic systems acts as the original sin, triggering a cascade where protein aggregation impairs mitochondrial respiration, which in turn fuels oxidative stress and forces the astrocyte into a reactive, pro-inflammatory state. This sequence suggests that therapeutic interventions targeting neurons alone may fail because they do not address the hostile microenvironment maintained by dysfunctional glia. A critical question arising from our synthesis is whether certain astrocytic dysfunctions represent primary pathogenic drivers versus secondary consequences of neuronal injury. Longitudinal evidence favors glymphatic failure (AQP4-depolarization) and lysosomal proteostasis impairment as primary mechanisms, as both precede detectable dopaminergic neuron loss in animal models. In contrast, the conversion to reactive astrocyte and lactate shuttle disruption consistently emerges after established protein aggregation, suggesting they amplify rather than initiate pathology. This distinction has direct translational implications: primary mechanisms are ideal targets for true disease modification in prodromal individuals, while secondary mechanisms may be more amenable to intervention in established disease. The field currently lacks prospective longitudinal studies in human subjects that could definitively establish this temporal hierarchy, representing a critical knowledge gap.

A recurring theme across the reviewed domains is the dual nature of the astrocytic response. In the prodromal and early stages of PD, astrocytes appear to mount a compensatory defense, upregulating Nrf2, increasing glycolytic flux to fuel neurons via the astrocyte-neuron lactate shuttle, and forming tunneling nanotubes to donate healthy mitochondria to distressed neurons. However, as pathology advances, this adaptive capacity is overwhelmed. The transition to a SASP and the loss of key trophic factors such as GDNF and BDNF mark a tipping point where the astrocyte effectively abandons its neuronal ward. This temporal dichotomy highlights a critical nuance for clinical translation: therapies must be stage-specific. Agents that broadly suppress astrocyte activation might be deleterious in early disease by dampening protective responses, whereas senolytic strategies that clear zombie astrocytes may be most effective in established disease where toxicity predominates.

This review further highlights that astrocyte dysfunction in PD is not confined to the nigrostriatal tract but is deeply integrated with systemic physiology. The emergence of the MGA axis provides a mechanistic link explaining how peripheral dysbiosis can drive central neuroinflammation. Gut-derived metabolites such as SCFAs, phenolic acids, and bacterial toxins bypass a compromised blood-brain barrier, itself a victim of astrocytic end-feet retraction and MMP release, to directly modulate glial reactivity. Furthermore, the disruption of circadian clock genes in astrocytes suggests that the non-motor symptoms of PD, such as sleep fragmentation, are not merely side effects but direct manifestations of glial pathology. This implies that restoring systemic homeostasis, such as through gut microbiota modulation or circadian entrainment, could serve as a viable strategy to reset astrocytic function and slow disease progression.

Finally, the therapeutic landscape reviewed here, ranging from GLP-1 receptor agonists to gene therapies like CRISPRa-mediated reprogramming, offers promise but faces significant translational hurdles. The success of agents like lixisenatide in Phase 2 trials validates the concept of targeting metabolic and inflammatory pathways in glia. However, the failure of other agents underscores the complexity of human PD compared to toxin-based rodent models. The disparity in receptor expression, like TLR4 differences between human and rodent astrocytes, and the lack of non-invasive biomarkers to quantify astrocytic reactivity in living patients, remain major bottlenecks. Future success will likely depend on “precision medicine” approaches that can stratify patients based on their specific profile of glial dysfunction, separating those driven primarily by inflammation from those driven by lysosomal or metabolic failure.

## Limitations and Future Directions

While the astrocyte-centric paradigm represents a transformative approach for PD therapeutics, several conceptual, methodological, and translational limitations must be acknowledged and addressed to realize its full potential. A primary limitation is the heavy reliance on rodent and toxin-induced models of PD, which fail to fully recapitulate the complex, protracted pathophysiology of human PD. A particularly consequential example is the TLR4 expression gap: TLR4 is highly expressed in human substantia nigra astrocytes but appears absent in rodent astrocytes, meaning that α-synuclein-TLR4-NF-κB inflammatory signaling, a cornerstone of reactive astrogliosis in human PD, cannot be adequately modeled in standard MPTP or 6-OHDA rodent paradigms. This mandates validation of all TLR-dependent mechanisms in human iPSC-derived astrocytes or 3D organoid systems before clinical translation. Key interspecies differences compromise the direct translation of mechanistic findings, necessitating a shift toward human-centric models such as iPSC-derived astrocytes and cerebral organoids. Furthermore, the vision of precision astrocyte therapies is constrained by a critical lack of validated biomarkers to quantify astrocytic states or dysfunctions in vivo. This absence impedes patient stratification and objective monitoring of therapeutic response, highlighting the urgent need for novel PET ligands, advanced MRI techniques, and biofluid assays.

A formidable conceptual challenge lies in the profound functional duality of astrocytes, which perform essential homeostatic roles while also being capable of neurotoxicity. Broadly suppressing astrocyte reactivity risks impairing their critical functions, making the objective not mere inhibition but precise phenotypic modulation. This requires a deeper molecular understanding of the signaling switches that control astrocyte phenotype. Additionally, the field is still early in comprehending the full spectrum of astrocyte heterogeneity and the temporal dynamics of their responses, which evolve with disease progression. An intervention beneficial in the prodromal stage may be inappropriate for advanced stages, underscoring the need for spatiotemporal mapping to inform stage-specific, regionally targeted therapies. Finally, astrocytes do not act in isolation but are integral to the neurovascular unit. Focusing therapies exclusively on astrocytes may overlook critical pathogenic feedback loops, suggesting that ultimate efficacy will likely require integrated, system-level interventions combining astrocyte-targeting agents with therapies for synergistic cell types.

In conclusion, overcoming these limitations requires a concerted shift toward human model validation, biomarker development, nuanced phenotypic modulation, and systems-level integration. By addressing these gaps, the “astrocyte paradigm” can evolve from a compelling concept into a cornerstone of next-generation, precision medicine for PD.

## Conclusion

In conclusion, the paradigm of PD is shifting from a purely neurodegenerative perspective to one that embraces a more holistic, multicellular view, with the astrocyte at its core. The evidence compellingly demonstrates that astrocyte dysfunction is not a secondary consequence but a primary driver of pathogenesis, orchestrating a vicious cycle of failed protein clearance, metabolic collapse, and chronic neuroinflammation that collectively seals the fate of dopaminergic neurons. The transition of astrocytes from neuroprotective guardians to neurotoxic agents represents a critical turning point in disease progression. Therefore, future therapeutic strategies must move beyond solely neuronal-focused interventions and embrace the development of astrocyte-targeted therapies. By restoring the homeostatic and supportive functions of these essential glial cells, it may be possible to slow or even halt the relentless progression of PD. Critically, the optimal window for astrocyte-directed disease-modifying therapy appears to be the prodromal and early motor stages, when astrocytes retain sufficient homeostatic reserve to be rescued before irreversible transition to a neurotoxic phenotype. Clinical trial designs must incorporate this temporal dimension through rigorous staging and biomarker-based patient stratification.

## Data Availability

No datasets were generated or analysed during the current study.

## Abbreviations

A1: Neurotoxic reactive astrocyte state
ANLS: Astrocyte-neuron lactate shuttle
BBB: Blood-brain barrier
C3: Complement component 3
CSF: Cerebrospinal fluid
EAAT2/GLT-1: Excitatory amino acid transporter 2
FFAR2/3: Free fatty acid receptors 2/3
GDNF: Glial cell line-derived neurotrophic factor
GLP-1: Glucagon-like peptide-1
IFN-γ: Interferon-gamma
IL-1β: Interleukin-1 beta
LD: Lipid droplet
LRRK2: Leucine-rich repeat kinase 2
MCT1/2/4: Monocarboxylate transporter 1/2/4
MMP-9: Matrix metalloproteinase-9
NLRP3: NLR family pyrin domain-containing 3
Nurr1: Nuclear receptor-related 1 protein
PD: Parkinson’s disease
SCFAs: Short-chain fatty acids
STAT3: Signal transducer and activator of transcription 3
TNF-α: Tumor necrosis factor-alpha
TUDCA: Tauroursodeoxycholic acid
VEGF: Vascular endothelial growth factor
6-OHDA: 6-Hydroxydopamine
ALP: Autophagy-lysosomal pathway
AQP4: Aquaporin-4
BDNF: Brain-derived neurotrophic factor
CNS: Central nervous system
Cx43: Connexin 43
ER: Endoplasmic reticulum
GBA1: Glucosylceramidase beta
GIP: Glucose-dependent insulinotropic polypeptide
GLP-1RA: GLP-1 receptor agonist
IL-1α: Interleukin-1 alpha
IL-6: Interleukin-6
LPS: Lipopolysaccharide
MAMP: Microbe-associated molecular pattern
MGA: Microbiota-gut-astrocyte axis
NF-κB: Nuclear factor kappa-light-chain-enhancer of activated B cells
Nrf2: Nuclear factor erythroid 2-related factor 2
P2X7R: Purinergic receptor P2X7
SASP: Senescence-associated secretory phenotype
SCN: Suprachiasmatic nucleus
TLR2/TLR4: Toll-like receptor 2/4
TNTs: Tunneling nanotubes
UPS: Ubiquitin-proteasome system
α-syn: Alpha-synuclein

## Declarations

### Declaration of AI Usage

No generative AI was used to create any figures. AI‑assisted design tools were used only for non‑generative layout refinement. All scientific content, data interpretation, mechanistic pathways, and final figure approval are solely the authors’ work. Quillbot was used for language refinement. The authors assume full responsibility for the accuracy, originality, and integrity of all content.

## References

[1] El-Sayed RM, Abdelaziz AM, Zaki HF, Abdel Rasheed NO (2023) Cilostazol novel neuroprotective mechanism against rotenone-induced Parkinson’s disease in rats: correlation between Nrf2 and HMGB1/TLR4/PI3K/Akt/mTOR signaling. Int Immunopharmacol 117. 10.1016/J.INTIMP.2023.109986 37012873

[2] Booth HDE, Hirst WD, Wade-Martins R (2017) The role of astrocyte dysfunction in Parkinson’s disease pathogenesis. Trends Neurosci 40:358. 10.1016/J.TINS.2017.04.001 28527591 PMC5462417

[3] Sorrentino ZA, Giasson BI, Chakrabarty P (2019) α-Synuclein and astrocytes: tracing the pathways from homeostasis to neurodegeneration in Lewy body disease. Acta Neuropathol 138:1. 10.1007/S00401-019-01977-2 30798354 PMC6571045

[4] Brash-Arias D, García LI, Pérez-Estudillo CA, Rojas-Durán F, Aranda-Abreu GE, Herrera-Covarrubias D, Chi-Castañeda D (2024) The role of astrocytes and alpha-synuclein in Parkinson’s disease: a review. NeuroSci 5:71. 10.3390/NEUROSCI5010005 39483813 PMC11523690

[5] Wang C, Yang T, Liang M, Xie J, Song N (2021) Astrocyte dysfunction in Parkinson’s disease: from the perspectives of transmitted α-synuclein and genetic modulation. Transl Neurodegener. 10.1186/S40035-021-00265-Y 34657636 PMC8522040

[6] Al-Qahtani Z, Al-kuraishy HM, Al-Gareeb AI, Albuhadily AK, Abdelaziz AM, Alruwaili M, El-Saber Batiha G (2026) Astrocytic dysfunction in Parkinson’s disease pathogenesis: focusing on alpha-synuclein. Inflammopharmacology. 10.1007/S10787-026-02176-8 41893948

[7] Satarker S, Bojja SL, Gurram PC, Mudgal J, Arora D, Nampoothiri M (2022) Astrocytic glutamatergic transmission and its implications in neurodegenerative disorders. Cells 11:1139. 10.3390/CELLS11071139 35406702 PMC8997779

[8] Mahmoud S, Gharagozloo M, Simard C, Gris D (2019) Astrocytes maintain glutamate homeostasis in the CNS by controlling the balance between glutamate uptake and release. Cells 8:184. 10.3390/CELLS8020184 30791579 PMC6406900

[9] Miyazaki I, Asanuma M (2020) Neuron-astrocyte interactions in Parkinson’s disease. Cells 9:2623. 10.3390/CELLS9122623 33297340 PMC7762285

[10] Niranjan R (2014) The role of inflammatory and oxidative stress mechanisms in the pathogenesis of Parkinson’s disease: focus on astrocytes. Mol Neurobiol 49:28–38. 10.1007/S12035-013-8483-X 23783559

[11] Kim J, Lee S, Hong DG, Yang S, Tran CS, Kwak J, Kim MJ, Rajarathinam T, Chung KW, Jung YS, Ishigami A, Chang SC, Lee H, Yun H, Lee J (2024) Amelioration of astrocyte-mediated neuroinflammation by EI-16004 confers neuroprotection in an MPTP-induced Parkinson’s disease model. Neuromolecular Med 26. 10.1007/S12017-023-08769-8 38294608

[12] Roumes H, Pellerin L, Bouzier-Sore AK (2023) Astrocytes as metabolic suppliers to support neuronal activity and brain functions. Essays Biochem 67:27. 10.1042/EBC20220080 36504117 PMC10011397

[13] Dringen R, Arend C (2025) Glutathione metabolism of the brain—the role of astrocytes. J Neurochem 169:e70073. 10.1111/JNC.70073 40313177 PMC12046376

[14] Alshahrani SM, Al-Kuraishy HM, Albuhadily AK, Al-Gareeb AI, Abdelaziz AM, Alexiou A, Papadakis M, Alruwaili M et al (2025) GDF15 upregulation in Parkinson’s disease: compensatory mechanisms and causal insights. Cytokine 193:156983. 10.1016/j.cyto.2025.156983 40580649

[15] de Rus Jacquet A, Alpaugh M, Denis HL, Tancredi JL, Boutin M, Decaestecker J, Beauparlant C, Herrmann L et al (2023) The contribution of inflammatory astrocytes to BBB impairments in a brain-chip model of Parkinson’s disease. Nat Commun 14(1):1–21. 10.1038/s41467-023-39038-8 PMC10282096 37339976

[16] Lau K, Kotzur R, Richter F (2024) Blood–brain barrier alterations and their impact on Parkinson’s disease pathogenesis and therapy. Transl Neurodegener 13:37. 10.1186/S40035-024-00430-Z 39075566 PMC11285262

[17] McConnell HL, Li Z, Woltjer RL, Mishra A (2019) Astrocyte dysfunction and neurovascular impairment in neurological disorders: correlation or causation? Neurochem Int 128:70. 10.1016/J.NEUINT.2019.04.005 30986503 PMC7728575

[18] Altay MF, Liu AKL, Holton JL, Parkkinen L, Lashuel HA (2022) Prominent astrocytic alpha-synuclein pathology with unique post-translational modification signatures unveiled across Lewy body disorders. Acta Neuropathol Commun. 10.1186/S40478-022-01468-8 36371251 PMC9652889

[19] Irwin DJ (2025) Where do PDD and DLB SYNdromes fit in neuronal alpha-SYNuclein biological frameworks?. J Neural Transm 133(2):133193–214. 10.1007/S00702-025-03060-5 PMC12855396 41343056

[20] Otero-Jimenez M, Wojewska MJ, Binding LP, Jogaudaite S, Gray-Rodriguez S, Young AL, Gentleman S, Alegre-Abarrategui J (2025) Neuropathological stages of neuronal, astrocytic and oligodendrocytic alpha-synuclein pathology in Parkinson’s disease. Acta Neuropathol Commun. 10.1186/S40478-025-01944-X 39934841 PMC11816504

[21] Sonninen TM, Hämäläinen RH, Koskuvi M, Oksanen M, Shakirzyanova A, Wojciechowski S, Puttonen K, Naumenko N et al (2020) Metabolic alterations in Parkinson’s disease astrocytes. Sci Rep 10(1):14474-. 10.1038/s41598-020-71329-8 PMC7468111 32879386

[22] Park AJ, Fish SL, Samstag CL, Kim M, Weiss J, Yu S, Callier ML, Chiu EH, Weiss J, Hampe CS, Estes RE, Lin B, Khera A, Yearout D, Pallanck LJ, Zabetian CP, Davis MY (2026) GBA1 deficiency differentially affects endolysosomal trafficking in neurons versus astrocytes. BioRxiv 2026.01.12.697563. 10.64898/2026.01.12.697563

[23] Zhang X, Wu H, Tang B, Guo J (2024) Clinical, mechanistic, biomarker, and therapeutic advances in GBA1-associated Parkinson’s disease. Transl Neurodegener 13(1):48. 10.1186/S40035-024-00437-6 39267121 PMC11391654

[24] Sun H, Liang R, Yang B, Zhou Y, Liu M, Fang F, Ding J, Fan Y et al (2016) Aquaporin-4 mediates communication between astrocyte and microglia: implications of neuroinflammation in experimental Parkinson’s disease. Neuroscience 317:65–75. 10.1016/j.neuroscience.2016.01.003 26774050

[25] Si X, Dai S, Fang Y, Tang J, Wang Z, Li Y, Song Z, Chen Y et al (2024) Matrix metalloproteinase-9 inhibition prevents aquaporin-4 depolarization-mediated glymphatic dysfunction in Parkinson’s disease. J Adv Res 56:125–136. 10.1016/j.jare.2023.03.004 PMC10834796 36940850

[26] Liddelow SA, Guttenplan KA, Clarke LE, Bennett FC, Bohlen CJ, Schirmer L, Bennett ML, Münch AE et al (2017) Neurotoxic reactive astrocytes are induced by activated microglia. Nature 541:481–487. 10.1038/nature21029 PMC5404890 28099414

[27] Ajoolabady A, Kim B, Abdulkhaliq AA, Ren J, Bahijri S, Tuomilehto J, Borai A, Khan J et al (2025) Dual role of microglia in neuroinflammation and neurodegenerative diseases. Neurobiol Dis 216:107133. 10.1016/j.nbd.2025.107133 41052547

[28] Li Y, Xia Y, Yin S, Wan F, Hu J, Kou L, Sun Y, Wu J et al (2021) Targeting microglial α-synuclein/TLRs/NF-kappaB/NLRP3 inflammasome axis in Parkinson’s disease. Front Immunol. 10.3389/fimmu.2021.719807 PMC8531525 34691027

[29] Wang C, Yang T, Liang M, Xie J, Song N (2021) Astrocyte dysfunction in Parkinson’s disease: from the perspectives of transmitted α-synuclein and genetic modulation. Transl Neurodegener 10:39. 10.1186/S40035-021-00265-Y 34657636 PMC8522040

[30] Hughes CD, Choi ML, Ryten M, Hopkins L, Drews A, Botía JA, Iljina M, Rodrigues M et al (2019) Picomolar concentrations of oligomeric alpha-synuclein sensitizes TLR4 to play an initiating role in Parkinson’s disease pathogenesis. Acta Neuropathol 137:103–120. 10.1007/s00401-018-1907-y PMC6338693 30225556

[31] Pike AF, Varanita T, Herrebout MAC, Plug BC, Kole J, Musters RJP, Teunissen CE, Hoozemans JJM et al (2021) α‐Synuclein evokes NLRP3 inflammasome‐mediated IL‐1β secretion from primary human microglia. Glia 69:1413. 10.1002/GLIA.23970 PMC8247862 33506583

[32] Abdelaziz AM, Rasheed NOA, Zaki HF, Salem HA, El-Sayed RM (2024) Canagliflozin attenuates neurodegeneration and ameliorates dyskinesia through targeting the NLRP3/Nurr1/GSK-3β/SIRT3 pathway and autophagy modulation in rotenone-lesioned rats. Int Immunopharmacol 146. 10.1016/J.INTIMP.2024.113839 39700958

[33] Zou W, Kou L, Wang Y, Jin Z, Xiong N, Wang T, Xia Y (2025) Complement C4 exacerbates astrocyte-mediated neuroinflammation and promotes α-synuclein pathology in Parkinson’s disease. Npj Parkinson’s Disease 11(1):11 1–14. 10.1038/s41531-025-01005-z PMC12119883 40436856

[34] Du RH, Zhou Y, Xia ML, Lu M, Ding JH, Hu G (2018) α-Synuclein disrupts the anti-inflammatory role of Drd2 via interfering β-arrestin2-TAB1 interaction in astrocytes. J Neuroinflammation 15:258. 10.1186/s12974-018-1302-6 30200997 PMC6131810

[35] Datta D, Leslie SN, Morozov YM, Duque A, Rakic P, Van Dyck CH, Nairn AC, Arnsten AFT (2020) Classical complement cascade initiating C1q protein within neurons in the aged rhesus macaque dorsolateral prefrontal cortex. J Neuroinflammation 17(1):8. 10.1186/S12974-019-1683-1 31906973 PMC6945481

[36] Zhao J, Li Q, Ouyang X, Wang F, Li Q, Xu Z, Ji D, Wu Q et al (2023) The effect of CX3CL1/CX3CR1 signal axis on microglia in central nervous system diseases. Journal of Neurorestoratology 11:100042. 10.1016/J.JNRT.2023.100042

[37] Hong S, Beja-Glasser VF, Nfonoyim BM, Frouin A, Li S, Ramakrishnan S, Merry KM, Shi Q et al (1979) Complement and microglia mediate early synapse loss in Alzheimer mouse models. Science 352:712–716. 10.1126/SCIENCE.AAD8373 PMC5094372 27033548

[38] Angelopoulou E, Paudel YN, Shaikh MF, Piperi C (2020) Fractalkine (CX3CL1) signaling and neuroinflammation in Parkinson’s disease: potential clinical and therapeutic implications. Pharmacol Res 158:104930. 10.1016/J.PHRS.2020.104930 32445958

[39] Shigetomi E, Sakai K, Koizumi S (2024) Extracellular ATP/adenosine dynamics in the brain and its role in health and disease. Front Cell Dev Biol 11:1343653. 10.3389/FCELL.2023.1343653 38304611 PMC10830686

[40] Islam J, Cho JA, Kim JY, Park KS, Koh YJ, Chung CY, Lee EJ, Nam SJ, et al. (2022) GPCR19 regulates P2X7R-mediated NLRP3 inflammasomal activation of microglia by amyloid β in a mouse model of Alzheimer’s disease. Front Immunol 13:766919. 10.3389/FIMMU.2022.766919/BIBTEX 35464490 PMC9019633

[41] Bedetta M, Pizzo P, Lia A (2025) The multifaceted role of P2X7R in microglia and astrocytes. Neurochem Res 50:239. 10.1007/S11064-025-04502-Y 40690063 PMC12279596

[42] Albalawi F, Lu W, Beckel JM, Lim JC, McCaughey SA, Mitchell CH (2017) The P2X7 receptor primes IL-1β and the NLRP3 inflammasome in astrocytes exposed to mechanical strain. Front Cell Neurosci 11:227. 10.3389/FNCEL.2017.00227/FULL 28848393 PMC5550720

[43] Kam TI, Hinkle JT, Dawson TM, Dawson VL (2020) Microglia and astrocyte dysfunction in Parkinson’s disease. Neurobiol Dis 144:105028. 10.1016/J.NBD.2020.105028 32736085 PMC7484088

[44] Chen K, Wang H, Ilyas I, Mahmood A, Hou L (2023) Microglia and astrocytes dysfunction and key neuroinflammation-based biomarkers in Parkinson’s disease. Brain Sci 13:634. 10.3390/BRAINSCI13040634 37190599 PMC10136556

[45] Rostami J, Fotaki G, Sirois J, Mzezewa R, Bergström J, Essand M, Healy L, Erlandsson A (2020) Astrocytes have the capacity to act as antigen-presenting cells in the Parkinson’s disease brain. J Neuroinflammation 17:119. 10.1186/s12974-020-01776-7 32299492 PMC7164247

[46] Abounit S, Bousset L, Loria F, Zhu S, de Chaumont F, Pieri L, Olivo‐Marin J, Melki R et al (2016) Tunneling nanotubes spread fibrillar α‐synuclein by intercellular trafficking of lysosomes. EMBO J 35:2120–2138. 10.15252/EMBJ.201593411 PMC5048354 27550960

[47] Russ K, Teku G, Bousset L, Redeker V, Piel S, Savchenko E, Pomeshchik Y, Savistchenko J et al (2021) TNF-α and α-synuclein fibrils differently regulate human astrocyte immune reactivity and impair mitochondrial respiration. Cell Rep 34:108895. 10.1016/j.celrep.2021.108895 33761362

[48] Skou LD, Johansen SK, Okarmus J, Meyer M (2024) Pathogenesis of DJ-1/PARK7-mediated Parkinson’s disease. Cells 13:296. 10.3390/CELLS13040296 38391909 PMC10887164

[49] Ariga H, Takahashi-Niki K, Kato I, Maita H, Niki T, Iguchi-Ariga SMM (2013) Neuroprotective function of DJ-1 in Parkinson’s disease. Oxid Med Cell Longev. 10.1155/2013/683920 23766857 PMC3671546

[50] Choi DJ, An J, Jou I, Park SM, Joe EH (2019) A Parkinson’s disease gene, DJ-1, regulates anti-inflammatory roles of astrocytes through prostaglandin D2 synthase expression. Neurobiol Dis 127:482–491. 10.1016/J.NBD.2019.04.003 30954702

[51] Kim J, Choi D, Jeong H, Kim J, Kim DW, Choi SY, Park SM, Suh YH et al (2013) DJ-1 facilitates the interaction between STAT1 and its phosphatase, SHP-1, in brain microglia and astrocytes: a novel anti-inflammatory function of DJ-1. Neurobiol Dis 60:1–10. 10.1016/j.nbd.2013.08.007 23969237

[52] Waak J, Weber SS, Waldenmaier A, Görner K, Alunni-Fabbroni M, Schell H, Vogt-Weisenhorn D, Pham TT et al (2009) Regulation of astrocyte inflammatory responses by the Parkinson’s disease-associated gene DJ-1. FASEB J 23:2478–2489. 10.1096/FJ.08-125153 19276172

[53] De Miranda BR, Rocha EM, Bai Q, El Ayadi A, Hinkle D, Burton EA, Timothy Greenamyre J (2018) Astrocyte-specific DJ-1 overexpression protects against rotenone-induced neurotoxicity in a rat model of Parkinson’s disease. Neurobiol Dis 115:101. 10.1016/J.NBD.2018.04.008 PMC5943150 29649621

[54] Wei S, Song X, Mou Y, Yang T, Wang Y, Wang H, Ren C, Song X (2025) New insights into pathogenisis and therapies of P2X7R in Parkinson’s disease. NPJ Parkinsons Dis 11:108. 10.1038/S41531-025-00980-7 40325043 PMC12053563

[55] Ciechanover A, Kwon YT (2015) Degradation of misfolded proteins in neurodegenerative diseases: therapeutic targets and strategies. Exp Mol Med 47:e147–e147. 10.1038/EMM.2014.117;SUBJMETA 25766616 PMC4351408

[56] Lehtonen Š, Sonninen TM, Wojciechowski S, Goldsteins G, Koistinaho J (2019) Dysfunction of cellular proteostasis in Parkinson’s disease. Front Neurosci 13:457790. 10.3389/FNINS.2019.00457/FULL PMC6524622 31133790

[57] Sharma A, Mannan A, Singh TG (2025) Rethinking Parkinson’s: the role of proteostasis networks and autophagy in disease progression. Mol Cell Neurosci 134:104023. 10.1016/J.MCN.2025.104023 40490236

[58] Lee HJ, Suk JE, Patrick C, Bae EJ, Cho JH, Rho S, Hwang D, Masliah E et al (2010) Direct transfer of α-synuclein from neuron to astroglia causes inflammatory responses in synucleinopathies. J Biol Chem 285:9262–9272. 10.1074/jbc.M109.081125 PMC2838344 20071342

[59] Senol AD, Samarani M, Syan S, Guardia CM, Nonaka T, Liv N, Latour-Lambert P, Hasegawa M et al (2021) A-Synuclein fibrils subvert lysosome structure and function for the propagation of protein misfolding between cells through tunneling nanotubes. PLoS Biol. 10.1371/JOURNAL.PBIO.3001287 PMC8291706 34283825

[60] Rahmani Z, Surabhi S, Rojo-Cortés F, Dulac A, Jenny A, Birman S (2022) Lamp1 deficiency enhances sensitivity to α-synuclein and oxidative stress in *Drosophila* models of Parkinson disease. Int J Mol Sci. 10.3390/IJMS232113078 36361864 PMC9657416

[61] Klucken J, Poehler AM, Ebrahimi-Fakhari D, Schneider J, Nuber S, Rockenstein E, Schlötzer-Schrehardt U, Hyman BT et al (2012) Alpha-synuclein aggregation involves a bafilomycin A1-sensitive autophagy pathway. Autophagy 8:754. 10.4161/AUTO.19371 PMC3378419 22647715

[62] Riedel M, Goldbaum O, Richter-Landsberg C (2009) α-Synuclein promotes the recruitment of tau to protein inclusions in oligodendroglial cells: effects of oxidative and proteolytic stress. J Mol Neurosci 39:226–234. 10.1007/S12031-009-9190-Y 19266322

[63] Kim YM, Jang WH, Quezado MM, Oh Y, Chung KC, Junn E, Mouradian MM (2011) Proteasome inhibition induces α-synuclein SUMOylation and aggregate formation. J Neurol Sci 307:157. 10.1016/J.JNS.2011.04.015 21641618 PMC3129438

[64] Hua J, Yin N, Yang B, Zhang J, Ding J, Fan Y, Hu G (2017) Ginkgolide B and bilobalide ameliorate neural cell apoptosis in α-synuclein aggregates. Biomed Pharmacother 96:792–797. 10.1016/j.biopha.2017.10.050 29054095

[65] Tsunemi T, Ishiguro Y, Yoroisaka A, Valdez C, Miyamoto K, Ishikawa K, Saiki S, Akamatsu W et al (2020) Astrocytes protect human dopaminergic neurons from α-synuclein accumulation and propagation. J Neurosci 40:8618. 10.1523/JNEUROSCI.0954-20.2020 PMC7643299 33046546

[66] Rostami J, Holmqvist S, Lindström V, Sigvardson J, Westermark GT, Ingelsson M, Bergström J, Roybon L et al (2017) Human astrocytes transfer aggregated alpha-synuclein via tunneling nanotubes. J Neurosci 37:11835–11853. 10.1523/JNEUROSCI.0983-17.2017 PMC5719970 29089438

[67] Giusti V, Kaur G, Giusto E, Civiero L (2024) Brain clearance of protein aggregates: a close-up on astrocytes. Mol Neurodegener. 10.1186/S13024-024-00703-1 38229094 PMC10790381

[68] Lindström V, Gustafsson G, Sanders LH, Howlett EH, Sigvardson J, Kasrayan A, Ingelsson M, Bergström J et al (2017) Extensive uptake of α-synuclein oligomers in astrocytes results in sustained intracellular deposits and mitochondrial damage. Mol Cell Neurosci 82:143–156. 10.1016/J.MCN.2017.04.009 28450268

[69] Ho MS (2025) Clearance pathways for α-synuclein in Parkinson’s disease. J Neurochem 169:e70124. 10.1111/JNC.70124;JOURNAL:JOURNAL:14714159;WGROUP:STRING:PUBLICATION 40509661 PMC12163304

[70] Streubel-Gallasch L, Giusti V, Sandre M, Tessari I, Plotegher N, Giusto E, Masato A, Iovino L et al (2021) Parkinson’s disease-associated LRRK2 interferes with astrocyte-mediated alpha-synuclein clearance. Mol Neurobiol 58:3119–3140. 10.1007/S12035-021-02327-8 PMC8257537 33629273

[71] Obergasteiger J, Frapporti G, Lamonaca G, Pizzi S, Picard A, Lavdas AA, Pischedda F, Piccoli G, Hilfiker S, Lobbestael E, Baekelandt V, Hicks AA, Corti C, Pramstaller PP, Volta M (2020) Kinase inhibition of G2019S-LRRK2 enhances autolysosome formation and function to reduce endogenous alpha-synuclein intracellular inclusions. Cell Death Discov 6(1):1–13. 10.1038/s41420-020-0279-y PMC7280235 32550012

[72] Henry AG, Aghamohammadzadeh S, Samaroo H, Chen Y, Mou K, Needle E, Hirst WD (2015) Pathogenic LRRK2 mutations, through increased kinase activity, produce enlarged lysosomes with reduced degradative capacity and increase ATP13A2 expression. Hum Mol Genet 24:6013–6028. 10.1093/HMG/DDV314 26251043

[73] Sanyal A, DeAndrade MP, Novis HS, Lin S, Chang J, Lengacher N, Tomlinson JJ, Tansey MG et al (2020) Lysosome and inflammatory defects in GBA1-mutant astrocytes are normalized by LRRK2 inhibition. Mov Disord 35:760–773. 10.1002/MDS.27994 PMC8167931 32034799

[74] Kwatra M, Kwak G, Li H, Suk JS, Ko HS (2025) Polymeric nanoparticle-mediated GBA1 gene therapy is neuroprotective in a preclinical model of Parkinson’s disease. Drug Deliv Transl Res 2025:1–17. 10.1007/S13346-025-01944-3 PMC12426995 40836186

[75] Kim S, Yun SP, Lee S, Umanah GE, Bandaru VVR, Yin X, Rhee P, Karuppagounder SS et al (2018) GBA1 deficiency negatively affects physiological α-synuclein tetramers and related multimers. Proc Natl Acad Sci U S A 115:798–803. 10.1073/PNAS.1700465115 PMC5789900 29311330

[76] Qiao C, Yin N, Gu HY, Zhu JL, Ding JH, Lu M, Hu G (2016) Atp13a2 Deficiency aggravates astrocyte-mediated neuroinflammation via NLRP3 inflammasome activation. CNS Neurosci Ther 22:451–460. 10.1111/CNS.12514 26848562 PMC6492810

[77] Fujii T, Nagamori S, Wiriyasermkul P, Zheng S, Yago A, Shimizu T, Tabuchi Y, Okumura T, Fujii T, Takeshima H, Sakai H (2023) Parkinson’s disease-associated ATP13A2/PARK9 functions as a lysosomal H+,K+-ATPase. Nat Commun 14(1):1–11. 10.1038/s41467-023-37815-z PMC10119128 37080960

[78] O’Hara DM, Pawar G, Kalia SK, Kalia LV (2020) LRRK2 and α-synuclein: distinct or synergistic players in Parkinson’s disease? Front Neurosci 14:577. 10.3389/FNINS.2020.00577 32625052 PMC7311858

[79] Sonninen TM, Hämäläinen RH, Koskuvi M, Oksanen M, Shakirzyanova A, Wojciechowski S, Puttonen K, Naumenko N et al (2020) Metabolic alterations in Parkinson’s disease astrocytes. Sci Rep 10:14474. 10.1038/S41598-020-71329-8 PMC7468111 32879386

[80] Huang H, Lin L, Wu T, Wu C, Zhou L, Li G, Su F, Liang F et al (2024) Phosphorylation of AQP4 by LRRK2 R1441G impairs glymphatic clearance of IFNγ and aggravates dopaminergic neurodegeneration. NPJ Parkinsons Dis 10:31. 10.1038/S41531-024-00643-Z PMC10831045 38296953

[81] Yi S, Wang L, Wang H, Ho MS, Zhang S (2022) Pathogenesis of α-synuclein in Parkinson’s disease: from a neuron-glia crosstalk perspective. Int J Mol Sci 23:14753. 10.3390/IJMS232314753 36499080 PMC9739123

[82] Kim S, Pajarillo E, Nyarko-Danquah I, Aschner M, Lee E (2023) Role of astrocytes in Parkinson’s disease associated with genetic mutations and neurotoxicants. Cells 12:622. 10.3390/CELLS12040622 36831289 PMC9953822

[83] Lambourne OA, Bell S, Wilhelm LP, Yarbrough EB, Holly GG, Russell OM, Alghamdi AM, Fdel AM et al (2023) PINK1-dependent mitophagy inhibits elevated ubiquitin phosphorylation caused by mitochondrial damage. J Med Chem 66:7645–7656. 10.1021/ACS.JMEDCHEM.3C00555/SUPPL_FILE/JM3C00555_SI_001.CSV PMC10258795 37248632

[84] Riley JF, Robbins CV, Holzbaur ELF (2026) PINK1/Parkin-dependent mitophagy mediates astrocytic inflammatory responses to mitochondrial damage. BioRxiv 2026.05.11.724378. 10.64898/2026.05.11.724378

[85] Franco R, Rivas-Santisteban R, Navarro G, Pinna A, Reyes-Resina I (2021) Genes implicated in familial Parkinson’s disease provide a dual picture of nigral dopaminergic neurodegeneration with mitochondria taking center stage. Int J Mol Sci 22:4643. 10.3390/IJMS22094643/S1 33924963 PMC8124903

[86] Ge P, Dawson VL, Dawson TM (2020) PINK1 and Parkin mitochondrial quality control: a source of regional vulnerability in Parkinson’s disease. Mol Neurodegener 15:20. 10.1186/S13024-020-00367-7 32169097 PMC7071653

[87] Murphy KE, Gysbers AM, Abbott SK, Tayebi N, Kim WS, Sidransky E, Cooper A, Garner B et al (2014) Reduced glucocerebrosidase is associated with increased α-synuclein in sporadic Parkinson’s disease. Brain 137:834–848. 10.1093/BRAIN/AWT367 PMC3927701 24477431

[88] Blanz J, Saftig P (2016) Parkinson’s disease: acid-glucocerebrosidase activity and alpha-synuclein clearance. J Neurochem 139:198–215. 10.1111/JNC.13517;WGROUP:STRING:PUBLICATION 26860955

[89] Bae EJ, Yang NY, Lee C, Lee HJ, Kim S, Sardi SP, Lee SJ (2015) Loss of glucocerebrosidase 1 activity causes lysosomal dysfunction and α-synuclein aggregation. Exp Mol Med 47(3):e153–e153. 10.1038/emm.2014.128 25813221 PMC4351412

[90] Liu G, Aliaga L, Cai H (2012) α-synuclein, LRRK2 and their interplay in Parkinson’s disease. Future Neurol 7:145–153. 10.2217/FNL.12.2 22563296 PMC3343692

[91] rui Sun Y, Lv QK, Liu JY, Wang F, Liu CF (2025) New perspectives on the glymphatic system and the relationship between glymphatic system and neurodegenerative diseases. Neurobiol Dis 205 106791. 10.1016/J.NBD.2025.106791 39778750

[92] De la Herrán-Arita AK (2025) When sleep fails, brain clearance suffers: the role of glymphatic impairment in clinical neurology. Acta Neurol Belg. 10.1007/S13760-025-02959-W 41315137

[93] Chen S, Wang H, Zhang L, Xi Y, Lu Y, Yu K, Zhu Y, Regina I et al (2025) Glymphatic system: a self-purification circulation in brain. Front Cell Neurosci 19:1528995. 10.3389/FNCEL.2025.1528995/BIBTEX PMC11861344 40012567

[94] Abdelaziz AM (2026) The cGAS-STING-glymphatic-gut axis in Parkinson’s disease: a proposed self-amplifying triad of neuroinflammation and therapeutic opportunity. Int Immunopharmacol 179:116628. 10.1016/J.INTIMP.2026.116628 41966779

[95] Wang L, Tanglay O, Su F, Li H, Liu J, Kim WS, Halliday GM, Fu YH (2025) Distinct AQP4 alterations in movement disorders with primary synucleinopathy. Mov Disord. 10.1002/MDS.70023;WEBSITE:WEBSITE:MOVEMENTDISORDERS;JOURNAL:JOURNAL:15318257;REQUESTEDJOURNAL:JOURNAL:15318257;WGROUP:STRING:PUBLICATION 40862511 PMC12710156

[96] Lian X, Liu Z, Gan Z, Yan Q, Tong L, Qiu L, Liu Y, Chen JF et al (2025) Targeting the glymphatic system to promote α-synuclein clearance: a novel therapeutic strategy for Parkinson’s disease. Neural Regen Res 21:233. 10.4103/NRR.NRR-D-24-00764 PMC12094544 39819820

[97] Lopes DM, Llewellyn SK, Bury SE, Wang J, Wells JA, Gegg ME, Verona G, Lythgoe MF, et al. (2025) The influence of the glymphatic system on α-synuclein propagation: the role of aquaporin-4. Brain 148:4519–4531. 10.1093/BRAIN/AWAF255 40632813

[98] Qin J, Fang Y, Duanmu X, Wen J, Wu H, Wu C, Guo T, Zhou C, Zheng Q, Yuan W, Zhu Z, Chen J, Wu J, Jiang M, Zhang B, Xu X, Guan X, Zhang M (2025) The effects of aquaporin-4 polymorphisms on glymphatic function and motor symptoms in Parkinson’s disease. Npj Parkinson’s Disease 11(1):288. 10.1038/s41531-025-01139-0 PMC12508451 41062548

[99] Huang H, Lin L, Wu T, Wu C, Zhou L, Li G, Su F, Liang F et al (2024) Phosphorylation of AQP4 by LRRK2 R1441G impairs glymphatic clearance of IFNγ and aggravates dopaminergic neurodegeneration. NPJ Parkinsons Dis 10:14. 10.1038/s41531-024-00643-z PMC10831045 38296953

[100] Braun M, Simon MJ, Jang J, Sanderson K, Swierz J, Sevao M, Pincus AB, Schaser AJ, Elliott JE, Lim MM, Unni VK, Schindler AG, Keene CD, Latimer CS, Iliff JJ (2024) Aquaporin-4 mis-localization slows glymphatic clearance of α-synuclein and promotes α-synuclein pathology and aggregate propagation. BioRxiv 2024.08.14.607971. 10.1101/2024.08.14.607971

[101] Ding XB, Wang XX, Xia DH, Liu H, Tian HY, Fu Y, Chen YK, Qin C et al (2021) Impaired meningeal lymphatic drainage in patients with idiopathic Parkinson’s disease. Nat Med 27:411–418. 10.1038/s41591-020-01198-1 33462448

[102] Chen L, Shen Q, Liu Y, Zhang Y, Sun L, Ma X, Song N, Xie J (2025) Homeostasis and metabolism of iron and other metal ions in neurodegenerative diseases. Signal Transduct Target Ther 10(1):1–48. 10.1038/s41392-024-02071-0 39894843 PMC11788444

[103] Oksanen M, Lehtonen S, Jaronen M, Goldsteins G, Hämäläinen RH, Koistinaho J (2019) Astrocyte alterations in neurodegenerative pathologies and their modeling in human induced pluripotent stem cell platforms. Cell Mol Life Sci 76(14):2739–2760. 10.1007/S00018-019-03111-7 31016348 PMC6588647

[104] Franklin H, Clarke BE, Patani R (2021) Astrocytes and microglia in neurodegenerative diseases: lessons from human in vitro models. Prog Neurobiol 200:101973. 10.1016/J.PNEUROBIO.2020.101973 33309801 PMC8052192

[105] Cui J, Guo X, Li Q, Song N, Xie J (2020) Hepcidin-to-ferritin ratio is decreased in astrocytes with extracellular alpha-synuclein and iron exposure. Front Cell Neurosci 14:47. 10.3389/FNCEL.2020.00047 32210768 PMC7075942

[106] Chen L, Shen Q, Liu Y, Zhang Y, Sun L, Ma X, Song N, Xie J (2025) Homeostasis and metabolism of iron and other metal ions in neurodegenerative diseases. Signal Transduct Target Ther 10(1):1–48. 10.1038/s41392-024-02071-0 PMC11788444 39894843

[107] You L, Yu PP, Dong T, Guo W, Chang S, Zheng B, Ci Y, Wang F, Yu P, Gao G, Chang YZ (2022) Astrocyte-derived hepcidin controls iron traffic at the blood-brain-barrier via regulating ferroportin 1 of microvascular endothelial cells. Cell Death Dis 13. 10.1038/S41419-022-05043-W PMC9343463 35915080

[108] Zhang X, Gou YJ, Zhang Y, Li J, Han K, Xu Y, Li H, You LH et al (2020) Hepcidin overexpression in astrocytes alters brain iron metabolism and protects against amyloid-β induced brain damage in mice. Cell Death Discov 6:113. 10.1038/S41420-020-00346-3 PMC7603348 33298837

[109] Liang T, Qian ZM, Mu MD, Yung WH, Ke Y (2020) Brain hepcidin suppresses major pathologies in experimental parkinsonism. IScience 23:101284. 10.1016/J.ISCI.2020.101284 32623334 PMC7334576

[110] Xu M, Tan X, Li N, Wu H, Wang Y, Xie J, Wang J (2019) Differential regulation of estrogen in iron metabolism in astrocytes and neurons. J Cell Physiol 234:4232–4242. 10.1002/JCP.27188 30132882

[111] Ru Q, Li Y, Chen L,Wu Y, Min J, Wang F (2024) Iron homeostasis and ferroptosis in human diseases: mechanisms and therapeutic prospects, Signal Transduction and Targeted Therapy 9(1): 1–64. 10.1038/s41392-024-01969-z PMC11486532 39396974

[112] Kim Y, Connor JR (2020) The roles of iron and HFE genotype in neurological diseases. Mol Aspects Med 75:100867. 10.1016/J.MAM.2020.100867 32654761

[113] Marshall Moscon SL, Connor JR (2024) HFE Mutations in neurodegenerative disease as a model of hormesis. Int J Mol Sci 25:3334. 10.3390/IJMS25063334 PMC10970347 38542306

[114] Izumi Y, Yamamoto N, Matsushima S, Yamamoto T, Takada-Takatori Y, Akaike A, Kume T (2015) Compensatory role of the Nrf2–ARE pathway against paraquat toxicity: relevance of 26S proteasome activity. J Pharmacol Sci 129:150–159. 10.1016/J.JPHS.2015.09.003 26598004

[115] Song X, Long D (2020) Nrf2 and ferroptosis: a new research direction for neurodegenerative diseases. Front Neurosci 14:484266. 10.3389/FNINS.2020.00267/FULL PMC7186402 32372896

[116] Thomas GEC, Zarkali A, Ryten M, Shmueli K, Gil-Martinez AL, Leyland LA, McColgan P, Acosta-Cabronero J et al (2021) Regional brain iron and gene expression provide insights into neurodegeneration in Parkinson’s disease. Brain 144:1787. 10.1093/BRAIN/AWAB084 PMC8320305 33704443

[117] Dringen R, Karger G, Winkler U, Hirrlinger J (2025) ATP metabolism of astrocytes: consumption, regeneration and restoration. Neurochem Res 50(6):1–26. 10.1007/S11064-025-04604-7 PMC12612006 41222868

[118] Mulica P, Grünewald A, Pereira SL (2021) Astrocyte-neuron metabolic crosstalk in neurodegeneration: a mitochondrial perspective. Front. Endocrinol. (Lausanne) 12:668517. 10.3389/fendo.2021.668517 34025580 PMC8138625

[119] Wang X, Becker K, Levine N, Zhang M, Lieberman AP, Moore DJ, Ma J (2019) Pathogenic alpha-synuclein aggregates preferentially bind to mitochondria and affect cellular respiration. Acta Neuropathol Commun 7:41. 10.1186/S40478-019-0696-4 30871620 PMC6419482

[120] Bellingacci L, Sciaccaluga M, Megaro A, Cardinale A, Canonichesi J, De Carluccio M, Mastrantonio R, Costa C et al (2025) Oligomeric alpha-synuclein causes early synaptic dysfunction of the corticostriatal pathway associated with non-motor symptoms. NPJ Parkinsons Dis 11:220. 10.1038/S41531-025-01075-Z PMC12307689 40730592

[121] Gustafsson G, Lindström V, Rostami J, Nordström E, Lannfelt L, Bergström J, Ingelsson M, Erlandsson A (2017) Alpha-synuclein oligomer-selective antibodies reduce intracellular accumulation and mitochondrial impairment in alpha-synuclein exposed astrocytes. Journal of Neuroinflammation 14(1)241. 10.1186/S12974-017-1018-Z PMC5725978 29228971

[122] Xie W, Chung KKK (2012) Alpha-synuclein impairs normal dynamics of mitochondria in cell and animal models of Parkinson’s disease. J Neurochem 122:404–414. 10.1111/J.1471-4159.2012.07769.X 22537068

[123] Hayakawa K, Esposito E, Wang X, Terasaki Y, Liu Y, Xing C, Ji X, Lo EH (2016) Transfer of mitochondria from astrocytes to neurons after stroke. Nature 535:551. 10.1038/NATURE18928 27466127 PMC4968589

[124] Cheng XY, Biswas S, Li J, Mao CJ, Chechneva O, Chen J, Li K, Li J, Zhang JR, Liu CF, Bin Deng W (2020) Human iPSCs derived astrocytes rescue rotenone-induced mitochondrial dysfunction and dopaminergic neurodegeneration in vitro by donating functional mitochondria. Transl Neurodegener 9:13. 10.1186/S40035-020-00190-6 PMC7325238 32345341

[125] Kuter KZ, Olech Ł, Dencher NA (2019) Increased energetic demand supported by mitochondrial electron transfer chain and astrocyte assistance is essential to maintain the compensatory ability of the dopaminergic neurons in an animal model of early Parkinson’s disease. Mitochondrion 47:227–237. 10.1016/J.MITO.2018.12.002 30578987

[126] Scheiblich H, Eikens F, Wischhof L, Opitz S, Jüngling K, Cserép C, Schmidt SV, Lambertz J et al (2024) Microglia rescue neurons from aggregate-induced neuronal dysfunction and death through tunneling nanotubes. Neuron 112:3106-3125.e8. 10.1016/J.NEURON.2024.06.029 39059388

[127] Neher JJ, Simons M (2024) Protective lifelines: tunneling nanotubes connect neurons and microglia. Neuron 112:2991–2993. 10.1016/J.NEURON.2024.07.027 39326386

[128] Joshi AU, Minhas PS, Liddelow SA, Haileselassie B, Andreasson KI, Dorn GW, Mochly-Rosen D (2019) Fragmented mitochondria released from microglia trigger A1 astrocytic response and propagate inflammatory neurodegeneration. Nat Neurosci 22:1635–1648. 10.1038/S41593-019-0486-0 31551592 PMC6764589

[129] Vasquez V, Kodavati M, Mitra J, Vedula I, Hamilton DJ, Garruto RM, Rao KS, Hegde ML (2024) Mitochondria-targeted oligomeric α-synuclein induces TOM40 degradation and mitochondrial dysfunction in Parkinson’s disease and parkinsonism-dementia of Guam. Cell Death Dis. 10.1038/S41419-024-07258-5 39695091 PMC11655978

[130] Jeon YM, Kwon Y, Jo M, Lee S, Kim S, Kim HJ (2020) The role of glial mitochondria in α-synuclein toxicity. Front Cell Dev Biol 8:548283. 10.3389/FCELL.2020.548283/FULL 33262983 PMC7686475

[131] Chen MM, Hu ZL, Ding JH, Du RH, Hu G (2021) Astrocytic Kir6.1 deletion aggravates neurodegeneration in the lipopolysaccharide-induced mouse model of Parkinson’s disease via astrocyte-neuron cross talk through complement C3-C3R signaling. Brain Behav Immun 95:310–320. 10.1016/j.bbi.2021.04.003 33838249

[132] Hu ZL, Sun T, Lu M, Ding JH, Du RH, Hu G (2019) Kir6.1/K-ATP channel on astrocytes protects against dopaminergic neurodegeneration in the MPTP mouse model of Parkinson’s disease via promoting mitophagy. Brain Behav Immun 81:509–522. 10.1016/j.bbi.2019.07.009 31288070

[133] Morales I, Sanchez A, Rodriguez-Sabate C, Rodriguez M (2017) Striatal astrocytes engulf dopaminergic debris in Parkinson’s disease: a study in an animal model. PLoS One 12. 10.1371/JOURNAL.PONE.0185989 PMC5640218 29028815

[134] Morales I, Sanchez A, Puertas-Avendaño R, Rodriguez-Sabate C, Perez-Barreto A, Rodriguez M (2020) Neuroglial transmitophagy and Parkinson’s disease. Glia 68:2277–2299. 10.1002/GLIA.23839 32415886

[135] Morales I, Puertas-Avendaño R, Sanchez A, Perez-Barreto A, Rodriguez-Sabate C, Rodriguez M (2021) Astrocytes and retrograde degeneration of nigrostriatal dopaminergic neurons in Parkinson’s disease: removing axonal debris. Transl Neurodegener 10:43. 10.1186/S40035-021-00262-1 34727977 PMC8562009

[136] Fiorino G, Di Florio DN, Hadley DH, Ross OA, Fiesel FC, Springer (W) PINK1 loss in astrocytes triggers inflammatory dysfunction and neuronal death. BioRxiv 2026.06.04.729996. 10.64898/2026.06.04.729996

[137] Bantle CM, Hirst WD, Weihofen A, Shlevkov E (2021) Mitochondrial dysfunction in astrocytes: a role in Parkinson’s disease? Front Cell Dev Biol 8:608026. 10.3389/FCELL.2020.608026 33537300 PMC7849831

[138] Zaltieri M, Longhena F, Pizzi M, Missale C, Spano P, Bellucci A (2015) Mitochondrial dysfunction and α-synuclein synaptic pathology in Parkinson’s disease: who’s on first? Parkinsons Dis. 10.1155/2015/108029 25918668 PMC4396726

[139] Lee JH, Han JH, Kim H, Park SM, Joe EH, Jou I (2019) Parkinson’s disease-associated LRRK2-G2019S mutant acts through regulation of SERCA activity to control ER stress in astrocytes. Acta Neuropathol Commun 7:68. 10.1186/S40478-019-0716-4 31046837 PMC6498585

[140] Liu M, Qin L, Wang L, Tan J, Zhang H, Tang J, Shen X, Tan L et al (2018) α-Synuclein induces apoptosis of astrocytes by causing dysfunction of the endoplasmic reticulum-Golgi compartment. Mol Med Rep 18:322. 10.3892/MMR.2018.9002 PMC6059687 29749529

[141] Liu M, Qin L, Wang L, Tan J, Zhang H, Tang J, Shen X, Tan L et al (2018) ?-Synuclein induces apoptosis of astrocytes by causing dysfunction of the endoplasmic reticulum-Golgi compartment. Mol Med Rep 18:322–332. 10.3892/MMR.2018.9002/ABSTRACT PMC6059687 29749529

[142] Meares GP, Liu Y, Rajbhandari R, Qin H, Nozell SE, Mobley JA, Corbett JA, Benveniste EN (2014) PERK-dependent activation of JAK1 and STAT3 contributes to endoplasmic reticulum stress-induced inflammation. Mol Cell Biol 34:3911. 10.1128/MCB.00980-14 25113558 PMC4187715

[143] Guthrie LN, Abiraman K, Plyler ES, Sprenkle NT, Gibson SA, McFarland BC, Rajbhandari R, Rowse AL et al (2016) Attenuation of PKR-like ER kinase (PERK) signaling selectively controls endoplasmic reticulum stress-induced inflammation without compromising immunological responses. J Biol Chem 291:15830–15840. 10.1074/jbc.M116.738021 PMC4957064 27226638

[144] Sprenkle NT, Lahiri A, Simpkins JW, Meares GP (2019) Endoplasmic reticulum stress is transmissible in vitro between cells of the central nervous system. J Neurochem 148:516–530. 10.1111/JNC.14642 30520047 PMC6379102

[145] Wang Y, Chen Y, Zhou Q, Xu J, Qian Q, Ni P, Qian Y (2018) Mild endoplasmic reticulum stress protects against lipopolysaccharide-induced astrocytic activation and blood-brain barrier hyperpermeability. Front Cell Neurosci 12:387415. 10.3389/FNCEL.2018.00222/BIBTEX PMC6077757 30104960

[146] Ledesma MD, Galvan C, Hellias B, Dotti C, Jensen PH (2002) Astrocytic but not neuronal increased expression and redistribution of parkin during unfolded protein stress. J Neurochem 83:1431–1440. 10.1046/J.1471-4159.2002.01253.X 12472897

[147] Singh K, Han K, Tilve S, Wu K, Geller HM, Sack MN (2018) Parkin targets NOD2 to regulate astrocyte endoplasmic reticulum stress and inflammation. Glia 66:2427. 10.1002/GLIA.23482 30378174 PMC6275110

[148] Shi CH, Tang BS, Wang L, Lv ZY, Wang J, Luo LZ, Shen L, Jiang H et al (2011) PLA2G6 gene mutation in autosomal recessive early-onset parkinsonism in a Chinese cohort. Neurology 77:75–81. 10.1212/WNL.0B013E318221ACD3 21700586

[149] Liu J, Tan J, Tang B, Guo J (2024) Unveiling the role of iPLA2β in neurodegeneration: from molecular mechanisms to advanced therapies. Pharmacol Res 202:107114. 10.1016/J.PHRS.2024.107114 38395207

[150] Nguyen D, Alavi MV, Kim KY, Kang T, Scott RT, Noh YH, Lindsey JD, Wissinger B et al (2011) A new vicious cycle involving glutamate excitotoxicity, oxidative stress and mitochondrial dynamics. Cell Death Dis. 10.1038/CDDIS.2011.117 PMC3252734 22158479

[151] Karki P, Smith K, Johnson J, Aschner M, Lee EY (2015) Genetic dys-regulation of astrocytic glutamate transporter EAAT2 and its implications in neurological disorders and manganese toxicity. Neurochem Res 40:380–388. 10.1007/S11064-014-1391-2 25064045 PMC4308576

[152] Centelles JJ (2016) Glutamate transporters: the regulatory proteins for excitatory/excitotoxic glutamate in brain. J Transl Sci 2. 10.15761/JTS.1000123

[153] Almohmadi NH, Al-kuraishy HM, Al-Gareeb AI, Albuhadily AK, Abdelaziz AM, Jabir MS, Alexiou A, Papadakis M et al (2025) Glutamatergic dysfunction in neurodegenerative diseases focusing on Parkinson’s disease: role of glutamate modulators. Brain Res Bull 225:111349. 10.1016/J.BRAINRESBULL.2025.111349 40252703

[154] Iovino L, Tremblay ME, Civiero L (2020) Glutamate-induced excitotoxicity in Parkinson’s disease: the role of glial cells. J Pharmacol Sci 144:151–164. 10.1016/J.JPHS.2020.07.011 32807662

[155] Chalifoux JR, Carter AG (2011) Glutamate spillover promotes the generation of NMDA spikes. J Neurosci 31:16435–16446. 10.1523/JNEUROSCI.2777-11.2011 22072693 PMC3235338

[156] Wang J, Zhao J, Zhao K, Wu S, Chen X, Hu W (2024) The role of calcium and iron homeostasis in Parkinson’s disease. Brain Sciences 14:88. 10.3390/BRAINSCI14010088 PMC10813814 38248303

[157] Verma M, Lizama BN, Chu CT (2022) Excitotoxicity, calcium and mitochondria: a triad in synaptic neurodegeneration. Transl Neurodegener 11(1):1–14. 10.1186/S40035-021-00278-7 PMC8788129 35078537

[158] Chotibut T, Davis RW, Arnold JC, Frenchek Z, Gurwara S, Bondada V, Geddes JW, Salvatore MF (2014) Ceftriaxone increases glutamate uptake and reduces striatal tyrosine hydroxylase loss in 6-OHDA Parkinson’s model. Mol Neurobiol 49:1282–1292. 10.1007/S12035-013-8598-0 24297323 PMC4618839

[159] Wang WW, Zhang XR, Zhang ZR, Wang XS, Chen J, Chen SY, Xie CL (2018) Effects of mGluR5 antagonists on Parkinson’s patients with L-Dopa-induced dyskinesia: a systematic review and meta-analysis of randomized controlled trials. Front Aging Neurosci 10:312628. 10.3389/FNAGI.2018.00262/BIBTEX PMC6142875 30271338

[160] Rylander D, Iderberg H, Li Q, Dekundy A, Zhang J, Li H, Baishen R, Danysz W et al (2010) A mGluR5 antagonist under clinical development improves L-DOPA-induced dyskinesia in parkinsonian rats and monkeys. Neurobiol Dis 39:352–361. 10.1016/j.nbd.2010.05.001 20452425

[161] Hudson J, Granholm AC, Gerhardt GA, Henry MA, Hoffman A, Biddle P, Leela NS, Mackerlova L et al (1995) Glial cell line-derived neurotrophic factor augments midbrain dopaminergic circuits in vivo. Brain Res Bull 36:425–432. 10.1016/0361-9230(94)00224-O 7712205

[162] Carballo-Molina OA, Kolberg-Edelbrock AN, Alvarez-Saavedra M, Álvarez Z, Fyrner T, Perez-Rosello T, Syrgiannis Z, Chin SM et al (2025) Supramolecular nanostructure mimics GDNF trophic effects in vitro on human dopaminergic neurons. Npj Regen Med 10(1):37. 10.1038/s41536-025-00424-z PMC12334742 40781074

[163] Luo J (2022) TGF-β as a key modulator of astrocyte reactivity: disease relevance and therapeutic implications. Biomedicines 10:1206. 10.3390/BIOMEDICINES10051206 35625943 PMC9138510

[164] L’Episcopo F, Tirolo C, Testa N, Caniglia S, Morale MC, Cossetti C, D’Adamo P, Zardini E et al (2011) Reactive astrocytes and Wnt/β-catenin signaling link nigrostriatal injury to repair in 1-methyl-4-phenyl-1,2,3,6-tetrahydropyridine model of Parkinson’s disease. Neurobiol Dis 41:508–527. 10.1016/J.NBD.2010.10.023 PMC3558878 21056667

[165] Rizor A, Pajarillo E, Johnson J, Aschner M, Lee E (2019) Astrocytic oxidative/nitrosative stress contributes to Parkinson’s disease pathogenesis: the dual role of reactive astrocytes. Antioxidants (Basel) 8:265. 10.3390/ANTIOX8080265 31374936 PMC6719180

[166] Zhang Z, Zhang N, Ding S (2025) Reactive astrocytes release GDNF to promote brain recovery and neuronal survival following ischemic stroke. Neurochem Res 50:117. 10.1007/S11064-025-04370-6 40085335 PMC11909085

[167] Albini M, Krawczun-Rygmaczewska A, Cesca F (2023) Astrocytes and brain-derived neurotrophic factor (BDNF). Neurosci Res 197:42–51 36780947 10.1016/j.neures.2023.02.001

[168] Ozoran H, Srinivasan R (2023) Astrocytes and alpha-synuclein: friend or foe? J Parkinsons Dis 13:1289. 10.3233/JPD-230284 38007674 PMC10741342

[169] Toledo-Aral JJ, Méndez-Ferrer S, Pardal R, Echevarría M, López-Barneo J (2003) Trophic restoration of the nigrostriatal dopaminergic pathway in long-term carotid body-grafted parkinsonian rats. J Neurosci 23:141–148. 10.1523/JNEUROSCI.23-01-00141.2003 12514210 PMC6742142

[170] Patel NK, Bunnage M, Plaha P, Svendsen CN, Heywood P, Gill SS (2005) Intraputamenal infusion of glial cell line-derived neurotrophic factor in PD: a two-year outcome study. Ann Neurol 57:298–302. 10.1002/ANA.20374 15668979

[171] Whone A, Luz M, Boca M, Woolley M, Mooney L, Dharia S, Broadfoot J, Cronin D et al (2019) Randomized trial of intermittent intraputamenal glial cell line-derived neurotrophic factor in Parkinson’s disease. Brain 142:512. 10.1093/BRAIN/AWZ023 30808022 PMC6391602

[172] Kambey PA, Kanwore K, Ayanlaja AA, Nadeem I, Du YZ, Buberwa W, Liu WY, Gao D (2021) Failure of glial cell-line derived neurotrophic factor (GDNF) in clinical trials orchestrated by reduced NR4A2 (NURR1) transcription factor in Parkinson’s disease. A systematic review Front Aging Neurosci 13:645583. 10.3389/FNAGI.2021.645583 PMC7943926 33716718

[173] Van Laar AD, Christine CW, Phielipp N, Larson PS, Elder JB, Merola A, San Sebastian W, Fiandaca MS et al (2025) Intraputaminal delivery of adeno-associated virus serotype 2-glial cell line-derived neurotrophic factor in mild or moderate Parkinson’s disease. Mov Disord 40:1297–1306. 10.1002/MDS.30193 PMC12273615 40395017

[174] Heiss JD, Ray-Chaudhury A, Kleiner DE, Ehrlich DJ, Scott G, Edwards NA, Goldstein DS, Hammoud DA et al (2024) Persistent GDNF expression 45 months after putaminal infusion of AAV2-GDNF in a patient with Parkinson’s disease. Mov Disord 39:1412. 10.1002/MDS.29820 PMC11341257 38718138

[175] Kim SC, Lee SY, Jung UJ, Kim SR (2025) Application of neurotrophic factors as a therapeutic approach for neurodegenerative diseases. Exp Neurobiol 34:169–199. 10.5607/EN25023 41029914 PMC12580398

[176] Garbayo E, Montero-Menei CN, Ansorena E, Lanciego JL, Aymerich MS, Blanco-Prieto MJ (2009) Effective GDNF brain delivery using microspheres-a promising strategy for Parkinson’s disease. J Control Release 135:119–126. 10.1016/J.JCONREL.2008.12.010 19154763

[177] Bonvento G, Bolaños JP (2021) Astrocyte-neuron metabolic cooperation shapes brain activity. Cell Metab 33:1546–1564. 10.1016/J.CMET.2021.07.006 34348099

[178] Suzuki A, Stern SA, Bozdagi O, Huntley GW, Walker RH, Magistretti PJ, Alberini CM (2011) Astrocyte-neuron lactate transport is required for long-term memory formation. Cell 144:810–823. 10.1016/J.CELL.2011.02.018 21376239 PMC3073831

[179] Pierre K, Pellerin L (2005) Monocarboxylate transporters in the central nervous system: distribution, regulation and function. J Neurochem 94:1–14. 10.1111/J.1471-4159.2005.03168.X;PAGEGROUP:STRING:PUBLICATION 15953344

[180] Zorov DB, Schurr A (2025) The feud over lactate and its role in brain energy metabolism: an unnecessary burden on research and the scientists who practice it. Int J Mol Sci 26:4429. 10.3390/IJMS26094429 PMC12072709 40362665

[181] Sonninen TM, Hämäläinen RH, Koskuvi M, Oksanen M, Shakirzyanova A, Wojciechowski S, Puttonen K, Naumenko N, Goldsteins G, Laham-Karam N, Lehtonen M, Tavi P, Koistinaho J, Lehtonen Š (2020) Metabolic alterations in Parkinson’s disease astrocytes. Sci Rep 10(1):14474. 10.1038/s41598-020-71329-8 PMC7468111 32879386

[182] Bak LK, Walls AB (2018) Astrocytic glycogen metabolism in the healthy and diseased brain. J Biol Chem 293:7108–7116. 10.1074/JBC.R117.803239 29572349 PMC5950001

[183] Mason S (2017) Lactate shuttles in neuroenergetics—homeostasis, allostasis and beyond. Front Neurosci 11:43. 10.3389/FNINS.2017.00043 28210209 PMC5288365

[184] Poff AM, Moss S, Soliven M, D’Agostino DP (2021) Ketone supplementation: meeting the needs of the brain in an energy crisis. Front Nutr 8:783659. 10.3389/FNUT.2021.783659 35004814 PMC8734638

[185] Cavaleri F, Bashar E (2018) Potential synergies of β-hydroxybutyrate and butyrate on the modulation of metabolism, inflammation, cognition, and general health. J Nutr Metab. 10.1155/2018/7195760 29805804 PMC5902005

[186] Babenko VA, Varlamova EG, Saidova AA, Turovsky EA, Plotnikov EY (2024) Lactate protects neurons and astrocytes against ischemic injury by modulating Ca2+ homeostasis and inflammatory response. FEBS J 291:1684–1698. 10.1111/FEBS.17051 38226425

[187] Islam S, Noorani A, Sun Y, Michikawa M, Zou K (2025) Multi-functional role of apolipoprotein E in neurodegenerative diseases. Front Aging Neurosci 17:1535280. 10.3389/FNAGI.2025.1535280/FULL 39944166 PMC11813892

[188] Wang Y, Wang B, Hou J, Huo X, Liu C, Guan R, Chen H, Zhou Y et al (2026) Lipid droplets in astrocytes: key organelles for CNS homeostasis and disease (review). Int J Mol Med 57:1–17. 10.3892/IJMM.2025.5691/HTML PMC12634068 41235649

[189] Zhong J, Peng Y, Zhang L, Xiao B, Zhang M (2025) Astrocyte lipid droplet dynamics orchestrate neurological disorders and therapeutic horizons. Small Sci 5:2500152. 10.1002/SMSC.202500152 40917390 PMC12412622

[190] Halim DO, Di Biase E, Rajon A, Jordi L, Hallett PJ, Isacson O (2025) APOE3 astrocytes can rescue lipid abnormalities and dystrophic neurites of APOE4 human neurons. BioRxiv 2025.10.24.684364. 10.1101/2025.10.24.684364 PMC13003211 41867897

[191] Brekk OR, Honey JR, Lee S, Hallett PJ, Isacson O (2020) Cell type-specific lipid storage changes in Parkinson’s disease patient brains are recapitulated by experimental glycolipid disturbance. Proc Natl Acad Sci 117:27646–27654. 10.1073/PNAS.2003021117 33060302 PMC7959493

[192] Galvagnion C (2017) The role of lipids interacting with α-synuclein in the pathogenesis of Parkinson’s disease. J Parkinsons Dis 7:433–450. 10.3233/JPD-171103 28671142

[193] Dear AJ, Teng X, Ball SR, Lewin J, Horne RI, Clow D, Stevenson A, Harper N et al (2024) Molecular mechanism of α-synuclein aggregation on lipid membranes revealed. Chem Sci 15:7229–7242. 10.1039/D3SC05661A PMC11095391 38756798

[194] Loix M, Wouters E, Vanherle S, Dehairs J, McManaman JL, Kemps H, Swinnen JV, Haidar M et al (2022) Perilipin-2 limits remyelination by preventing lipid droplet degradation. Cell Mol Life Sci 79:515. 10.1007/S00018-022-04547-0 36100764 PMC11803036

[195] Tao J, Berthet A, Citron YR, Tsiolaki PL, Stanley R, Gestwicki JE, Agard DA, McConlogue L (2021) Hsp70 chaperone blocks α-synuclein oligomer formation via a novel engagement mechanism. J Biol Chem 296:100613. 10.1016/J.JBC.2021.100613 33798554 PMC8102405

[196] Idi W, Sheta R, Oueslati A (2025) Interplay between lipid droplets and alpha-synuclein: implication in Parkinson’s disease pathogenesis. Front Mol Neurosci 18:1681039. 10.3389/FNMOL.2025.1681039/FULL 41404624 PMC12703102

[197] Watson DC, Bayik D, Storevik S, Moreino SS, Sprowls SA, Han J, Augustsson MT, Lauko A et al (2023) GAP43-dependent mitochondria transfer from astrocytes enhances glioblastoma tumorigenicity. Nat Cancer 4(5):648–664. 10.1038/s43018-023-00556-5 PMC10212766 37169842

[198] Chakraborty R, Nonaka T, Hasegawa M, Zurzolo C (2023) Tunnelling nanotubes between neuronal and microglial cells allow bi-directional transfer of α-Synuclein and mitochondria. Cell Death Dis 14(5):329. 10.1038/s41419-023-05835-8 37202391 PMC10195781

[199] Walecha V, Luthra PM (2025) The mitochondrial-astrocyte-neuron triad hypothesis in Parkinson’s disease: a toxic feedback loop of metabolism, aggregation, and oxidative stress. Neurochem Res 50:317. 10.1007/S11064-025-04559-9 41045318

[200] Todkar K, Chikhi L, Desjardins V, El-Mortada F, Pépin G, Germain M (2021) Selective packaging of mitochondrial proteins into extracellular vesicles prevents the release of mitochondrial DAMPs. Nature Commun 12(1):1971. 10.1038/s41467-021-21984-w PMC8009912 33785738

[201] Gervasi A, D’Aprile S, Denaro S, Amorini MA, Vicario N, Parenti R (2025) Connexin 43 role in mitochondrial transfer and homeostasis in the central nervous system. J Cell Physiol 240:e70086. 10.1002/JCP.70086 40838510 PMC12368938

[202] Irwin RM, Thomas MA, Fahey MJ, Mayán MD, Smyth JW, Delco ML (2024) Connexin 43 regulates intercellular mitochondrial transfer from human mesenchymal stromal cells to chondrocytes. Stem Cell Res Ther 15(1):359. 10.1186/S13287-024-03932-9 39390589 PMC11468299

[203] Fujii Y, Maekawa S, Morita M (2017) Astrocyte calcium waves propagate proximally by gap junction and distally by extracellular diffusion of ATP released from volume-regulated anion channels. Sci Rep 7(1):713115. 10.1038/s41598-017-13243-0 PMC5640625 29030562

[204] Cartes-Saavedra B, Ghosh A, Hajnóczky G (2025) The roles of mitochondria in global and local intracellular calcium signalling. Nat Rev Mol Cell Biol 26:456–475. 10.1038/S41580-024-00820-1 39870977

[205] Lock JT, Parker I, Smith IF (2016) Communication of Ca2+ signals via tunneling membrane nanotubes is mediated by transmission of inositol trisphosphate through gap junctions. Cell Calcium 60:266–272. 10.1016/J.CECA.2016.06.004 27388952 PMC5035603

[206] Rajasekaran S, Witt SN (2021) Trojan horses and tunneling nanotubes enable α-synuclein pathology to spread in Parkinson disease. PLoS Biol. 10.1371/JOURNAL.PBIO.3001331 34284485 PMC8291968

[207] Díaz EF, Labra VC, Alvear TF, Mellado LA, Inostroza CA, Oyarzún JE, Salgado N, Quintanilla RA et al (2019) Connexin 43 hemichannels and pannexin-1 channels contribute to the α-synuclein-induced dysfunction and death of astrocytes. Glia 67:1598–1619. 10.1002/GLIA.23631 31033038

[208] Palese F, Rakotobe M, Zurzolo C (2025) Transforming the concept of connectivity: unveiling tunneling nanotube biology and their roles in brain development and neurodegeneration. 105:1823–1865. 10.1152/Physrev.00023.2024 40067081

[209] Chiareli RA, Carvalho GA, Marques BL, Mota LS, Oliveira-Lima OC, Gomes RM, Birbrair A, Gomez RS et al (2021) The role of astrocytes in the neurorepair process. Front Cell Dev Biol 9:665795. 10.3389/FCELL.2021.665795 PMC8186445 34113618

[210] Droguerre M, Tsurugizawa T, Duchêne A, Portal B, Guiard BP, Déglon N, Rouach N, Hamon M, Mouthon F, Ciobanu L, Charvériat M (2019) A new tool for in vivo study of astrocyte connexin 43 in brain. Sci Rep 9(1):18292. 10.1038/s41598-019-54858-9 PMC6892890 31797899

[211] Li SJ, Zheng QW, Zheng J, Zhang JB, Liu H, Tie JJ, Zhang KL, Wu FF et al (2025) Mitochondria transplantation transiently rescues cerebellar neurodegeneration improving mitochondrial function and reducing mitophagy in mice. Nat Commun 16(1):2839. 10.1038/s41467-025-58189-4 PMC11929859 40121210

[212] Wang X, Gerdes HH (2015) Transfer of mitochondria via tunneling nanotubes rescues apoptotic PC12 cells. Cell Death Differ 22:1181–1191. 10.1038/CDD.2014.211 25571977 PMC4572865

[213] Selim MS, Matani BR, Henry-Ojo HO, Narayanan SP, Somanath PR (2025) Claudin 5 across the vascular landscape: from blood–tissue barrier regulation to disease mechanisms. Cells 14:1346. 10.3390/CELLS14171346 40940757 PMC12427713

[214] Jiao H, Wang Z, Liu Y, Wang P, Xue Y (2011) Specific role of tight junction proteins claudin-5, occludin, and ZO-1 of the blood-brain barrier in a focal cerebral ischemic insult. J Mol Neurosci 44:130–139. 10.1007/S12031-011-9496-4 21318404

[215] Garcia-Martínez T, Gornatti DG, Ortiz M, Cañellas G, Heine-Suñer D, Vives-Bauzà C (2025) The triad of blood–brain barrier integrity: endothelial cells, astrocytes, and pericytes in perinatal stroke pathophysiology. Int J Mol Sci 26:1886. 10.3390/IJMS26051886 PMC11900453 40076511

[216] Zhang Y, Zhang Y, Song C, Wu Y, Cui K, Zhang X, Sun Y, Shen H et al (2025) Astrocyte-derived MMP-9 is a key mediator of pseudorabies virus penetration of the blood–brain barrier and tight junction disruption. Vet Res 56:72. 10.1186/S13567-025-01486-Z PMC11963458 40176142

[217] Deng Z, Zhou L, Wang Y, Liao S, Huang Y, Shan Y, Tan S, Zeng Q, Peng L, Huang H, Lu Z (2020) Astrocyte-derived VEGF increases cerebral microvascular permeability under high salt conditions. Aging (Albany NY) 12:11781. 10.18632/AGING.103348 PMC7343440 32568100

[218] Valenzuela-Arzeta IE, Soto-Rojas LO, Flores-Martinez YM, Delgado-Minjares KM, Gatica-Garcia B, Mascotte-Cruz JU, Nava P, Aparicio-Trejo OE et al (2023) LPS triggers acute neuroinflammation and parkinsonism involving NLRP3 inflammasome pathway and mitochondrial CI dysfunction in the rat. Int J Mol Sci 24:4628. 10.3390/IJMS24054628/S1 PMC10003606 36902058

[219] He JY, Di Li D, Wen Q, Qin TY, Long H, Bin Zhang S, Zhang F (2023) Synergistic effects of lipopolysaccharide and rotenone on dopamine neuronal damage in rats. CNS Neurosci Ther 29:2281–2291. 10.1111/CNS.14180 PMC10352892 36942519

[220] Zhao J, Bi W, Xiao S, Lan X, Cheng X, Zhang J, Lu D, Wei W, Wang Y, Li H, Fu Y, Zhu L (2019) Neuroinflammation induced by lipopolysaccharide causes cognitive impairment in mice. Sci Rep 9(1):5790. 10.1038/s41598-019-42286-8 PMC6453933 30962497

[221] Tian J, Dai SB, Jiang SS, Yang WY, Yan YQ, Lin ZH, Dong JX, Liu Y, Zheng R, Chen Y, Zhang BR, Pu JL (2022) Specific immune status in Parkinson’s disease at different ages of onset. Npj Parkinson’s Disease 8(1):5. 10.1038/s41531-021-00271-x PMC8748464 35013369

[222] Pacheco R, Chuen R, Chang C (2021) T-cell based immunotherapies for Parkinsons disease, Open Exploration 1(2):72–85. 10.37349/ENT.2021.00007

[223] Williams GP, Freuchet A, Michaelis T, Frazier A, Tran NK, Lima-Junior JR, Phillips EJ, Mallal SA, Litvan I, Goldman JG, Alcalay RN, Sidney J, Sulzer D, Sette A, Lindestam Arlehamn CS, Arlehamn CL (2025) PINK1 is a target of T cell responses in Parkinson’s disease. J Clin Invest 135. 10.1172/JCI180478 PMC11827839 39688918

[224] Gate D, Tapp E, Leventhal O, Shahid M, Nonninger TJ, Yang AC, Strempfl K, Unger MS, et al. (1979) CD4+ T cells contribute to neurodegeneration in Lewy body dementia. Science 374(2021):868–874. 10.1126/SCIENCE.ABF7266;WEBSITE:WEBSITE:AAAS-SITE;JOURNAL:JOURNAL:SCIENCE;WGROUP:STRING:PUBLICATION PMC9122025 34648304

[225] Bauer AT, Bürgers HF, Rabie T, Marti HH (2009) Matrix metalloproteinase-9 mediates hypoxia-induced vascular leakage in the brain via tight junction rearrangement. J Cereb Blood Flow Metab 30:837. 10.1038/JCBFM.2009.248 19997118 PMC2949161

[226] Lan G, Wang P, Chan RB, Liu Z, Yu Z, Liu X, Yang Y, Zhang J (2022) Astrocytic VEGFA: an essential mediator in blood-brain-barrier disruption in Parkinson’s disease. Glia 70:337–353. 10.1002/GLIA.24109 34713920

[227] Loh JS, Mak WQ, Tan LKS, Ng CX, Chan HH, Yeow SH, Foo JB, Ong YS, How CW, Khaw KY (2024) Microbiota–gut–brain axis and its therapeutic applications in neurodegenerative diseases. Signal Transduct Target Ther 9(1):37. 10.1038/s41392-024-01743-1 PMC10869798 38360862

[228] Radford-Smith DE, Oke K, Costa CFFA, Anthony DC (2025) Systematic review and meta-analysis of microbiota-gut-astrocyte axis perturbation in neurodegeneration, brain injury, and mood disorders. Brain Behav Immun Health 46:101013. 10.1016/J.BBIH.2025.101013 40485663 PMC12145769

[229] Almohmadi NH, Al-kuraishy HM, Hussein KS, Turkistani AM, Yousef FM, Al-Gareeb AI, Abdelaziz AM, Elewa YHA et al (2026) The gut-brain axis: a critical link between type 2 diabetes and Parkinson’s disease. Probiotics Antimicrob Proteins 2026:1–25. 10.1007/S12602-026-11014-W 41981378

[230] Bonaz B (2024) The gut-brain axis in Parkinson’s disease. Rev Neurol (Paris) 180:65–78. 10.1016/j.neurol.2023.11.004 38129277

[231] Chen M, Mor DE (2023) Gut-to-brain α-synuclein transmission in Parkinson’s disease: Evidence for prion-like mechanisms. Int J Mol Sci 24:7205. 10.3390/ijms24087205 37108366 PMC10139032

[232] Zhou Z, Xu N, Matei N, McBride DW, Ding Y, Liang H, Tang J, Zhang JH (2021) Sodium butyrate attenuated neuronal apoptosis via GPR41/Gβγ/PI3K/Akt pathway after MCAO in rats. J Cereb Blood Flow Metab 41:267–281. 10.1177/0271678X20910533 32151222 PMC8370004

[233] Skalny AV, Aschner M, Gritsenko VA, Martins AC, Tizabi Y, Korobeinikova TV, Paoliello MMB, Tinkov AA (2024) Modulation of gut microbiota with probiotics as a strategy to counteract endogenous and exogenous neurotoxicity. Adv Neurotoxicol 11:133–176. 10.1016/BS.ANT.2024.02.002 38741946 PMC11090489

[234] Shekar S, Venkatachalapathy R, Jayaraman A, Sai Supra Siddhu N (2025) Fecal microbiota transplantation for Parkinson’s disease: a systematic review of clinical evidence. Med Microecol 25:100128. 10.1016/J.MEDMIC.2025.100128

[235] Nishiwaki H, Ito M, Hamaguchi T, Maeda T, Kashihara K, Tsuboi Y, Ueyama J, Yoshida T, Hanada H, Takeuchi I, Katsuno M, Hirayama M, Ohno K (2022) Short chain fatty acids-producing and mucin-degrading intestinal bacteria predict the progression of early Parkinson’s disease. Npj Parkinson’s Disease 8(1):65. 10.1038/s41531-022-00328-5 PMC9160257 35650236

[236] Pang S, Ren Z, Ding H, Chan P (2025) Short-chain fatty acids mediate enteric and central nervous system homeostasis in Parkinson’s disease: innovative therapies and their translation. Neural Regen Res 21:938. 10.4103/NRR.NRR-D-24-01265 40313087 PMC12296502

[237] Owe-Larsson M, Drobek D, Iwaniak P, Kloc R, Urbanska EM, Chwil M (2025) Microbiota-derived tryptophan metabolite indole-3-propionic acid-emerging role in neuroprotection. Molecules 30:3628. 10.3390/MOLECULES30173628 40942152 PMC12429930

[238] Barroso A, Mahler JV, Fonseca-Castro PH, Quintana FJ (2021) The aryl hydrocarbon receptor and the gut–brain axis. Cell Mol Immunol 18:259–268. 10.1038/S41423-020-00585-5;SUBJMETA 33408340 PMC8027889

[239] Valenzuela-Arzeta IE, Soto-Rojas LO, Flores-Martinez YM, Delgado-Minjares KM, Gatica-Garcia B, Mascotte-Cruz JU, Nava P, Aparicio-Trejo OE et al (2023) LPS triggers acute neuroinflammation and parkinsonism involving NLRP3 inflammasome pathway and mitochondrial CI dysfunction in the rat. Int J Mol Sci. 10.3390/ijms24054628 PMC10003606 36902058

[240] Di Vincenzo F, Del Gaudio A, Petito V, Lopetuso LR, Scaldaferri F (2023) Gut microbiota, intestinal permeability, and systemic inflammation: a narrative review. Intern Emerg Med 19:275. 10.1007/S11739-023-03374-W 37505311 PMC10954893

[241] Jones ML, Martoni CJ, Ganopolsky JG, Labbé A, Prakash S (2014) The human microbiome and bile acid metabolism: dysbiosis, dysmetabolism, disease and intervention. Expert Opin Biol Ther 14:467–482. 10.1517/14712598.2014.880420 24479734

[242] Duan WX, Wang F, Liu JY, Liu CF (2023) Relationship between short-chain fatty acids and Parkinson’s disease: a review from pathology to clinic. Neurosci Bull 40:500. 10.1007/S12264-023-01123-9 37755674 PMC11003953

[243] O’Riordan KJ, Collins MK, Moloney GM, Knox EG, Aburto MR, Fülling C, Morley SJ, Clarke G et al (2022) Short chain fatty acids: microbial metabolites for gut-brain axis signalling. Mol Cell Endocrinol 546:111572. 10.1016/J.MCE.2022.111572 35066114

[244] Missiego-Beltrán J, Olalla-Álvarez EM, González-Brugera A, Beltrán-Velasco AI (2024) Implications of butyrate signaling pathways on the motor symptomatology of Parkinson’s disease and neuroprotective effects—therapeutic approaches: a systematic review. Int J Mol Sci 25:8998. 10.3390/IJMS25168998 39201684 PMC11354563

[245] Saikachain N, Sungkaworn T, Muanprasat C, Asavapanumas N (2023) Neuroprotective effect of short-chain fatty acids against oxidative stress-induced SH-SY5Y injury via GPR43-dependent pathway. J Neurochem 166:201–214. 10.1111/JNC.15827;WGROUP:STRING:PUBLICATION 37070532

[246] Iwaniak P, Owe-Larsson M, Urbańska EM (2024) Microbiota, tryptophan and aryl hydrocarbon receptors as the target triad in Parkinson’s disease-a narrative review. Int J Mol Sci. 10.3390/ijms25052915 38474162 PMC10931713

[247] Yanguas-Casás N, Barreda-Manso MA, Nieto-Sampedro M, Romero-Ramírez L (2017) TUDCA: an agonist of the bile acid receptor GPBAR1/TGR5 with anti-inflammatory effects in microglial cells. J Cell Physiol 232:2231–2245. 10.1002/JCP.25742 27987324

[248] Li L, Acioglu C, Heary RF, Elkabes S (2020) Role of astroglial toll-like receptors (TLRs) in central nervous system infections, injury and neurodegenerative diseases. Brain Behav Immun 91:740. 10.1016/J.BBI.2020.10.007 33039660 PMC7543714

[249] Furnari S, Ciantia R, Garozzo A, Furneri PM, Fuochi V (2025) Lactobacilli-derived microbe-associated molecular patterns (MAMPs) in host immune modulation. Biomolecules 15:1609. 10.3390/BIOM15111609/S1 41301527 PMC12650587

[250] Hastings MH, Brancaccio M, Gonzalez-Aponte MF, Herzog ED (2023) Circadian rhythms and astrocytes: the good, the bad, and the ugly. Annu Rev Neurosci 46:123. 10.1146/ANNUREV-NEURO-100322-112249 36854316 PMC10381027

[251] Xu K, Zhang Y, Shi Y, Zhang Y, Zhang C, Wang T, Lv P, Bai Y et al (2024) Circadian rhythm disruption: a potential trigger in Parkinson’s disease pathogenesis. Front Cell Neurosci 18:1464595. 10.3389/FNCEL.2024.1464595 PMC11557417 39539340

[252] Tso CF, Simon T, Greenlaw AC, Puri T, Mieda M, Herzog ED (2017) Astrocytes regulate daily rhythms in the suprachiasmatic nucleus and behavior. Curr Biol 27:1055–1061. 10.1016/J.CUB.2017.02.037 28343966 PMC5380592

[253] Ness N, Díaz-Clavero S, Hoekstra MMB, Brancaccio M (2024) Rhythmic astrocytic GABA production synchronizes neuronal circadian timekeeping in the suprachiasmatic nucleus. EMBO J 44:356–381. 10.1038/S44318-024-00324-W 39623138 PMC11731042

[254] Vieira E, Mirizio GG, Barin GR, de Andrade RV, Nimer NFS, La Sala L (2020) Clock genes, inflammation and the immune system—implications for diabetes, obesity and neurodegenerative diseases. Int J Mol Sci 21:9743. 10.3390/IJMS21249743 33371208 PMC7766955

[255] Barca-Mayo O, Pons-Espinal M, Follert P, Armirotti A, Berdondini L, De Pietri Tonelli D (2017) Astrocyte deletion of Bmal1 alters daily locomotor activity and cognitive functions via GABA signalling. Nat Commun 8(1):14336. 10.1038/ncomms14336 28186121 PMC5309809

[256] Chen YC, Wang WS, Lewis SJG, Wu SL (2023) Fighting against the clock: circadian disruption and Parkinson’s disease. J Mov Disord 17:1. 10.14802/JMD.23216 PMC10846969 37989149

[257] Kim Y, Dube SE, Park CB (2025) Brain energy homeostasis: the evolution of the astrocyte-neuron lactate shuttle hypothesis, Korean. J Physiol Pharmacol 29:1–8. 10.4196/KJPP.24.388 PMC11694005 39725609

[258] Ozoran H, Srinivasan R (2023) Astrocytes and alpha-synuclein: friend or foe? J Parkinsons Dis 13:1289–1301. 10.3233/JPD-230284/ASSET/7983C896-A134-45BF-B0D9-0E1FADF89BB1/ASSETS/GRAPHIC/10.3233_JPD-230284-FIG2.JPG 38007674 PMC10741342

[259] McKee CA, Lananna BV, Musiek ES (2019) Circadian regulation of astrocyte function: implications for Alzheimer’s disease. Cell Mol Life Sci 77:1049. 10.1007/S00018-019-03314-Y 31578625 PMC7098845

[260] Cavadini G, Petrzilka S, Kohler P, Jud C, Tobler I, Birchler T, Fontana A (2007) TNF-α suppresses the expression of clock genes by interfering with E-box-mediated transcription. Proc Natl Acad Sci U S A 104:12843. 10.1073/PNAS.0701466104 17646651 PMC1937554

[261] Duhart JM, Leone MJ, Paladino N, Evans JA, Castanon-Cervantes O, Davidson AJ, Golombek DA (2013) Suprachiasmatic astrocytes modulate the circadian clock in response to TNF-α. J Immunol. 10.4049/jimmunol.1300450 24062487 PMC3811024

[262] Shearn CT, Anderson AL, Devereaux MW, El Kasmi KC, Orlicky DJ, Sokol RJ (2023) Expression of circadian regulatory genes is dysregulated by increased cytokine production in mice subjected to concomitant intestinal injury and parenteral nutrition. PLoS ONE 18:e0290385. 10.1371/JOURNAL.PONE.0290385 37647292 PMC10468060

[263] Shi G, Xie P, Qu Z, Zhang Z, Dong Z, An Y, Xing L, Liu Z et al (2016) Distinct roles of HDAC3 in the core circadian negative feedback loop are critical for clock function. Cell Rep 14:823–834. 10.1016/j.celrep.2015.12.076 26776516

[264] Mintz EM, Marvel CL, Gillespie CF, Price KM, Albers HE (1999) Activation of NMDA receptors in the suprachiasmatic nucleus produces light-like phase shifts of the circadian clock in vivo. J Neurosci 19:5124. 10.1523/JNEUROSCI.19-12-05124.1999 10366645 PMC6782653

[265] Genc S, Kurnaz IA, Ozilgen M (2011) Astrocyte - neuron lactate shuttle may boost more ATP supply to the neuron under hypoxic conditions - in silico study supported by in vitro expression data. BMC Syst Biol 5:162. 10.1186/1752-0509-5-162 21995951 PMC3202240

[266] Petit JM, Magistretti PJ (2016) Regulation of neuron-astrocyte metabolic coupling across the sleep-wake cycle. Neuroscience 323:135–156. 10.1016/j.neuroscience.2015.12.007 26704637

[267] Musiek E, Cirrito J, Colonna M, Diwan A, Lee J-M, Mckee CA, Louis S (2023) Circadian protein BMAL1 regulates astrocyte activation and protein degradation in health and disease

[268] Kim HY, Kim S, Akaydin AN, Kim S, Hyeon SJ, Lee J, Ryu H (2026) The rise of astrocytes: are they guardians or troublemakers of the brain disorder? Exp Mol Med 58(2):301–318. 10.1038/s12276-025-01627-6 41639424 PMC12992555

[269] Maciel-Barón LÁ, Morales-Rosales SL, Silva-Palacios A, Rodríguez-Barrera RH, García-Álvarez JA, Luna-López A, Pérez VI, Torres C et al (2018) The secretory phenotype of senescent astrocytes isolated from Wistar newborn rats changes with anti-inflammatory drugs, but does not have a short-term effect on neuronal mitochondrial potential. Biogerontology 19:415–433. 10.1007/S10522-018-9767-3 30097900

[270] Riessland M, Ximerakis M, Jarjour AA, Zhang B, Orr ME (2024) Therapeutic targeting of senescent cells in the CNS. Nat Rev Drug Discov 23:817. 10.1038/S41573-024-01033-Z 39349637 PMC11927922

[271] Zamanian JL, Xu L, Foo LC, Nouri N, Zhou L, Giffard RG, Barres BA (2012) Genomic analysis of reactive astrogliosis. J Neurosci 32:6391–6410. 10.1523/JNEUROSCI.6221-11.2012 22553043 PMC3480225

[272] Bronstein JM, Paul K, Yang W, Haas RH, Shults CW, Le T, Ritz B (2021) Movement disorders: 36(3). Mov Disord 36:1815–1820. 10.1002/mds.28151 PMC4439327 25757798

[273] Meissner WG, Remy P, Giordana C, Maltête D, Derkinderen P, Houéto J-L, Anheim M, Benatru I et al (2024) Trial of lixisenatide in early Parkinson’s disease. N Engl J Med 390:1176–1185. 10.1056/NEJMOA2312323 38598572

[274] Multicenter, randomized, placebo-controlled, double-blind, parallel-group proof-of-concept study of lixisenatide in patients with early Parkinson’s disease (PD): the LIXIPARK trial (NCT03439943) - MDS Abstracts (n.d.) https://www.mdsabstracts.org/abstract/multicenter-randomized-placebo-controlled-double-blind-parallel-group-proof-of-concept-study-of-lixisenatide-in-patients-with-early-parkinsons-disease-pd-the-lixipark-trial-nct0343994/. Accessed 4 Dec 2025

[275] Cummings JL, Atri A, Feldman HH, Hansson O, Sano M, Knop FK, Johannsen P, León T et al (2025) Evoke and evoke+: design of two large-scale, double-blind, placebo-controlled, phase 3 studies evaluating efficacy, safety, and tolerability of semaglutide in early-stage symptomatic Alzheimer’s disease. Alzheimers Res Ther 17:14. 10.1186/S13195-024-01666-7 PMC11708093 39780249

[276] Semaglutide Phase III PIONEER, SUSTAIN, and STEP Trial (n.d.) https://www.delveinsight.com/blog/semaglutide-phase-iii-pioneer-sustain-and-step-trial. Accessed 4 Dec 2025

[277] McGarry A, Rosanbalm S, Leinonen M, Olanow CW, To D, Bell A, Lee D, Chang J, Dubow J, Dhall R, Burdick D, Parashos S, Feuerstein J, Quinn J, Pahwa R, Afshari M, Ramirez-Zamora A, Chou K, Tarakad A, Luca C, Klos K, Bordelon Y, St Hiliare MH, Shprecher D, Lee S, Dawson TM, Roschke V, Kieburtz K (2024) Safety, tolerability, and efficacy of NLY01 in early untreated Parkinson’s disease: a randomised, double-blind, placebo-controlled trial. Lancet Neurol 23:37–45. 10.1016/S1474-4422(23)00378-2 38101901

[278] Jennings D, Huntwork-Rodriguez S, Vissers MFJM, Daryani VM, Diaz D, Goo MS, Chen JJ, Maciuca R et al (2023) LRRK2 inhibition by BIIB122 in healthy participants and patients with Parkinson’s disease. Mov Disord 38:386–398. 10.1002/MDS.29297 36807624

[279] Huber VJ, Igarashi H, Ueki S, Kwee IL, Nakada T (2018) Aquaporin-4 facilitator TGN-073 promotes interstitial fluid circulation within the blood-brain barrier: [17O]H2O JJVCPE MRI study. NeuroReport 29:697–703. 10.1097/WNR.0000000000000990 29481527 PMC5965936

[280] Alghanimy A, Martin C, Gallagher L, Holmes WM (2023) The effect of a novel AQP4 facilitator, TGN-073, on glymphatic transport captured by diffusion MRI and DCE-MRI. PLoS ONE 18:e0282955. 10.1371/JOURNAL.PONE.0282955 36920936 PMC10016657

[281] Garbarino VR, Palavicini JP, Melendez J, Barthelemy NR, He Y, Kautz TF, Lopez-Cruzan M, Mathews JJ, Xu P, Zhang B, Saliba A, Ragi N, Sharma K, Mason D, Johnson S, Hendrix S, Craft S, Petersen RC, Espindola-Netto JM, Xue A, Tchkonia T, Kirkland JL, Salardini A, Musi N, Bateman RJ, Gonzales MM, Orr ME (2025) Evaluation of exploratory fluid biomarkers from a phase 1 senolytic trial in mild Alzheimer’s disease. Neurotherapeutics 22. 10.1016/J.NEUROT.2025.E00591 PMC12418413 40274471

[282] Christine CW, Bankiewicz KS, Van Laar AD, Richardson RM, Ravina B, Kells AP, Boot B, Martin AJ et al (2019) Magnetic resonance imaging-guided phase 1 trial of putaminal AADC gene therapy for Parkinson’s disease. Ann Neurol 85:704–714. 10.1002/ANA.25450 PMC6593762 30802998

[283] Liu SY, Chan P, Stoessl AJ (2017) The underlying mechanism of prodromal PD: insights from the parasympathetic nervous system and the olfactory system. Transl Neurodegener 6(1):4. 10.1186/S40035-017-0074-8 28239455 PMC5319081

[284] Lapshina KV, Ekimova IV (2024) Aquaporin-4 and Parkinson’s disease. Int J Mol Sci 25:1672. 10.3390/IJMS25031672 38338949 PMC10855351

[285] Buccellato FR, D’Anca M, Serpente M, Arighi A, Galimberti D (2022) The role of glymphatic system in Alzheimer’s and Parkinson’s disease pathogenesis. Biomedicines 10:2261. 10.3390/biomedicines10092261 36140362 PMC9496080

[286] Filippini A, Salvi V, Dattilo V, Magri C, Castrezzati S, Veerhuis R, Bosisio D, Gennarelli M, et al. (2023) LRRK2 kinase inhibition attenuates astrocytic activation in response to amyloid β1-42 fibrils. Biomolecules 13:307. 10.3390/BIOM13020307 36830676 PMC9953366

[287] Yun SP, Kam TI, Panicker N, Kim S, Oh Y, Park JS, Kwon SH, Park YJ et al (2018) Block of A1 astrocyte conversion by microglia is neuroprotective in models of Parkinson’s disease. Nat Med 24:931–938. 10.1038/S41591-018-0051-5 PMC6039259 29892066

[288] Zhang L, Zhang L, Li Y, Li L, Melchiorsen JU, Rosenkilde M, Hölscher C (2020) The novel dual GLP-1/GIP receptor agonist DA-CH5 is superior to single GLP-1 receptor agonists in the MPTP model of Parkinson’s disease. J Parkinsons Dis 10:523–542. 10.3233/JPD-191768 31958096

[289] Hustad E, Aasly JO (2020) Clinical and imaging markers of prodromal Parkinson’s disease. Front Neurol 11:395. 10.3389/FNEUR.2020.00395 32457695 PMC7225301

[290] Jennings D, Siderowf A, Stern M, Seibyl J, Eberly S, Oakes D, Marek K (2014) Imaging prodromal Parkinson disease: the Parkinson Associated Risk Syndrome Study. Neurology 83:1739–1746. 10.1212/WNL.0000000000000960 25298306 PMC4239830

[291] Hinkle JT, Dawson VL, Dawson TM (2019) The A1 astrocyte paradigm: new avenues for pharmacological intervention in neurodegeneration. Mov Disord 34:959–969. 10.1002/MDS.27718 31136698 PMC6642014

[292] Lv MJ, Xue GF, Cheng HF, Meng PF, Lian X, Hölscher C, Li DF (2021) The GLP-1/GIP dual-receptor agonist DA5-CH inhibits the NF-κB inflammatory pathway in the MPTP mouse model of Parkinson’s disease more effectively than the GLP-1 single-receptor agonist NLY01. Brain Behav 11. 10.1002/BRB3.2231 PMC8413783 34125470

[293] Islam MT, Tuday E, Allen S, Kim J, Trott DW, Holland WL, Donato AJ, Lesniewski LA (2023) Senolytic drugs, dasatinib and quercetin, attenuate adipose tissue inflammation, and ameliorate metabolic function in old age. Aging Cell 22:e13767. 10.1111/ACEL.13767 36637079 PMC9924942

[294] Stoll AC, Harms AS (2026) Interconnected roles of astrocytes and the blood–brain barrier in Parkinson’s disease: pathological evidence, mechanistic insights, and knowledge gaps. Front Aging Neurosci 18:1765195. 10.3389/FNAGI.2026.1765195/FULL 41868432 PMC12999931

[295] Preininger MK, Kaufer D (2022) Blood–brain barrier dysfunction and astrocyte senescence as reciprocal drivers of neuropathology in aging. Int J Mol Sci 23:6217. 10.3390/IJMS23116217 35682895 PMC9180977

[296] Williams L, Qiu J, Waller S, Tsui D, Griffith J, Fung VS (2022) Challenges in managing late-stage Parkinson’s disease practical approaches and pitfalls. Aust J Gen Pract 51:778–785. 10.31128/AJGP-05-22-6438 36184862

[297] Van Laar A, Christine C, Merola A, Phielipp N, Elder B, Larson P, Sebastian WS, Fiandaca M et al (2024) Phase 1b safety and preliminary efficacy of bilateral intraputaminal delivery of AAV2 GDNF (AB-1005) in participants with mild or moderate Parkinson’s disease (N2.001). Neurology 102:6786. 10.1212/WNL.0000000000206690

[298] Fairley LH, Grimm A, Eckert A (2022) Mitochondria transfer in brain injury and disease. Cells 11:3603. 10.3390/CELLS11223603 36429030 PMC9688459

[299] Fogel DB (2018) Factors associated with clinical trials that fail and opportunities for improving the likelihood of success: a review. Contemp Clin Trials Commun 11:156. 10.1016/J.CONCTC.2018.08.001 30112460 PMC6092479

[300] Vaswani PA, Tropea TF, Dahodwala N (2020) Overcoming barriers to Parkinson disease trial participation: increasing diversity and novel designs for recruitment and retention. Neurotherapeutics 17:1724. 10.1007/S13311-020-00960-0 33150545 PMC7851248

[301] Athauda D, Foltynie T (2016) The glucagon-like peptide 1 (GLP) receptor as a therapeutic target in Parkinson’s disease: mechanisms of action. Drug Discov Today 21:802–818. 10.1016/j.drudis.2016.01.013 26851597

[302] Hölscher C (2020) Brain insulin resistance: role in neurodegenerative disease and potential for targeting. Expert Opin Investig Drugs 29:333–348. 10.1080/13543784.2020.1738383 32175781

[303] Cummings JL, Atri A, Feldman HH, Hansson O, Sano M, Knop FK, Johannsen P, León T et al (2025) Evoke and evoke+: design of two large-scale, double-blind, placebo-controlled, phase 3 studies evaluating efficacy, safety, and tolerability of semaglutide in early-stage symptomatic Alzheimer’s disease. Alzheimers Res Ther. 10.1186/S13195-024-01666-7 PMC11708093 39780249

[304] Ji C, Xue GF, Lijun C, Feng P, Li D, Li L, Li G, Hölscher C (2016) A novel dual GLP-1 and GIP receptor agonist is neuroprotective in the MPTP mouse model of Parkinson’s disease by increasing expression of BNDF. Brain Res 1634:1–11. 10.1016/j.brainres.2015.09.035 26453833

[305] Cummings JL, Atri A, Feldman HH, Hansson O, Sano M, Knop FK, Johannsen P, León T, Scheltens P (2025) evoke and evoke+: design of two large-scale, double-blind, placebo-controlled, phase 3 studies evaluating efficacy, safety, and tolerability of semaglutide in early-stage symptomatic Alzheimer’s disease. Alzheimer’s Research and Therapy 17. 10.1186/S13195-024-01666-7 PMC11708093 39780249

[306] Vijiaratnam N, Girges C, Auld G, McComish R, King A, Skene SS, Hibbert S, Wong A et al (2025) Exenatide once a week versus placebo as a potential disease-modifying treatment for people with Parkinson’s disease in the UK: a phase 3, multicentre, double-blind, parallel-group, randomised, placebo-controlled trial. Lancet 405:627–636. 10.1016/S0140-6736(24)02808-3 39919773

[307] Athauda D, Maclagan K, Skene SS, Bajwa-Joseph M, Letchford D, Chowdhury K, Hibbert S, Budnik N et al (2017) Exenatide once weekly versus placebo in Parkinson’s disease: a randomised, double-blind, placebo-controlled trial. Lancet 390:1664–1675. 10.1016/S0140-6736(17)31585-4 PMC5831666 28781108

[308] Badawi GA, Abd El Fattah MA, Zaki HF, El Sayed MI (2017) Sitagliptin and liraglutide reversed nigrostriatal degeneration of rodent brain in rotenone-induced Parkinson’s disease. Inflammopharmacology 25:369–382. 10.1007/s10787-017-0331-6 28258522

[309] Study Details | NCT02953665 | Safety and efficacy of liraglutide in Parkinson’s disease | ClinicalTrials.gov, (n.d.). https://clinicaltrials.gov/study/NCT02953665. Accessed 10 Apr 2026

[310] Hogg E, Wu T, Bresee C, Wertheimer J, Malatt C, Tan E, Pomeroy H, Nuno M et al (2022) A phase II, randomized, double-blinded, placebo-controlled trial of liraglutide in Parkinson’s disease. SSRN Electron J. 10.2139/SSRN.4212371

[311] Malatt C, Wu T, Bresee C, Hogg E, Wertheimer J, Tan E, Pomeroy H, Obialisi G et al (2022) Liraglutide improves non-motor function and activities of daily living in patients with Parkinson’s disease: a randomized, double-blind, placebo-controlled trial (P9-11.005). Neurology 98:3068. 10.1212/WNL.98.18_SUPPLEMENT.3068

[312] Saliev T, Singh PB (2025) Targeting senescence: a review of senolytics and senomorphics in anti-aging interventions. Biomolecules 15:860. 10.3390/BIOM15060860 40563501 PMC12190739

[313] Krzystyniak A, Wesierska M, Petrazzo G, Gadecka A, Dudkowska M, Bielak-Zmijewska A, Mosieniak G, Figiel I, Wlodarczyk J, Sikora E (2022) Combination of dasatinib and quercetin improves cognitive abilities in aged male Wistar rats, alleviates inflammation and changes hippocampal synaptic plasticity and histone H3 methylation profile. Aging 14:572–595. 10.18632/AGING.203835 PMC8833137 35042834

[314] Zou W, Pu T, Feng W, Lu M, Zheng Y, Du R, Xiao M, Hu G (2019) Blocking meningeal lymphatic drainage aggravates Parkinson’s disease-like pathology in mice overexpressing mutated α-synuclein. Transl Neurodegener 8:7. 10.1186/s40035-019-0147-y 30867902 PMC6396507

[315] Wang M, Ding F, Deng SY, Guo X, Wang W, Iliff JJ, Nedergaard M (2017) Focal solute trapping and global glymphatic pathway impairment in a murine model of multiple microinfarcts. J Neurosci 37:2870–2877. 10.1523/JNEUROSCI.2112-16.2017 28188218 PMC5354332

[316] Alessi DR, Sammler E (1979) LRRK2 kinase in Parkinson’s disease. Science 360(2018):36–37. 10.1126/science.aar5683 29622645

[317] Xie L, Kang H, Xu Q, Chen MJ, Liao Y, Thiyagarajan M, O’Donnell J, Christensen DJ et al (1979) Sleep drives metabolite clearance from the adult brain. Science 342:373–377. 10.1126/science.1241224 PMC3880190 24136970

[318] Zou W, Song Z, Guo Q, Liu C, Zhang Z, Zhang Y (2020) Intracranial pressure monitoring: epidemiological study of complementary approaches in traumatic brain injury. J Neurotrauma 37:1111–1121. 10.1089/neu.2019.6788

[319] Holth JK, Fritschi SK, Wang C, Pedersen NP, Cirrito JR, Mahan TE, Finn MB, Manis M et al (1979) The sleep-wake cycle regulates brain interstitial fluid tau in mice and CSF tau in humans. Science 363:80–884. 10.1126/science.aav2546 PMC6410369 30679382

[320] Fultz NE, Bonmassar G, Setsompop K, Stickgold RA, Rosen BR, Polimeni JR, Lewis LD (1979) Coupled electrophysiological, hemodynamic, and cerebrospinal fluid oscillations in human sleep. Science 366(2019):628–631. 10.1126/science.aax5440 PMC7309589 31672896

[321] Wu CH, Liao WH, Chu YC, Hsiao MY, Kung Y, Wang JL, Chen WS (2024) Very low-intensity ultrasound facilitates glymphatic influx and clearance via modulation of the TRPV4-AQP4 pathway. Advanced Science 11:2401039. 10.1002/ADVS.202401039;PAGE:STRING:ARTICLE/CHAPTER 39494466 PMC11653672

[322] Study Details | NCT05348785 | A study to learn about the safety of BIIB122 tablets and whether they can slow the worsening of early-stage Parkinson’s disease in adults between the ages of 30 and 80 | ClinicalTrials.gov, (n.d.). https://clinicaltrials.gov/study/NCT05348785. Accessed 20 June 2026

[323] Zhu B, Cao A, Chen C, Zhou W, Luo W, Gui Y, Wang Q, Xu Z, Wang J (2024) MMP-9 inhibition alleviates postoperative cognitive dysfunction by improving glymphatic function via regulating AQP4 polarity. Int Immunopharmacol 126. 10.1016/j.intimp.2023.111215 38000234

[324] Wang S, Wu D, Chen G, Yuan J, Zheng L, Huang X, Liu X, Kang Z et al (2026) Agomelatine targets aquaporin-4 polarization to rescue glymphatic dysfunction in Parkinson’s disease. Neurosci Bull. 10.1007/S12264-025-01543-9 PMC13158310 41251938

[325] Liu Z, Yang S, Song L, Zhang Y, Wan Y, Gan J, Wu N, Liu Z (2025) Tianqi pingchan granule promotes recovery of glymphatic system function in a rat model of L-DOPA-induced dyskinesia. J Tradit Complement Med 15:380–387. 10.1016/j.jtcme.2024.04.010 40677540 PMC12266283

[326] rui Sun Y, kun Lv Q, yu Xue X, Zhang Y, ying Shi L, le Sun M, Shen Y, jie Mao C, hui Wang L, Wang F, Liu JY, Liu CF (2026) Sleep deprivation accelerates Parkinson’s disease pathology by upregulating LAG3 in astrocytes and disrupting glymphatic system function. J Adv Res. 10.1016/J.JARE.2026.04.035 41980625

[327] Sun X, Dias L, Peng C, Zhang Z, Ge H, Wang Z, Jin J, Jia M et al (2024) 40 Hz light flickering facilitates the glymphatic flow via adenosine signaling in mice. Cell Discov. 10.1038/S41421-024-00701-Z PMC11300858 39103336

[328] Shi L, Xie J, Dai T, Zhang M, Yang T, Sheng L, Jin Q, Dai J (2026) Exercise-induced modulation of astrocyte in Alzheimer’s disease: mechanisms and therapeutic implications. Front Physiol 17:1827919. 10.3389/FPHYS.2026.1827919/FULL 42180831 PMC13189783

[329] Zhang J, Zhang J, Ding J, Zhang X, Wang Y, Wei W, Chai Y, Zhang S et al (2026) Reconstructing cerebral lymphatic clearance: an emerging target in the Alzheimer’s disease therapeutic pipeline. Alzheimer’s and Dementia 22:e71451. 10.1002/ALZ.71451;ISSUE:ISSUE:DOI PMC13183606 42151696

[330] Murdock MH, Yang CY, Sun N, Pao PC, Blanco-Duque C, Kahn MC, Kim TH, Lavoie NS et al (2024) Multisensory gamma stimulation promotes glymphatic clearance of amyloid. Nature 627:149–156. 10.1038/S41586-024-07132-6 PMC10917684 38418876

[331] Allen NJ, Bennett ML, Foo LC, Wang GX, Chakraborty C, Smith SJ, Barres BA (2012) Astrocyte glypicans 4 and 6 promote formation of excitatory synapses via GluA1 AMPA receptors. Nature 486:410–414. 10.1038/nature11059 22722203 PMC3383085

[332] McFarthing K, Buff S, Rafaloff G, Pitzer K, Fiske B, Navangul A, Beissert K, Pilcicka A et al (2024) Parkinson’s disease drug therapies in the clinical trial pipeline: 2024 update. J Parkinsons Dis 14:899–912. 10.3233/JPD-240272 PMC11307066 39031388

[333] Heiss JD, Lungu C, Hammoud DA, Herscovitch P, Ehrlich DJ, Argersinger DP, Sinharay S, Scott G et al (2019) Trial of magnetic resonance–guided putaminal gene therapy for advanced Parkinson’s disease. Mov Disord 34:1073–1078. 10.1002/mds.27724 PMC6642028 31145831

[334] Rocco MT, Akhter AS, Ehrlich DJ, Scott GC, Lungu C, Munjal V, Aquino A, Lonser RR, Fiandaca MS, Hallett M, Heiss JD, Bankiewicz KS (2022) Erratum: Long-term safety of MRI-guided administration of AAV2-GDNF and gadoteridol in the putamen of individuals with Parkinson’s disease. Mol Ther 30(12):3632–3638(S1525001622004919). 10.1016/j.ymthe.2022.08.003 PMC9734022 35957524

[335] Reisman SJ, Halabi D, Miller SE, Song L, Geraghty S, Sangvai N, Rice G, Safi A et al (2025) Comprehensive profiling of transcription factors for reprogramming human astrocytes to neuronal cells through endogenous CRISPR-based gene activation. BioRxiv. 10.1101/2025.10.11.681828

[336] Al-Dhahi A, Al-kuraishy HM, Albuhadily AK, Al-Gareeb AI, Abdelaziz AM, Alexiou A, Papadakis M, Alruwaili M et al (2025) The possible role of neurogenesis activators in temporal lobe epilepsy: state of art and future perspective. Eur J Pharmacol 998:177646. 10.1016/J.EJPHAR.2025.177646 40258399

[337] Giehrl‐Schwab J, Giesert F, Rauser B, Lao CL, Hembach S, Lefort S, Ibarra IL, Koupourtidou C, Luecken MD, Truong DJ, Fischer‐Sternjak J, Masserdotti G, Prakash N, Ninkovic J, Hölter SM, Vogt Weisenhorn DM, Theis FJ, Götz M, Wurst W (2022) Parkinson’s disease motor symptoms rescue by CRISPRa‐reprogramming astrocytes into GABAergic neurons. EMBO Mol Med 14 EMMM202114797. 10.15252/EMMM.202114797/FIGURES/7 PMC9081909 35373464

[338] Zhou H, Su J, Hu X, Zhou C, Li H, Chen Z, Xiao Q, Wang B et al (2020) Glia-to-neuron conversion by CRISPR-CasRx alleviates symptoms of neurological disease in mice. Cell 181:590-603.e16. 10.1016/j.cell.2020.03.024 32272060

[339] Song JJ, Oh SM, Kwon OC, Wulansari N, Lee HS, Chang MY, Lee E, Sun W et al (2017) Cografting astrocytes improves cell therapeutic outcomes in a Parkinson’s disease model. J Clin Invest 128:463. 10.1172/JCI93924 PMC5749514 29227284

[340] Oh S, Chang M, Song J, Rhee Y, Joe E, Lee H, Yi S, Lee S (2015) Combined Nurr1 and Foxa2 roles in the therapy of Parkinson’s disease. EMBO Mol Med 7:510–525. 10.15252/EMMM.201404610 25759364 PMC4492814

[341] Abdelaziz AM (2025) Alpha-synuclein drives NURR1 and NLRP3 inflammasome dysregulation in Parkinson’s disease: from pathogenesis to potential therapeutic strategies. Int Immunopharmacol 156:114692. 10.1016/J.INTIMP.2025.114692 40267723

[342] Francistiová L, Bianchi C, Di Lauro C, Sebastián-Serrano Á, de Diego-García L, Kobolák J, Dinnyés A, Díaz-Hernández M (2020) The role of P2X7 receptor in Alzheimer’s disease. Front Mol Neurosci 13:94. 10.3389/fnmol.2020.00094 32581707 PMC7283947

[343] Di Virgilio F, Dal Ben D, Sarti AC, Giuliani AL, Falzoni S (2017) The P2X7 receptor in infection and inflammation. Immunity 47:15–31. 10.1016/j.immuni.2017.06.020 28723547

[344] Ferrazoli EG, de Souza HDN, Nascimento IC, Oliveira-Giacomelli Á, Schwindt TT, Britto LR, Ulrich H (2017) Brilliant blue G, but not fenofibrate, treatment reverts hemiparkinsonian behavior and restores dopamine levels in an animal model of parkinson’s disease. Cell Transplant 26:669–677. 10.3727/096368917X695227 28403913 PMC5661224

[345] Carmo MRS, Simões AP, Fonteles AA, Souza CM, Cunha RA, Andrade GM (2014) ATP P2Y1 receptors control cognitive deficits and neurotoxicity but not glial modifications induced by brain ischemia in mice. Eur J Neurosci 39:614–622. 10.1111/EJN.12435 24304101

[346] Bhattacharya A, Wang Q, Ao H, Shoblock JR, Lord B, Aluisio L, Fraser I, Nepomuceno D et al (2013) Pharmacological characterization of a novel centrally permeable P2X7 receptor antagonist: JNJ-47965567. Br J Pharmacol 170:624–640. 10.1111/bph.12314 PMC3792000 23889535

[347] Burnstock G, Knight GE (2018) The potential of P2X7 receptors as a therapeutic target, including inflammation and tumour progression. Purinergic Signal 14:1–18. 10.1007/s11302-017-9593-0 29164451 PMC5842154

[348] Kopp R, Krautloher A, Ramírez-Fernández A, Nicke A (2019) P2X7 interactions and signaling – making head or tail of it. Front Mol Neurosci 12:183. 10.3389/fnmol.2019.00183 31440138 PMC6693442

[349] Coll RC, Robertson AAB, Chae JJ, Higgins SC, Muñoz-Planillo R, Inserra MC, Vetter I, Dungan LS et al (2015) A small-molecule inhibitor of the NLRP3 inflammasome for the treatment of inflammatory diseases. Nat Med 21:248–257. 10.1038/nm.3806 PMC4392179 25686105

[350] Gordon R, Albornoz EA, Christie DC, Langley MR, Kumar V, Mantovani S, Robertson AAB, Butler MS, Rowe DB, O’Neill LA, Kanthasamy AG, Schroder K, Cooper MA, Woodruff TM (2018) Inflammasome inhibition prevents -synuclein pathology and dopaminergic neurodegeneration in mice. Sci Transl Med 10 eaah4066. 10.1126/scitranslmed.aah4066 PMC6483075 30381407

[351] Brambilla R, Bracchi-Ricard V, Hu WH, Frydel B, Bramwell A, Karmally S, Green EJ, Bethea JR (2005) Inhibition of astroglial nuclear factor κB reduces inflammation and improves functional recovery after spinal cord injury. J Exp Med 202:145–156. 10.1084/jem.20041918 15998793 PMC2212896

[352] Villapol S (2018) Roles of peroxisome proliferator-activated receptor gamma on brain and peripheral inflammation. Cell Mol Neurobiol 38:121–132. 10.1007/s10571-017-0554-5 28975471 PMC5776063

[353] Serafino A, Cozzolino M (2022) The Wnt/β-catenin signaling: a multifunctional target for neuroprotective and regenerative strategies in Parkinson’s disease. Neural Regen Res 18:306. 10.4103/1673-5374.343908 PMC9396482 35900408

[354] Anfinogenova ND, Quinn MT, Schepetkin IA, Atochin DN (2020) alarmins and c-Jun N-terminal kinase (JNK) signaling in neuroinflammation. Cells 9:2350. 10.3390/CELLS9112350 33114371 PMC7693759

[355] Kirkley KS, Popichak KA, Hammond SL, Davies C, Hunt L, Tjalkens RB (2019) Genetic suppression of IKK2/NF-κB in astrocytes inhibits neuroinflammation and reduces neuronal loss in the MPTP-probenecid model of Parkinson’s disease. Neurobiol Dis 127:193. 10.1016/J.NBD.2019.02.020 30818064 PMC6588478

[356] Jiang T, Hoekstra J, Heng X, Kang W, Ding J, Liu J, Chen S, Zhang J (2015) P2X7 receptor is critical in α-synuclein-mediated microglial NADPH oxidase activation. Neurobiol Aging 36:2304–2318. 10.1016/J.NEUROBIOLAGING.2015.03.015 25983062

[357] Baidya F, Bohra M, Datta A, Sarmah D, Shah B, Jagtap P, Raut S, Sarkar A et al (2021) Neuroimmune crosstalk and evolving pharmacotherapies in neurodegenerative diseases. Immunology 162:160–178. 10.1111/IMM.13264 PMC7808166 32939758

[358] Ramos-Gonzalez P, Mato S, Chara JC, Verkhratsky A, Matute C, Cavaliere F (2021) Astrocytic atrophy as a pathological feature of Parkinson’s disease with LRRK2 mutation. Npj Parkinson’s Disease 7(1):1–11. 10.1038/s41531-021-00175-w PMC8009947 33785762

[359] Cyske Z, Gaffke L, Rintz E, Wiśniewska K, Węgrzyn G, Pierzynowska K (2024) Molecular mechanisms of the ambroxol action in Gaucher disease and GBA1 mutation-associated Parkinson disease. Neurochem Int 178:105774. 10.1016/J.NEUINT.2024.105774 38797393

[360] Streubel-Gallasch L, Giusti V, Sandre M, Tessari I, Plotegher N, Giusto E, Masato A, Iovino L et al (2021) Parkinson’s disease–associated LRRK2 interferes with astrocyte-mediated alpha-synuclein clearance. Mol Neurobiol 58:3119. 10.1007/S12035-021-02327-8 PMC8257537 33629273

[361] Duret LC, Nagoshi E (2025) The intertwined relationship between circadian dysfunction and Parkinson’s disease. Trends Neurosci 48:62–76. 10.1016/J.TINS.2024.10.006 39578132

[362] Gan L, Vargas MR, Johnson DA, Johnson JA (2012) Astrocyte-specific overexpression of Nrf2 delays motor pathology and synuclein aggregation throughout the CNS in the alpha-synuclein mutant (A53T) mouse model. J Neurosci 32:17775–17787. 10.1523/JNEUROSCI.3049-12.2012 23223297 PMC3539799

[363] Liddell JR (2017) Are astrocytes the predominant cell type for activation of Nrf2 in aging and neurodegeneration?. Antioxidants 6:65. 10.3390/ANTIOX6030065 PMC5618093 28820437

[364] Hertz L (2004) The astrocyte-neuron lactate shuttle: a challenge of a challenge. J Cereb Blood Flow Metab 24:1241–1248. 10.1097/00004647-200411000-00008 15545919

[365] Yamagata K (2022) Lactate supply from astrocytes to neurons and its role in ischemic stroke-induced neurodegeneration. Neuroscience 481:219–231. 10.1016/J.NEUROSCIENCE.2021.11.035 34843897

[366] Cui J, Guo X, Li Q, Song N, Xie J (2020) Hepcidin-to-ferritin ratio is decreased in astrocytes with extracellular alpha-synuclein and iron exposure. Front Cell Neurosci 14:523399. 10.3389/FNCEL.2020.00047/BIBTE PMC7075942 32210768

[367] Song N, Wang J, Jiang H, Xie J (2018) Astroglial and microglial contributions to iron metabolism disturbance in Parkinson’s disease. Biochimica et Biophysica Acta (BBA) - Molecular Basis of Disease 186:967–973. 10.1016/J.BBADIS.2018.01.008 29317336

[368] Peterson AL, Nutt JG (2008) Treatment of Parkinson’s disease with trophic factors. Neurotherapeutics 5:270–280. 10.1016/J.NURT.2008.02.003 18394569 PMC5084169

[369] Gash DM, Gerhardt GA, Bradley LH, Wagner R, Slevin JT (2020) GDNF clinical trials for Parkinson’s disease: a critical human dimension. Cell Tissue Res 382:65–70. 10.1007/S00441-020-03269-8 32830288

[370] Zhang L, Yin JC, Yeh H, Ma NX, Lee G, Chen XA, Wang Y, Lin L et al (2015) Small molecules efficiently reprogram human astroglial cells into functional neurons. Cell Stem Cell 17:735. 10.1016/J.STEM.2015.09.012 PMC4675726 26481520

[371] Liu H, Mao H, Ouyang X, Lu R, Li L (2024) Intercellular mitochondrial transfer: the novel therapeutic mechanism for diseases. Traffic 25:e12951. 10.1111/TRA.12951 39238078

[372] Jain R, Begum N, Tryphena KP, Singh SB, Srivastava S, Rai SN, Vamanu E, Khatri DK (2023) Inter and intracellular mitochondrial transfer: future of mitochondrial transplant therapy in Parkinson’s disease. Biomed Pharmacother 159:114268. 10.1016/J.BIOPHA.2023.114268 36682243

[373] Zhang Y, Tan F, Xu P, Qu S (2016) Recent advance in the relationship between excitatory amino acid transporters and Parkinson’s disease. Neural Plast. 10.1155/2016/8941327 26981287 PMC4769779

[374] de Rus Jacquet A, Alpaugh M, Denis HL, Tancredi JL, Boutin M, Decaestecker J, Beauparlant C, Herrmann L, Saint-Pierre M, Parent M, Droit A, Breton S, Cicchetti F (2023) The contribution of inflammatory astrocytes to BBB impairments in a brain-chip model of Parkinson’s disease. Nat Commun 14(1):1–21. 10.1038/s41467-023-39038-8 PMC10282096 37339976

[375] Argaw AT, Asp L, Zhang J, Navrazhina K, Pham T, Mariani JN, Mahase S, Dutta DJ et al (2012) Astrocyte-derived VEGF-A drives blood-brain barrier disruption in CNS inflammatory disease. J Clin Invest 122:2454–2468. 10.1172/JCI60842 PMC3386814 22653056

